# Can Provence Flora Offer Effective Alternatives to Widely Used Medicinal Plants? A Comparative Study of Antioxidant Activity and Chemical Composition Using Molecular Networking

**DOI:** 10.3390/molecules30092072

**Published:** 2025-05-07

**Authors:** Clémentine Achard-Baccati, Elnur Garayev, Charifat Saïd Hassane, Célia Breaud, Eldar Garaev, Myriam Bertolotti, Fathi Mabrouki, Sok-Siya Bun-Llopet, Béatrice Baghdikian

**Affiliations:** 1 IMBE, Aix Marseille Univ, Avignon Univ, CNRS 7263, IRD 237, 27 Blvd Jean Moulin, Service of Pharmacognosy, Faculty of Pharmacy, 13385 Marseille, France; clementine.baccati@univ-amu.fr (C.A.-B.); elnur.garayev@univ-amu.fr (E.G.);; 2 Department of General and Toxicological Chemistry, Azerbaijan Medical University, Baku AZ1001, Azerbaijan

**Keywords:** Provence flora, molecular networking, chemical composition, UHPLC-HRMS/MS, antioxidant, phenolics, post-column assays

## Abstract

This study compares the antioxidant properties and phytochemical profiles of three pairs of widely used medicinal plant species to their counterparts from Provence, France: *Arnica montana* with *Pentanema montanum* (formerly known as *Inula montana), Helichrysum italicum* with *Helichrysum stoechas*, and *Satureja hortensis* with *Satureja montana*. Phytochemical composition has been investigated using UHPLC-HRMS/MS and molecular networking, revealing chemical profiles dominated by phenylpropanoids and flavonoids, with lignans, sesquiterpene lactones, or polyketides aside. Well-plate DPPH/ABTS assays were used to evaluate the antioxidant activity of extracts, and post-column assays were used to identify antioxidant compounds. The three Provence species demonstrated comparable or superior antioxidant activities to their counterparts, primarily attributed to phenolic compounds such as mono- and di-caffeoylquinic acids, quercetagetin-7-O-glucoside, and myricetin acetylhexoside. These findings show the potential of Provence species to be substituted for some overharvested medicinal plants. This research supports biodiversity conservation while promoting the integration of these local species into pharmaceutical, nutraceutical, cosmetic, and food industries.

## 1. Introduction

In recent years, the global demand for herbal products, especially those with antioxidant properties, has increased due to their important use in health, cosmetics, and industrial applications. Antioxidants are essential agents to reduce oxidative stress, implicated in various pathological disorders such as inflammation, cancer, and others [1,2,3]. Herbal products, rich in bioactive phytochemicals like phenolic compounds, are particularly valuable in the food industry to extend shelf life by preventing lipid oxidation for example [4,5]. Furthermore, in cosmetics, antioxidant compounds reduce oxidative stress, helping to prevent skin aging and stabilize formulations, thus improving their preservation and efficacy [6,7].

The growing demand for medicinal plants involves significant sustainability challenges. With more than 20% of medicinal plant species worldwide considered under threat, and approximately 90% of European medicinal plants harvested in the wild [8,9], there are raising concerns about natural resource depletion.

Overexploitation of endangered or rare plants for industrial use has long been a concern, leading to many conservation efforts [10,11,12]. Various strategies have been proposed, such as small-scale cultivation, sustainable harvesting, in situ and ex situ conservation, and substitution of endangered plants with closely related species offering similar benefits. For instance, the Chinese medicinal plant *Salvia miltiorrhiza* is often substituted with other species from the same genus due to cultivation difficulties [13], while *Arnica chamissonis* is used instead of *A. montana* due to the latter’s challenging cultivation requirements [14,15,16]. Alternative substitutes to rare medicinal plants can be chosen based on local traditional knowledge or based on chemosystematics and pharmaco-phylogeny studies [8,17,18,19,20]. Indeed, numerous studies showed that the chemical compounds responsible for the medicinal properties of plants are linked to their phylogenetic classification [21,22,23].

The Mediterranean region, and especially Provence, is a recognized biodiversity hotspot and plays a central role in the global production of medicinal and aromatic plants [24,25]. Many plant species from this region have been traditionally used for their nutritional and therapeutic properties [26].

This study aims to evaluate whether selected medicinal plants from Provence can be used as sustainable alternatives to widely used species with similar therapeutic applications. The chosen plant species from Provence meet three criteria (*i*) documented ethnobotanical uses in traditional medicine to treat external ailments, such as wounds, burns, or bruises, (*ii*) non-endangered conservation status to ensure sustainability, and (*iii*) growing demand for their widely used counterparts. Therefore, we compare the phytochemical composition and antioxidant activity of three plant pairs, each of them composed of a widely known medicinal species and a taxonomically related species from Provence: *Arnica montana* vs. *Pentanema montanum* (formerly known as *Inula montana)*, *Helichrysum italicum* vs. *Helichrysum stoechas*, and *Satureja hortensis* vs. *Satureja montana*.

In Provence, *Pentanema montanum* is used for healing bruises, muscular pain, and burns, similar to the officinal species *Arnica montana*, which is administered orally as a tincture or applied topically as an ointment [27,28,29,30]. Likewise, *Helichrysum stoechas* is widely employed in Provence in traditional medicine and in cosmetics for its anti-inflammatory and regenerative properties, comparable to the use of *Helichrysum italicum* for the same purposes [31,32,33,34,35,36]. Similarly, *Satureja montana* is recognized in Mediterranean traditional medicine for its antimicrobial and analgesic properties, alongside the officinal use of *Satureja hortensis*, with both species also serving as aromatic herbs [37,38,39].

Antioxidant activity was chosen as a preliminary screening criterion due to its fundamental role in protecting cells against oxidative stress, a major factor involved in skin damage. Widely used in cosmetics for its ability to neutralize free radicals and enhance skin protection, antioxidant activity also plays a key role in various biological functions, including anti-inflammatory and wound-healing properties. Antioxidant activity was assessed through DPPH and ABTS reagents, both in microplates and post-column On-Line techniques, to directly identify bioactive compounds. Metabolomic profiling based on Ultra-High Performance Liquid Chromatography coupled with High-Resolution Mass Spectrometry (UHPLC-HRMS/MS), in both positive and negative ionization modes, was combined with molecular networking for metabolite annotation. 

By integrating phytochemical analysis with antioxidant evaluation, this research seeks to determine the potential of Provence species as reliable substitutes, promoting biodiversity conservation and the preservation of traditional knowledge [40].

## 2. Results and Discussion

### 2.1. Chemical Composition of the Extracts

In untargeted metabolomics studies, computational approaches for dereplication are increasingly popular, especially for their efficiency in processing large datasets [41]. Different types of tools have been developed to describe and investigate metabolomes, such as classification predictions and molecular networking.

#### 2.1.1. Chemical Classification Predictions

Some computational tools aim to automatically predict the structural classification of natural products [42], such as CANOPUS, specifically designed for the classification of chemical compounds based on their mass spectrometry data. The CANOPUS tool relies on its own neural network classification system, but can also refer to external classification systems, such as the NPClassifier ontology based on 7 metabolic pathways, containing 70 superclasses and 672 classes [43].

**Figure 1:**
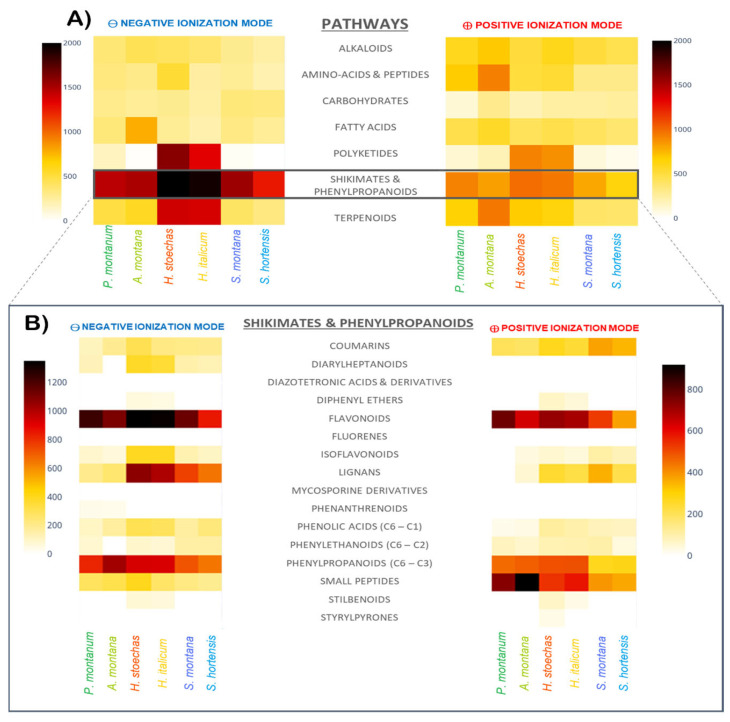
Heatmaps showing metabolites repartition according to their metabolic pathways (**A**) and superclasses among “Shikimates and Phenylpropanoids” pathway (**B**).

Heatmaps can be used to visualize classification predictions, showing metabolite relative abundance calculated from the square root of their peak areas for both negative and positive ionization modes using a Python (3.10) script freely available on GitHub [44]). Darker colors represent higher abundance, while lighter yellow indicates lower or no abundance. Figure 1 displays heatmaps with the relative abundance of metabolites grouped either by metabolic pathway (Figure 1A) or by superclass (Figure 1B). Most of the metabolites are derived from the “Shikimates and Phenylpropanoids” pathway, regardless of the ionization mode. Both species of the *Helichrysum* genus also show high amounts of metabolites from polyketides and terpenoids pathways, which is in accordance with the literature [45,46].

Focusing on features from the “Shikimates and Phenylpropanoids” metabolic pathway, flavonoids and phenylpropanoids (C6–C3) represent the most abundant superclass for every species of the sampling (Figure 1B). However, the repartition of classes of flavonoids differs between pairs (Figure S1 of Supplementary Material). The *Arnica* pair shows profiles with dominance of flavones and flavonols. The species of the *Helichrysum* pair show similar profiles dominated by flavonols in both ionization modes, and with high amounts of chalcones, only in the positive ionization mode. The chemical profiles of the *Satureja* pair are quite similar, with high amounts of flavones, flavonols, and flavanones, the latter being prominent in *S. montana* extracts.

In addition to flavonoids and phenylpropanoids, the *Arnica* pair also exhibits high amounts of small peptides (mostly amino acids), mainly observable in positive ionization mode, while the *Helichrysum* pair shows noticeable amounts of lignans, in negative ionization mode, and small peptides in positive ionization mode, both rarely described in the genus [47].

On the basis of classification predictions, each pair appears to have a common chemical profile in terms of metabolite structures. To visualize and analyze in greater detail the similarities and differences of chemical composition within each pair, molecular networks were then used.

#### 2.1.2. Molecular Networking

Molecular Networks (MNs) approaches, linking mass spectra of metabolites based on their fragmentation patterns, are used to visualize, characterize, and identify metabolites using mass spectrometry-based techniques [48]. The combination of MN with feature detection and dereplication tools, such as the FBMN tool from the GNPS platform, allows the incorporation of MS^1^ information and the automatic confrontation of experimental MS^2^ spectra with spectral libraries, facilitating the annotation [49,50].

MNs in both negative and positive ionization modes were created using the GNPS platform tool FBMN to visualize the chemical space of the samples and easily dereplicate metabolites. The negative MN is made of 2223 nodes, while the positive MN is made of 2178 nodes. In these two MNs, nodes represent feature ions, labeled with their MZmine feature IDs. Node cores are in pie chart shape, indicating the relative abundance of the feature in the species of the sampling, while the external color represents the metabolic pathway prediction of the CANOPUS tool. Features with round-rectangle contours correspond to the bioactive compounds detected with the post-column assays (presented hereafter). The node size depends on the sum of the precursor ion intensity.

A total of 228 compounds have been annotated (Table S1 of Supplementary Material) with the help of GNPS, SIRIUS, and timaR tools, and their putative identification confidence were classified according to Schymanski et al. confidence levels: (L1) for confirmed structures with reference standards matching of MS, MS2 data and retention time, (L2) for probable structures with (L2a) for spectra matching with literature or experimental library and (L2b) for diagnostic of structure using MS/MS fragments or ionization behavior, with no literature confirmation, (L3) for tentative identifications, (L4) for unequivocal molecular formula without structure [51].

**Figure 2:**
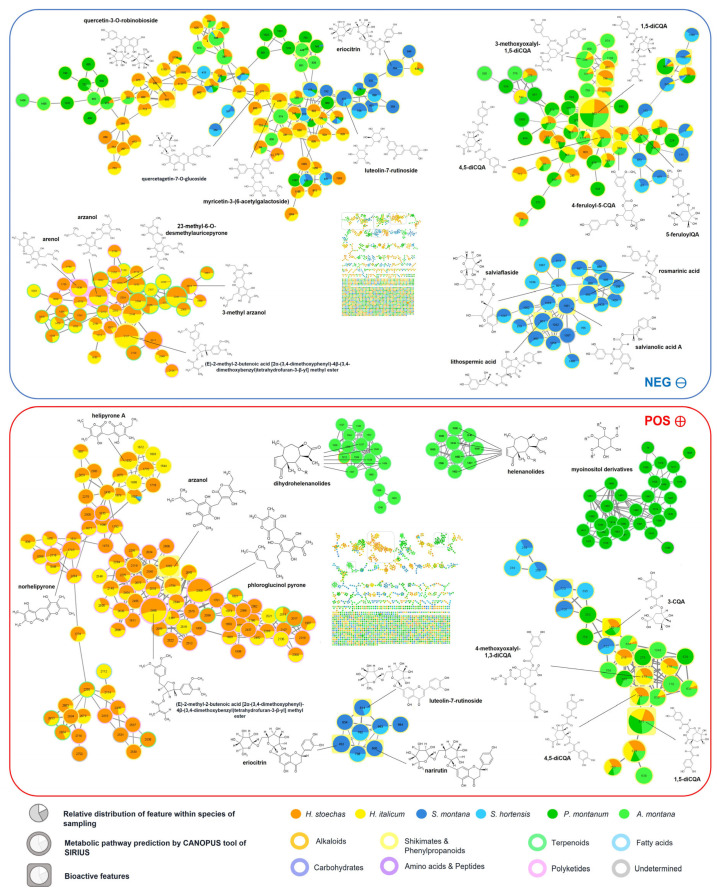
Molecular networks in negative (blue) and positive (red) ionization modes, with some highlighted clusters and annotated metabolites.

Figure 2 exhibits several large clusters composed of flavonoids widely distributed across the plant kingdom, alongside numerous smaller clusters composed of features specific to certain species or genera.

#### 2.1.3. Arnica Pair

Caffeoylquinic acid (CQA) derivatives, belonging to the phenylpropanoids superclass, are often described as major compounds in many Asteraceae species [52]. They represent the main constituents of both *A. montana* and *P. montanum* extracts, especially 3-CQA and 1,5-diCQA isomers [53,54,55]. Methoxyoxaloyl-diCQA isomers (ID− 598, 727, 798) were detected in *A. montana* extracts and in both *Helichrysum* extracts in accordance with the literature [55,56,57], showing a characteristic fragment at *m/z* 395.0989 corresponding to [M − caffeoyl − COOH], along with caffeoyl and dicaffeoyl fragments [35,58,59]. Moreover, a methoxyoxaloyl-triCQA (ID− 1108) is only observed in *A. montana* extract. Hexaric acid caffeoyl-derivatives, such as tri-(ID− 583 and 743) and tetra-(ID− 1043) caffeoyl hexaric acids, were only present in *P. montanum* extracts. To our knowledge, these compounds are described for the first time in *P. montanum*, but were already known in some species from the Inuleae tribe [60,61,62]. Although both species share several flavonoids, such as hispidulin (ID− 1173) and methoxy-myricetin-3-*O*-hexoside (ID− 468), the major part of the flavonoids are species-specific. Flavonols as quercetin 3-*O*-glucuronide (ID− 374), or kaempferol-3-*O*-glucoside (ID− 588) and quercetin-3-*O*-glucoside (ID− 391) are the major flavonoids in *A. montana*; whereas flavones as nepetin (ID− 1016), also called 6-methoxyluteolin, nepetin-7-glucoside (ID− 527), jaceoside (ID− 804) and luteolin (ID− 965) are the major flavonoids in *P. montanum*.

Besides flavonoids and phenylpropanoids, sesquiterpene lactones (SLs) represent an important class of metabolites in both *A. montana* and *P. montanum,* detected only with the positive ionization mode. These compounds are well-known as chemotaxonomic markers of the tribes in the Asteraceae family [63,64]. SLs result from a cyclical structure along with a fused α-methylene-γ-lactone ring [65]. Derivatives of helenalin and dihydrohelenalin are pseudo-guaianolides characteristic of *A. montana* [66,67,68,69,70]. This subclass of SLs presents a characteristic lactone ring fused with seven and five-membered rings, with a methyl group in the C-5 or C-10 position [71,72]. Helenalin esters share a characteristic fragment at *m/z* 245.1180 in positive ionization mode, while dihydrohelenalin esters share a common fragment at *m/z* 247.1329. Both characteristic fragments result from the elimination of carboxylic acids from C-6 of the esters [67]. Among those, dihydrohelenalin (ID+ 395), its tigloyl ester (ID+ 1459), and tigloyl ester of helenalin (ID+ 1493) are the most abundant. On the other hand, *P. montanum* extracts contain a germacranolide SL with a ten-membered ring costunolide (ID+ 1678), and an eudesmanolide SL with a six-membered ring alantolactone (ID+ 1619), as reported in scientific literature [27,73,74]. Moreover, several myoinositol derivatives are exclusively found in *P. montanum* extracts, such as myoinositol,1,5-diangelate-4,6-diacetate (ID+ 1361), myoinositol,1,6-diangelate-4,5-diacetate (ID+ 1380), myoinositol-1-angelate-4,5-diacetate-6-(2-methylbutyrate) (ID+ 1451) and myoinositol-1-angelate-4,5-diacetate-isovalerate (ID+ 1455).

#### 2.1.4. Helichrysum Pair

Both of the *Helichrysum* species showed similar profiles with numerous phenylpropanoids and flavonoids, but also polyketides, especially alpha-pyrones and other phloroglucinol derivatives, as described in the literature [45,75]. Some CQA derivatives, as 1,5-diCQA (ID− 562), 3,5-diCQA (ID− 707), and 1-methoxyoxaloyl-4,5-diCQA (ID− 798), figure among the major compounds of both *Helichrysum* species. Flavonoids, and especially flavonols, are widely represented: quercetin glycosides derivatives, such as quercetagetin-7-*O*-glucoside (ID− 271), quercetin-3-*O*-robinobioside (ID− 960) and quercetin-3-*O*-glucoside (ID− 391), or kaempferol and myricetin glycosides derivatives, as tiliroside (ID− 1080) or myricetin-acetyl- (ID− 436) and malonyl- (ID− 438) hexosides. These flavonoids have already been described as major components in these two species [58,76].

Several clusters specific to both *Helichrysum* species are composed of prenylated phloroglucinol pyrones with common ionization fragments in negative ionization mode, depending on their substituents. Pyrones with methyl and prenyl groups, such as arenol (ID− 1638) and arzanol (ID− 1746), share fragments at *m/z* 235.0972 and 247.0973; while pyrones with methyl and geranyl groups exhibit fragments at *m/z* 303.1606 and 315.1605, such as several unfully determined pyrones ethylpyrone (with ID− 2017, 2107, 2241) and those bearing methyl and hydroxygeranyl groups at *m/z* 319.1547 and 331.1546 (ID− 1658, 1781, 2001). Arzanol, 3-methylarzanol (ID− 1869), arenol, and helipyrone A (ID− 1730) are major compounds of both species, in accordance with the literature [34,75,77,78,79,80].

The differences between *H. stoechas* and *H. italicum* chemical compositions are primarily quantitative rather than qualitative, consisting of the same compounds but in varying amounts (Figure S2 of Supplementary Material). For instance, compounds as everlastoside L (ID− 769), heliarzanol (ID− 1709), 3-methylarzanol (ID− 1869), 2b-Isopropyl-3-methyl-8-(3′,3′-dimethylallyl-5,7-dihydroxychroman-4-one (ID− 1732), santinol A1 (ID+ 2438), arenol C (ID+ 2276) or some unidentified pyrones derivatives (ID− 1708, 1732), associated signals are twice to four times higher in *H. stoechas* extracts than in *H. italicum*.

#### 2.1.5. Satureja Pair

Both of the *Satureja* species contain mainly phenylpropanoids, flavonoids, and lignans, in concordance with the literature [38,81,82,83,84,85].

The *Satureja* pair exhibits specific phenylpropanoids, such as rosmarinic, salvianolic, and lithospermic acids, resulting from the condensation of caffeic acid units and salvianic acid, quite common in the Lamiaceae family, which were not found in the two Asteraceae pairs. Among them, rosmarinic acid (ID− 685), lithospermic acid A (ID− 861), and salvianolic acid B (ID− 917) are the major compounds described in both *Satureja* species [38,81]. Salvianolic acid A (ID− 1051) figures among the major components of *S. montana* extracts but is absent in *S. hortensis* extract. Formed by the condensation of salvianic acid and two caffeic acid units, salvianolic acid A is described as a potential derivative of different lithospermic acids, themselves derived from rosmarinic acid and its dimer, called salvianolic acid B [86]. Salvianolic acid B (ID− 917), twice as prevalent in *S. montana* as in *S. hortensis,* is characterized by a pseudo-molecular ion of *m/z* of 717.1456. The latter ion yields fragments at *m/z* of 519.0943, resulting from the loss of salvianic acid, and 357.0622, resulting from the loss of two caffeic acid moieties [87]. Lithospermic acid A isomers (ID− 861, 999) share ions with *m/z* at 537.1040, and a characteristic fragment at *m/z* 493.1152, resulting from the loss of an 8”-carboxyl group [87,88]. An undetermined rosmarinic acid derivative (ID− 189) is twice as abundant in *S. hortensis* as in *S. montana.*

Most of the flavonoids found in these two *Satureja* species are from the flavones superclass; for instance, diosmetin-7-rutinoside (ID− 720), luteolin-7-rutinoside (ID− 433), and luteolin-7-glucuronide (ID− 405). Salvigenin (ID+ 1645) is a flavone that is more prominent in *S. hortensis* than in *S. montana* extracts. Some flavanones are also present in both *Satureja* species, such as naringenin (ID− 1082), hesperidin (ID− 698), and eriodictyol (ID− 845). Some rutinoside-derived flavanones are quite abundant in *S. montana* while in traces in *S. hortensis*, such as eriodictyol-7-rutinoside (ID− 452) and naringenin-7-*O*-rutinoside (ID− 600). Among flavonols, quercetin-3-alloside (ID− 299) is more abundant in *S*. *hortensis* than in *S. montana.*

### 2.2. Antioxidant Activity Assessment

#### 2.2.1. Total Phenolic and Flavonoid Contents

Polyphenolic compounds, especially flavonoids, are often described as the main phytochemicals responsible for antioxidant activity in plant extracts [89].

Phenolic contents of the sampling ranged from 12.64 ± 3.13 mg/100 mg to 21.44 ± 1.65 mg/100 mg, while flavonoid contents ranged from 4.62 ± 0.44 mg/100 mg to 15.01 ± 1.32 mg/100 mg (Table 1). The *Helichrysum* species exhibited the highest total phenolic (TPC) and flavonoid (TFC) contents, with flavonoids representing over 70% of their phenolic composition, as evidenced by their notably high TFC/TPC ratios. On the contrary, *Satureja* species showed the lowest TFC/TPC ratios (<30%), indicating a dominance of non-flavonoid phenolic compounds.

#### 2.2.2. DPPH and ABTS Well-Plate Assays

The EC_50s_ of the six studied species are presented in Table 1, along with their TPC, TFC, and TFC/TPC ratios. All studied extracts were active. The *Helichrysum* extracts showed the most intense activity according to both DPPH and ABTS assays, with EC_50_ of 17.21 ± 2.38 µg/mL and 3.81 ± 0.75 µg/mL, respectively, with statistically comparable values between the two species. These results are consistent with their high TPC, TFC, and TFC/TPC ratios, underlining the role of flavonoids in their antioxidant activity. Although TPC, TFC, and TFC/TPC values are similar inside the *Arnica* pair, *P. montanum* EC_50s_ are half those of *A. montana*, suggesting non-flavonoid phenolic compounds mediate their antioxidant effects. In the same manner, the statistically comparable antioxidant activity of the two *Satureja* species comes from non-flavonoid phenolic compounds, with less than 30% of the phenolics being flavonoids, while these two species exhibited comparable activity to *Helichrysum* species in both assays.

Based on these two assays, the Provence species of each pair exhibit antioxidant activities that are either comparable to or even superior to those of their widely used relative.

#### 2.2.3. DPPH/ABTS-On-Line-UHPLC Assays

**Figure 3:**
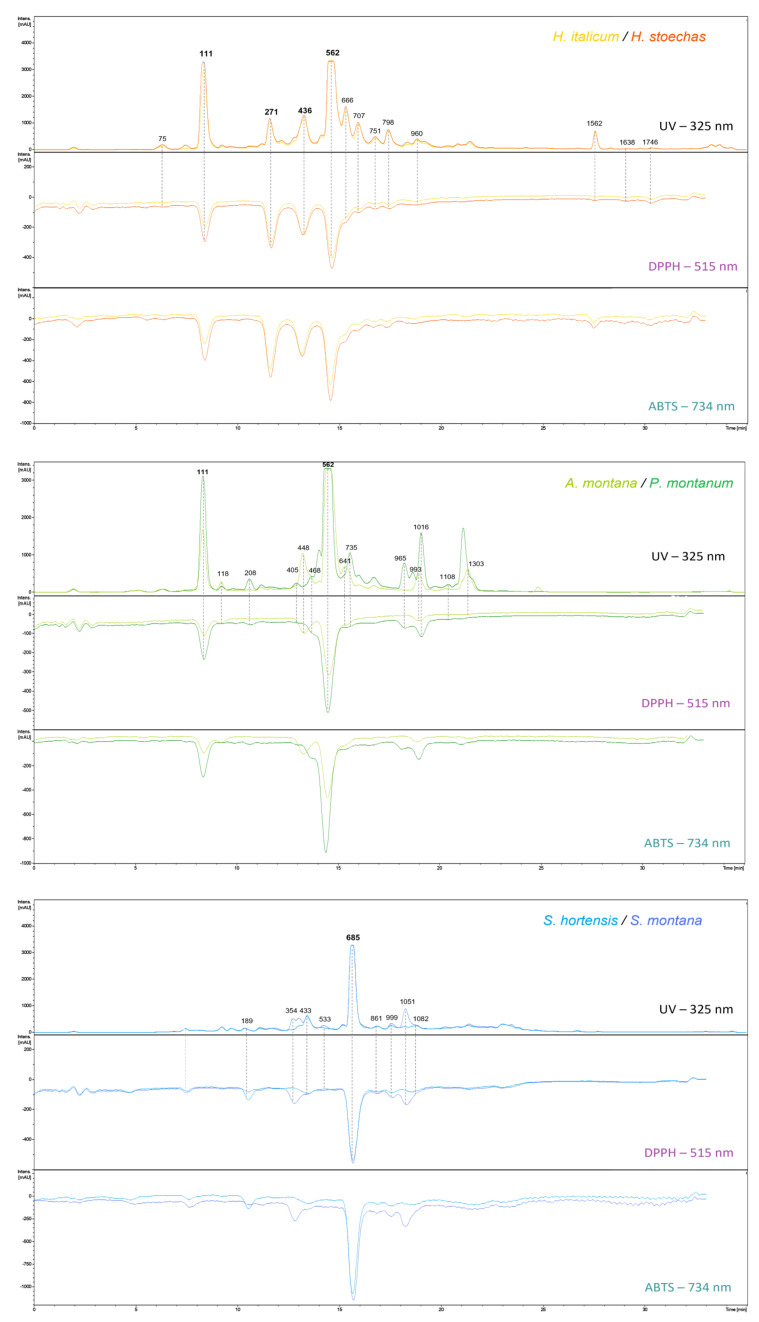
UV-VIS chromatograms of post-column DPPH/ABTS On-Line assays grouped by pair with metabolites identified by their ID in negative ionization mode, detailed below in Table 2.

Combined UV chromatograms with positive signals (at 325 nm) and negative signals (at 515 and 734 nm) are represented in Figure 3. The putative identifications of antioxidant compounds are presented in Table 2, and compounds are designated by their MZmine ID in negative mode in brackets. Among all detected analytes, 34 exhibited antiradical scavenging activity towards both DPPH and ABTS, most of them being phenolic compounds, with 9 phenylpropanoids and 19 flavonoids. In addition, 3 lignans, 2 aromatic polyketides, and one alkaloid figure among the bioactive compounds.

Nine of the detected radical scavenging compounds were phenylpropanoids derived from cinnamic acid, known for their antioxidant activities [90]. Caffeic acid (118) figures among radical scavenging compounds and is present in every species in this study. 

Chlorogenic acids, positional isomers of monoacyl-caffeoylquinic acid, are particularly known for their radical scavenging capacity [91]. These isomers, only found in Asteraceae species in this study, share a characteristic caffeoyl fragment ion at *m/z* 191.0571 in negative mode, while diCQA exhibits both caffeoyl and dicaffeoyl fragment ions at *m/z* 191.0571 et 353.0895. Two caffeoylquinic acid isomers, with a C_16_H_18_O_9_ molecular formula, are described here as antioxidant compounds: 3-CQA (ID− 75), or neochlorogenic acid, and 5-CQA (ID− 111), or chlorogenic acid. Neochlorogenic acid contributes to the antioxidant activity observed for both *Helichrysum* and *Arnica* pairs, while chlorogenic acid only contributes to the antioxidant activity in the *Arnica* pair. The annotation was based on fragment ion abundances and order of elution in the literature, and the 5-CQA identity was confirmed by comparison of a standard injected under the same chromatographic conditions [92]. The same approach was used to distinguish the positional isomers of diCQAs, leading to the annotation of 1,3-diCQA (ID− 208), 3,4-diCQA (ID− 498), 1,5-diCQA (ID− 562), which were confirmed by comparison with the standard and 3,5-diCQA (ID− 707) in order of elution [93]. The 1,5-diCQA isomer is the major compound responsible for the antioxidant activity in species from *Helichrysum* and *Arnica* pairs, while 3,5-diCQA and 1,3-diCQA are minor contributors, and 3,4-diCQA did not figure among antioxidant compounds. According to UV data at 325 nm, 5-CQA, and 1,5-diCQA are 2.5 times more abundant in *P. montanum* extract. This difference in concentration could explain the observed difference in activity. The significantly higher levels of active compounds provide a strong argument for considering this Provence species as a promising source of antioxidants. CQA derivatives are known antioxidant agents, and diCQA isomers exhibit stronger antioxidant activity than CQA isomers. In scientific literature, the EC_50_ of diCQA isolated isomers range between 7.5–9.5 µg/mL and 67.3–77.6 µg/mL for DPPH and ABTS assays, respectively, while between 13.2–13.8 µg/mL and 87.5–91.5 µg/mL for CQA isomers [94,95].

Some methoxylated derivatives of di- and tri-CQA, such as 4-methoxyoxaloyl-1,3-diCQA (ID− 798) and 3-methoxyoxaloyl-1,4,5-triCQA (ID− 1108), also contribute to the antioxidant activity. Another cinnamic acid derivative showing strong antioxidant activity is rosmarinic acid (ID− 685), only present in the *Satureja* species, with diagnostic fragments of *m/z* 161.0252 and 197.0464 [96]. This compound has already been described as a strong antioxidant compound, with EC_50_ values between 0.57 and 28.39 µg/mL in DPPH assays, depending on the authors [97,98,99]. Finally, three phenylpropanoid derivatives, sometimes classified as lignans, participate in the antioxidant activity of the *Satureja* species: lithospermic acid A (ID− 861), salvianolic acid B derivative (ID− 999), and salvianolic acid A (ID− 1051). These compounds were not found in any of the Asteraceae species.

Nineteen of the compounds with scavenging activity were flavonoids. Among those, three flavanones: eriodictyol-7-rutinoside (ID− 354) and naringenin-7-rutinoside (ID− 533) are specific to *Satureja* species, and naringenin (ID− 1082). These three flavanones share a fragment ion at *m/z* 151.0042, characteristic of the loss of the B ring of the flavanone and a C_2_H_2_O fragment of the precursor flavanone ion [100].

Five flavones show antioxidant activity: hispidulin-4-glucoside (ID− 735), luteolin (ID− 965), and three luteolin derivatives, luteolin-7-O-glucuronide (ID− 405), luteolin-7-O-diglucuronide (ID− 254), and 6-methoxyluteolin (ID− 1016), also called nepetin. Luteolin, nepetin, and hispidulin-4-glucoside were detected in all species; however, they are way more abundant in *P. montanum* extract. Luteolin and its glucuronide derivatives shared a common fragment at *m/z* 285.0415. The glucuronide derivatives show additional fragments at 461.0743 and 637.1054, respectively, corresponding to [M-glucuronic acid] (loss of 176 Da) [101]. Nepetin showed a mass spectrum with a loss of 15 Da, characteristic of the methyl radical in methoxylated flavonoids [102].

Eleven flavonols are described among bioactive metabolites, mostly found in Asteraceae species. Six of them were quercetin derivatives, with quercetagetin-7-O-glucoside (ID− 271), quercetin-O-acetyl-hexoside (ID− 666) and quercetin-3-O-robinobioside (ID− 960), and unfully determined quercetin hexoside (ID− 751) in *Helichrysum* species, while patuletin-3-O-glucuronide (ID− 448) and 6-methoxyquercetin (ID− 993), also called patuletin, were found in species from the *Arnica* pair. The latter showed fragments at *m/z* 301.0362, 463.0897, 151.0044, and 178.9994 in negative ionization mode. Kramberger et al. [58] described a compound as a quercetin hexoside in *H. italicum* hydroethanolic extracts with the same fragments, without more precision. Among those, quercetagetin-7-O-glucoside (ID− 271) appeared as one of the major contributors to the antioxidant activity observed in the *Helichrysum* species, with one of the tallest peak areas in On-Line assays. Although described in plant extracts with antioxidant properties, the antioxidant potential of this compound has never been assessed to our knowledge. Its aglycone part, quercetagetin, is known to exhibit strong antioxidant activity, with EC_50_ values ranging from 1.27 to 10.5 µg/mL according to DPPH assays [103,104,105].

In addition to quercetin derivatives, luteolin-7-O-rutinoside (ID− 433), myricetin acetyl-hexoside (ID− 436), methoxy-myricetin-3-O-glucoside (ID− 468), isorhamnetin-3-O-glucoside (ID− 641), and 3,5-dihydroxy-6,7,8-trimethoxyflavone (ID− 1562) were pointed out as antioxidant compounds. Among those, myricetin acetyl-hexoside (ID− 436) emerged as one of the major contributors to the antioxidant activity of the *Helichrysum* extracts. While the isolated acetylated derivative antioxidant capacity has never been assessed, its aglycone part, myricetin, is known for its antioxidant properties, with DPPH EC_50_ values ranging from 1.3 to 9.0 µg/mL [106,107].

Two aromatic polyketides derived from phloroglucinols contributed to the radical scavenging activity of the Helichrysum pair, namely arenol (ID− 1638) and arzanol (ID− 1746). Arzanol has already been described as an efficient antioxidant agent, comparable to BHT, according to the autoxidation of linoleic acid assay [78]. Moreover, a nitrogen compound classified among ornithine alkaloids, namely N1,N5,N10,N14-tetra-trans-p-coumaroylspermine (ID− 1303), figures among the bioactive compounds. Its antioxidant activity has already been described, although it has been evaluated as moderate [108].

This approach of post-column derivatization with DPPH and ABTS reagents allows the gathering of chromatographic data, spectrometric data, and radical scavenging activity profile within a single experiment [109]. The major contributors to the antioxidant activity were phenylpropanoids derived from cinnamic acid: caffeoylquinic acid derivatives in the Asteraceae species, and rosmarinic acid in the Lamiaceae species. This way, the biologically active compounds can be identified efficiently, avoiding the time-consuming and often unproductive process of blindly purifying each compound for offline assays. While some authors suggest that ABTS post-column assay is better than DPPH, especially in terms of base-line stability [110,111,112], no difference between the two reagents was observed here. However, while the in vitro assays provide valuable insights into radical scavenging properties, further investigations are required. In particular, cell-based assays would be necessary to evaluate bioavailability and efficacy in biological systems, for industrial use in cosmetics or pharmaceutics. In the food industry, the incorporation of natural antioxidants as food ingredients could help prevent oxidative rancidity, thereby preserving the nutritional value, organoleptic properties, and shelf life of food products. Similarly, in cosmetics and pharmaceuticals, antioxidant compounds contribute to formulation stability, skin protection, and anti-inflammatory effects.

**Table 2:** Antioxidant compounds detected by UHPLC-DPPH/ABTS-HRMS/MS in negative mode ionization.

Peak Area (mAU)						Rt (min)	Formula	Annotation	CL	ID−	[M−H]^−^	[M−H]^−^ Fragments (Relative Intensities in %)	**Ref** **.**
AM	PM	HI	HS	SH	SM								
1954	-	3565	4634	-	-	1.65	C_16_H_18_O_9_	3-caffeoylquinic acid	L2a	75	353.0877 (−0.3)	191.0556 (100), 179.0346 (64), 135.0446 (64), 85.0290 (7)	[92]
32,896	62,763	59,859	74,503	-	-	3.58	C_16_H_18_O_9_	5-caffeoylquinic acid	L1	111	353.0879 (+0.3)	191.0571 (100), 353.0894 (73), 179.0360 (65), 135.0459 (27), 161.0252 (6), 173.0465 (4), 155.0361 (1)	[92]
4298	1863	1259	2011	2589	3148	3.70	C_9_H_8_O_4_	caffeic acid	L1	118	179.0349 (−0.5)	135.0454 (100), 89.0403 (12), 107.0505 (5), 117.0343 (4)	[113]
-	-	-	-	1929	2186	6.58	C_18_H_16_O_8_	rosmarinic acid derivative	L3	189	359.0772 (+3.1)	161.0241 (100), 174.9554 (63), 197.0452 (57), 135.0453 (50)	[114]
944	5088	-	-	-	-	6.89	C_25_H_24_O_12_	1,3-dicaffeoylquinic acid	L2b	208	515.1188 (−1.4)	191.0565 (100), 353.0878 (96), 179.0355 (86), 135.0457 (26), 515.1187 (15), 335.0780 (12), 161.0239 (9)	[93]
-	-	18,988	19,462	-	-	8.14	C_21_H_20_O_13_	quercetagetin-7-O-glucoside	L1	271	479.0829 (−0.4)	317.0311 (100), 479.0847 (27), 165.9914 (5), 139.0044 (3)	[115]
-	-	-	-	-	6796	9.29	C_27_H_32_O_15_	eriodictyol-7-rutinoside	L2a	354	595.1671 (+0.4)	287.0570 (100), 151.0045 (61), 595.1673 (49), 506.1708 (23), 135.0458 (17), 459.1150 (7)	[116]
-	2311	-	-	-	-	9.72	C_21_H_18_O_12_	luteolin-7-glucuronide	L2a	405	461.0727 (+0.3)	285.0417 (100), 461.0743 (11), 300.0283 (3)	[117]
-	-	26,450	27,237	-	-	9.90	C_23_H_22_O_14_	myricetin-acetylhexoside	L2a	436	521.0938 (+0.2)	317.0290 (100), 521.0952 (45), 329.1405 (10), 165.9915 (7), 463.0835 (6)	[118]
-	-	-	-	11,630	12,262	9.91	C_27_H_30_O_16_	luteolin-7-rutinoside	L2a	433	593.1513 (+0.2)	285.0412 (100), 593.1519 (99)	[119]
17,590	-	-	-	-	-	10.02	C_22_H_20_O_14_	patuletin-3-glucuronide	L3	448	507.0779 (−0.3)	331.0447 (100), 316.0210 (60), 507.0768 (19), 287.0187 (17), 270.0166 (9)	[120]
5062	4828	-	-	-	-	10.19	C_22_H_22_O_13_	methoxy-myricetin-3-O-hexoside	L2a	468	493.0988 (+0.1)	330.0392 (100), 493.0995 (91), 315.0157 (89), 287.0206 (25)	[121]
-	-	-	-	-	4209	10.63	C_27_H_32_O_14_	naringenin-7-rutinoside	L2a	533	579.1724 (+0.8)	271.0622 (100), 579.1726 (29), 151.0042 (13), 313.0730 (3)	[122]
111,632	103,103	102,694	102,494	-	-	10.82	C_25_H_24_O_12_	1,5-dicaffeoylquinic acid	L1	562	515.1197 (+0.4)	191.0571 (100), 353.0894 (73), 179.0360 (65), 135.0459 (27), 161.0252 (6)	[93]
14,299	-	-	-	-	-	11.38	C_22_H_22_O_12_	isorhamnetin-3-O-glucoside	L2a	641	477.1037 (−0.3)	477.1024 (100), 314.0420 (81), 299.0184 (58), 271.0238 (36), 243.0287 (17)	[123]
-	-	28,068	29,179	-	-	11.48	C_23_H_22_O_13_	quercetin-3-O-glucosyl-6′-acetate	L2a	666	505.0988 (+2.2)	301.0355 (100), 505.1006 (2)	[46]
-	-	-	-	70,991	70,405	11.63	C_18_H_16_O_8_	rosmarinic acid	L2a	685	359.0778 (+1.6)	161.0252 (100), 197.0464 (38), 135.0458 (31), 179.0358 (21), 72.9937 (21), 123.0458 (16)	[119]
-	-	15,354	17,140	-	-	11.79	C_25_H_24_O_12_	3,5-dicaffeoylquinic acid	L2a	707	515.1194 (−0.2)	353.0881 (100), 173.0455 (61), 179.0349 (49), 191.0562 (25), 135.0449 (16), 515.1205 (9)	[93]
1368	17376	-	-	-	-	11.91	C_22_H_22_O_11_	hispidulin-4-glucoside	L2a	735	461.1088 (−0.3)	461.1108 (100), 283.0261 (93), 297.0416 (15)	[124]
-	-	5353	5577	-	-	12.08	C_21_H_20_O_12_	quercetin-hexoside	L2b	751	463.0882 (+0)	301.0362 (100), 463.0897 (32), 151.0044 (24), 178.9994 (16)	[58]
-	-	11,015	11,382	-	-	12.41	C_28_H_26_O_15_	1,3-dicaffeoyl-4-methoxy-oxaloyl-quinic acid	L2a	798	601.1199 (+0)	395.0989 (100), 233.0671 (66), 353.0882 (44), 173.0459 (40), 191.0566 (28), 179.0353 (26), 439.0883 (9)	[59]
-	-	-	-	2335	2671	12.94	C_27_H_22_O_12_	lithospermic acid	L2a	861	537.1040 (+0.3)	135.0460 (100), 295.0620 (97), 161.0253 (79), 359.0782 (75), 179.0360 (35), 197.0466 (33)	[125]
-	-	5247	5667	-	-	13.81	C_30_H_26_O_14_	quercetin-3-O-robinobioside	L2a	960	609.1251 (+0.2)	300.0282 (100), 609.1265 (92), 463.0894 (46)	[126]
-	12,944	-	-	-	-	13.87	C_15_H_10_O_6_	luteolin	L1	965	285.0405 (+0.1)	285.0415 (100), 133.0301 (45), 151.0044 (6), 107.0144 (4)	[58]
9640	-	-	-	-	-	14.18	C_16_H_12_O_8_	patuletin	L2a	993	331.0457 (−0.7)	316.0231 (100), 165.9919 (39), 110.0018 (34), 331.0477 (24), 139.0039 (22), 181.0157 (10), 121.0308 (9)	[127]
-	-	-	-	2524	4667	14.21	C_27_H_22_O_12_	lithospermic acid derivative	L3	999	537.1047 (+1.6)	339.0518 (100), 357.0623 (60), 519.0936 (52), 283.0619 (20), 197.0462 (17), 295.0617 (15)	[128]
-	26204	-	-	-	-	14.39	C_16_H_12_O_7_	nepetin	L1	1016	315.0510 (−0.1)	300.0283 (100), 315.0516 (14), 136.9886 (10), 201.0201 (6), 133.0299 (5), 65.0039 (4)	[129]
-	-	-	-	1974	15905	14.62	C_26_H_22_O_10_	salvianolic acid A	L2a	1051	493.1140 (+0)	161.0246 (100), 135.0453 (75), 359.0777 (56), 197.0459 (29), 179.0352 (27), 295.0613 (13)	[130]
-	-	-	-	3699	3566	15.14	C_15_H_12_O_5_	naringenin	L1	1082	271.0612 (+0)	119.0505 (100), 151.0043 (68), 107.0143 (26), 271.0617 (25), 83.0143 (20), 65.0039 (19), 93.0351 (17)	[58]
-	2702	-	-	-	-	15.41	C_37_H_32_O_18_	1,4,5-tricaffeoyl-3-methoxy- oxaloylquinic acid	L2a	1108	763.1509 (−0.9)	395.0978 (100), 353.0874 (23), 233.0661 (18), 557.1305 (17), 515.1189 (16), 677.1512 (15), 763.1509 (12), 179.0346 (12), 601.1195 (11)	[59]
16,527	5714	-	-	-	-	19.06	C_46_H_50_N_4_O_8_	N1,N5,N10,N14-tetra-trans-*p*- coumaroylspermine	L2a	1303	785.3553 (−0.4)	785.3547 (100), 545.2404 (81), 665.2977 (55), 145.0296 (9)	[131]
-	-	9396	8821	-	-	23.54	C_18_H_16_O_7_	3,5-dihydroxy-6,7,8-trimethoxy-flavone	L2a	1562	343.0824 (+0.2)	313.0352 (100), 270.0179 (63), 328.0588 (34), 186.0321 (31), 285.0409 (21), 242.0218 (20), 298.0126 (16)	[132]
-	-	379	479	-	-	24.48	C_21_H_24_O_7_	arenol	L2a	1638	387.1451 (+0.4)	235.0982 (100), 247.0980 (88), 191.1080 (17), 139.0403 (15), 95.0504 (14)	[133]
-	-	1295	1230	-	-	25.67	C_22_H_26_O_7_	arzanol	L2a	1746	401.1606 (+0.1)	235.0972 (100), 247.0973 (91), 191.1074 (23), 109.0655 (20), 153.0553 (18), 205.0866 (11)	[58]

Peak Area according to UV Chromatogram (325 nm); Rt: retention time; CL: confidence level (with L1: confirmed structure (standard), L2a: library spectrum match, L2b: diagnostic evidence, L3: tentative candidate); ID−: MZmine ID in negative ionization mode; Ref.: references.

## 3. Materials and Methods

### 3.1. Plant Material

Three pairs of plant species were constituted (Table 3): the hereafter labeled “*Arnica* pair” with *Arnica montana* and *Pentanema montanum* (formerly known as *Inula montana* L.), the *Helichryum* pair with *Helichrysum italicum* and *Helichrysum stoechas*, and the *Satureja* pair with *Satureja hortensis* and *Satureja montana*. Voucher specimens were deposited in the Laboratory of Pharmacognosy of Aix-Marseille University (Marseille, France). The plant material was either supplied by an industrial partner from existing batches already in their possession and currently in use or, for species not commercially available, harvested in the wild in Provence. All plant materials were stored in their dried form under controlled conditions, under 25 °C and 60% relative humidity, ensuring the stability of key metabolites.

### 3.2. Chemical Supplies

Solvents of analytical grade (methanol, ethanol, acetic acid glacial, formic acid) and LC-MS grade (water, formic acid, and acetonitrile) were purchased from CarloErba (Italy). Folin-Ciocalteu, DPPH (2,2-DiPhenyl-1-PicrylHydrazyl), ABTS (2,2′-Azino-bis-(3-ethylBenzoThiazoline-6-Sulfonic acid) diammonium salt) reagents, boric acid, and oxalic acid were purchased from Sigma Aldrich, sodium carbonate and potassium peroxodisulfate from Fluka Biochemika, gallic acid and isoquercitrin from Extrasynthese.

### 3.3. Extraction and Sample Preparation

Each extraction was performed in triplicate, using 0.5 g of herbal drug and 20 mL of 70% ethanol (1:40 m/V), by ultrasound-assisted extraction for 15 min at 25 °C (PEX05 25 kHz, Reus, France). These extraction conditions were selected based on preliminary laboratory studies to ensure efficient and rapid extraction. The extracts were filtered using 15–45 µm polypropylene frits. For UHPLC-MS/MS analysis, 500 µL was diluted to 2.0 mL in the same solvent and filtered again through 0.2 µm PTFE filters (Restek, France).

Ethanol from the remaining liquid extracts was evaporated under vacuum with a Speedvac (Thermo Scientific, Waltham (MA), USA), and the residual aqueous phase was freeze-dried (Cryotec, France) and stored in air-tight containers in the dark at −20 °C. Extraction yields are presented in Table S2 of the Supplementary Material.

### 3.4. Chemical Composition Determination

#### 3.4.1. UHPLC-MS/MS Analysis

The UHPLC-MS/MS analysis was performed on a Thermo Scientific Dionex 3000 UHPLC paired to a Bruker Impact II Q-TOF high-resolution mass spectrometer, equipped with an electrospray ionization source (ESI).

Chromatographic separation was achieved at 43 °C with an Agilent Zorbax Eclipse Plus C18 column (2.1 × 100 mm, 1.8 µm), protected by a Phenomenex SecurityGuard ULTRA C18 pre-column (3 mm). A gradient mode was used with a 0.8 mL/min flow rate: (A) water and (B) acetonitrile, both acidified at 0.1% formic acid (*v*/*v*): isocratic at 5% B for 2 min, 5–30% B over 2–17 min, 30–70% B over 17–27 min, 70–100% B over 27–31 min, then isocratic at 100% B for 1 min (31–32 min), followed by a decrease to 5% B in 0.1 min (32–32.1 min), held at 5% B over 32.1–34 min for the column equilibration for the next analysis. The injection volume was 1 µL for all samples.

Mass spectrometry data were acquired in both positive and negative ionization modes across a mass range of 50 to 1500 *m/z*. For both ionization modes, the following parameters were applied: acquisition rate at 8 Hz; nebulizer gas (N_2_) pressure at 3.5 bar; dry gas flow (N_2_) at 12 L/min; drying temperature at 250 °C; end plate offset at 500 V. The capillary voltage was set at 3500 V for positive mode and 3000 V for negative mode.

Using a data-dependent acquisition (DDA) protocol, MS/MS fragmentation spectra were generated for the three most abundant precursor ions, using mixed collision energy 20–40 eV (stepping mode). A sodium formate/acetate solution forming clusters in the studied mass range was used as the calibrant and automatically injected at the beginning of each injection for internal mass calibration, ensuring a precision of *m*/*z* lower than 2 ppm in the mass range.

#### 3.4.2. Data Processing

Raw datasets obtained from the UHPLC-MS/MS analysis were first calibrated using Bruker DataAnalysis (5.0) and then converted into open format «.mzXMLx» using MSConvert from ProteoWizard [134]. Data conversion of Bruker data to mzXML format led to an error in the exact mass of the fragmented ions in MS1, making it difficult to pair them with the precursor ions observed in MS2. Therefore, a “precursor value corrector” [135] script was used after conversion to fix this issue, according to MZmine documentation. The script is freely available on GitHub [136].

Corrected “.mzXML” data were processed using MZmine software v3.9 [137], using a workflow inspired by the literature [138,139,140], with parameters described in Table S3 of Supplementary Materials. These parameters yielded a feature list of 2223 features in negative mode and 2178 features in positive mode, which were exported for Feature-Based molecular Networking in GNPS, and for MS^2^ spectra annotation with in silico tool SIRIUS.

#### 3.4.3. Molecular Networking with GNPS

Two molecular networks were created following the Feature-Based Molecular Networking (FBMN) workflow [49] on GNPS [50], using the default parameters for both ionization modes (Table S4 of Supplementary Materials). GNPS FBMN jobs can be publicly accessed at https://gnps.ucsd.edu/ProteoSAFe/status.jsp?task=aca51211c28f4d86becbe474d41cf293 (URL accessed on 27 February 2024) for negative and https://gnps.ucsd.edu/ProteoSAFe/status.jsp?task=96a1de9f0c6b4e6c981230384342c15f (URL accessed on 26 February 2024) for positive ionization mode, respectively. The resulting molecular networks were visualized using Cytoscape software 3.9.1 [141].

#### 3.4.4. Features Annotation

Features annotation was carried out by manual verification of the structure hypothesis obtained by different computational metabolomics tools, such as GNPS, SIRIUS, and timaR. The GNPS spectral libraries were consulted for feature annotation [50,142].

The SIRIUS software (v5.8.6) is designed for the structure elucidation of molecules from mass spectrometry data [143]. It accurately predicts molecular formulas and structures through the generation of fragmentation trees explaining experimental data.

The molecular fingerprints generated are compared to those contained in existing databases, such as COCONUT, GNPS, and custom databases.

Moreover, the CANOPUS tool [144] is used to predict compound classification, using the NPClassifier taxonomy system with 7 pathways, 70 superclasses, and 672 classes [43]. The TimaR (Taxonomically Informed Metabolite Annotation Repository) tool aims to generate a new ranking of structural hypotheses, depending on taxonomic data of the studied organisms [145].

### 3.5. Evaluation of Antioxidant Activity

#### 3.5.1. DPPH/ABTS Well-Plate Assays

##### Sample Preparation and Plate Layout

The dried hydro-ethanolic extracts were solubilized in methanol (MeOH) at 1 mg/mL and diluted at different concentrations ranging from 1 to 100 µg/mL, optimized to reach the EC_50_. Gallic acid, used as a positive control, was also solubilized in MeOH and diluted to obtain concentrations from 0.5 to 5 μg/mL for the DPPH assay and from 0.05 to 1 µg/mL for the ABTS assay.

The experiments were carried out in 96-well plates (Sterilin Ltd., Newport, UK), each filled according to the plan described in Figure S1. The first row is filled with methanol (MeOH, 250 µL) for the blank. The second row is filled with DPPH or ABTS solution (200 µL) and MeOH (50 µL), for the control, which represents the starting absorbance of DPPH or ABTS solution. The next six rows are divided into three groups of four columns each: (i) the first three filled with 200 µL of DPPH or ABTS solution and 50 µL of either gallic acid or the samples in decreasing concentrations, and (ii) the last one with 200 µL of MeOH and 50 µL of gallic acid or the samples in decreasing concentration. Each sample was tested in triplicate.

##### DPPH Assay

A DPPH methanolic solution at a concentration of 10^−4^ M is extemporaneously prepared by dissolving 3.0 mg of DPPH in 50 mL MeOH and stored at room temperature in the dark for 1 h before use. The well-plates were incubated at 25 °C in the spectrophotometer (EON BioTek, Providence (RI), USA) for 1 h, and absorbance was measured at 515 nm.

##### ABTS Assay

The ABTS assay was performed with freshly prepared methanolic ABTS solution at a concentration of 87.5 µM, extemporaneously prepared by diluting the stock solution of ABTS reagent in MeOH. The ABTS stock solution was prepared by dissolving 192.1 mg of ABTS reagent into 50 mL distillate water, and mixing it in a 1:1 ratio with an aqueous solution of potassium persulfate (K_2_S_2_O_8_), prepared by dissolving 66.2 mg of K_2_S_2_O_8_ into 50 mL distillate water.

The mixture is stored in a dark place at room temperature for 12–16 h before use. The well-plates were incubated at 25 °C in the spectrophotometer (EON BioTek, Providence (RI), USA) for 30 min and absorbance was measured at 734 nm.

##### EC_50_ Determination

The scavenging activity in % is calculated as [(*Abs_control_ − Abs_sample_)/Abs_control_*] × 100, with *Abs_control_* being the absorbance of DPPH or ABTS radical solution, and *Abs_sample_* being equal to *Abs_sample_ − Abs_sample blank_*. Blank absorbance is automatically subtracted from every other absorbance. Results are expressed as EC_50_, the concentration corresponding to a 50% reduction in absorbance.

#### 3.5.2. On-Line RP-UHPLC-DPPH/ABTS-MS/MS Assay

The post-column derivatization experimental setup, inspired by Breaud et al. [135] and Litewski et al. [109], consists of a UHPLC-based chromatographic separation with an adjustable flow splitter (Analytical, Flanders (NJ), USA) in two flows. One stream leads to a reaction coil with DPPH or ABTS solution, followed by a UV-Vis detector to assess analyte reactivity with the reagent by measuring the absorbance decline at the maximum absorption wavelength of each reagent. The second stream is routed through a nano-flowmeter (Bronkhorst, Netherlands) to HRMS/MS equipped with a nanoESI ionization source for structural characterization of radical-scavenging analytes. This setup is represented in Figure S4.

The UHPLC-DAD system (1290 Infinity, Agilent, Santa Clara (CA), USA) was equipped with an injector system (G4226A), a binary pump (G4220A), a thermostat (G1330B), and a DAD-UV detector (G4212A). Chromatographic separation was carried out with an Agilent Zorbax Eclipse Plus C18 column (2.1 × 100 mm, 1.8 µm), protected by a C18 pre-column Phenomenex SecurityGuard ULTRA (3 mm). Ultrapure water (A) and acetonitrile (B), both acidified with 0.1% (*v*/*v*) formic acid, were used as the mobile phase. All solvents used were of LC-MS grade. Separation was carried out at 43 °C, using a flow rate of 0.2 mL/min and an injection volume of 2 µL for every sample. The following gradient was applied: isocratic at 5% B for 2 min, 5–30% B over 2–17 min, 30–70% B over 17–27 min, 70–100% B over 27–31 min, then isocratic at 100% B for 1 min (31–32 min), followed by a decrease to 5% B in 0.1 min (32–32.1 min), held at 5% B over 32.1–35 min. DAD detector was configured between 190 and 600 nm with monitoring at 325 nm.

Using an adjustable flow splitter (Analytical, USA), the UHPLC effluent is split in a 1:50 ratio, and directed to (i) an HPLC-DAD system (1200 series, Agilent, USA) system equipped with a quaternary pump (G1311A) for the delivery of reagents across a reaction coil (peek tube 23 m × 0.18 mm) and a DAD UV-Vis detector (G1315B), and (ii) a Synapt G2-Si time-of-flight mass spectrometer (Waters, Wilford (AZ), USA). The HPLC quaternary pump delivered the reagents into the reaction coil at a flow rate of 0.2 mL/min, with the temperature of the reaction coil set at 60 °C. With these parameters, the reaction time between each sample and reagent solution is 1 min 6 s. The UV-Vis detection of the reaction solution was carried out at 515 nm for DPPH and 734 nm for ABTS. A fresh DPPH methanolic solution was prepared extemporaneously at a concentration of 2 × 10^−4^ M and kept at room temperature in the dark for at least 3 h before use. A fresh ABTS methanolic solution at a concentration of 0.21 mM was prepared extemporaneously by diluting the stock solution of ABTS reagent in MeOH. Mass spectrometry data were acquired in both positive and negative ionization modes across a mass range of 50 to 1200 *m/z,* with the following parameters: scan time of 0.25 s, capillary voltage of the nanosource set at 3000 V, source offset of 80, source temperature of 95 °C, nanoflow gas (N_2_) pressure at 1.5 bar. Leucine-enkephalin solution was used for lockspray calibration every 15 s.

Using a DDA protocol, MS/MS fragmentation spectra were generated for the three most abundant precursor ions, using trap collision energy 20–60 eV (stepping mode). A sodium formate solution was used for initial calibration of the spectrometer, ensuring a precision of *m*/*z* lower than 5 ppm in the mass range.

### 3.6. Total Phenolic and Flavonoid Contents

The total phenolic content (TPC) was evaluated according to the Folin–Ciocalteu colorimetric method, according to Breaud et al. [135].

The total flavonoid content (TFC) was evaluated using the oxalo-boric acid method, adapted from a monograph of the European Pharmacopoeia [146]. The dried hydro-ethanolic extracts were prepared in ethanol (EtOH) 50% (*v*/*v*) at different concentrations, adapted to reach optimal absorbances between 0.2 Au and 0.6 Au, from 0.2 mg/mL to 1 mg/mL. For each solution, 10 mL was mixed with 10 mL of oxalo-boric reagent, prepared by mixing 2.5 g of boric acid and 2.0 g of oxalic acid into 100 mL of anhydrous formic acid. The solution was then diluted in a 25 mL volumetric flask, completed with glacial acetic acid. After 30 min at room temperature, the absorbance of the solution was measured at 425 nm using a UV-Vis spectrophotometer (Genesys 10S, ThermoScientific, Loughborough, Leicestershire, UK). TFC was expressed as mg of isoquercitrin equivalent (IQEq) per 100 mg of dry extract.

### 3.7. Statistical Analysis

Statistical analyses were conducted using GraphPad Prism (8.0.2), applying ordinary one-way ANOVA with uncorrected Fisher’s LSD multiple comparisons.

## 4. Conclusions

This study investigated the phytochemical composition and antioxidant potential of three pairs of closely related medicinal plant species, selected on the basis of their common uses in phytotherapy, namely *Arnica montana* and *Pentanema montanum*, *Helichrysum stoechas* and *Helichrysum italicum*, *Satureja montana* and *Satureja hortensis*. The application of dereplication and molecular networking approaches allowed us to describe and compare the phytochemical composition of these species, including some understudied ones.

The antioxidant potential was assessed using DPPH and ABTS microplate assays, and the metabolites contributing to the bioactivity were identified precisely through post-column assays. Most of the compounds involved in the antioxidant activity are phenolic compounds, especially phenylpropanoids and flavonoids. The major contributors to the antioxidant activity are phenylpropanoids derived from cinnamic acid: caffeoylquinic acid derivatives in the Asteraceae species, especially 5-caffeoylquinic acid and 1,5-dicaffeoylquinic acid, and rosmarinic acid in the Lamiaceae species.

The results highlight the antioxidant properties of Provence species, which are either comparable to or even superior to those of their more widely known relatives. Therefore, the Provence species could be considered in pharmaceutical, nutraceutical, cosmetic, or food industries, and prevent overharvesting of over-exploited plants, such as *A. montana*. Promoting these species supports biodiversity conservation efforts while preserving local traditional knowledge and plant biodiversity.

## Figures and Tables

**Table 1:** Antioxidant activity (DPPH and ABTS EC_50s_), total phenolic content (TPC), total flavonoid content (TFC), and flavonoids proportion among phenolics (TFC/TPC).

Species	DPPH EC_50_ (µg/mL)	ABTS EC_50_ (µg/mL)	TPC (%)	TFC (%)	TFC/TPC (%)
*A. montana*	45.71 ± 3.05 ****	15.59± 2.51 ***	12.64 ± 3.13	5.88 ± 0.05 **	46.52
*P. montanum*	23.66 ± 0.75 ****	9.23 ± 0.70 ***	15.47 ± 0.52	7.96 ± 0.18 **	51.45
*H. italicum*	17.21 ± 2.38	5.53 ± 0.04	18.15 ± 2.59	12.93 ± 0.42 **	71.24
*H. stoechas*	17.58 ± 0.28	3.81 ± 0.75	21.44 ± 1.65	15.01 ± 1.32 **	70.01
*S. hortensis*	20.59 ± 0.39	5.88 ± 2.44	16.87 ± 0.50	4.62 ± 0.44	27.39
*S. montana*	19.66 ± 1.53	5.33 ± 1.15	17.13 ± 2.87	4.87 ± 0.51	28.43
Gallic acid	1.72 ± 0.25	0.35 ± 0.06	-	-	-

Data are expressed as the mean of triplicates ± standard deviation. Statistical differences within a pair according to one-way ANOVA followed by uncorrected Fisher’s LSD test (**: *p* < 0.05, ***: *p* < 0.02, ****: *p* < 0.0001).

**Table 3:** Plant material information.

Pair	*Arnica* Pair		*Helichrysum* Pair		*Satureja* Pair	
	Phytotherapy	Provence	Phytotherapy	Provence	Phytotherapy	Provence
Species	*Arnica* *montana* L.	*Pentanema* *montanum* (L.) D.Gut.Larr., Santos-Vicente, Anderb., E.Rico & M.M.Mart.Ort.	*Helichrysum italicum* (Roth) G. Don	*Helichrysum stoechas* (L.) Moench	*Satureja* *hortensis* L.	*Satureja* *montana* L.
Sample name	AM	PM	HI	HS	SH	SM
Plant part	flower heads	flower heads	flower heads	flower heads	leaves	leaves
Source	Père Blaize Supplier	wild (Provence)	“Moulin Bonaventure” Supplier	wild (Provence)	PMA28 Supplier	Cailleau Supplier
Voucher	AMFl.FR.1	IMFl.FR.20.1	HIFl.FR.21.1	HSFl.FR.18.1	SHF.FR.22.1	SMF.FR.19.1
Harvest	2022	2020	2021	2018	2022	2019

## Data Availability

The data presented in this study are openly available in a publicly-accessible repository. Raw UHPLC-MS/MS data are available at: ftp://massive.ucsd.edu/v07/MSV000096561/ (accessed on 10 December 2024) (temporary link: ftp://MSV000096561@massive.ucsd.edu; password: v8E93K0OM6w4ILOh) and treated data at: https://zenodo.org/records/14229843 (accessed on 10 December 2024). GNPS FBMN jobs can be publicly accessed at https://gnps.ucsd.edu/ProteoSAFe/status.jsp?task=aca51211c28f4d86becbe474d41cf293 (accessed on 28 November 2024) for negative and https://gnps.ucsd.edu/ProteoSAFe/status.jsp?task=96a1de9f0c6b4e6c981230384342c15f (accessed on 28 November 2024) for positive ionization mode, respectively.

## Supplementary Materials

The following supporting information can be downloaded at: https://www.mdpi.com/article/10.3390/molecules30092072/s1, Figure S1: Heatmaps showing (A) shikimates & phenylpropanoids repartition in superclasses and (B) flavonoids repartition in classes, in negative and positive ionization modes; Figure S2: Base Peak Chromatograms of each pair in negative (NEG) and positive (POS) ionization modes; Figure S3: Well-plate layout for DPPH assay; Figure S4: Experimental setup of the UHPLC-DPPH/ABTS-HRMS/MS On-Line assays; Table S1: UHPLC-HRMS/MS metabolite profiling; Table S2: Extraction yields; Table S3: MZmine parameters; Table S4: Feature-Based Molecular Networking (FBMN) parameters.
