# Supplementary material for: Can Provence Flora Offer Effective Alternatives to Widely Used Medicinal Plants? A Comparative Study of Antioxidant Activity and Chemical Composition Using Molecular Networking
Source: Molecules. 2025 May 7;30(9):2072. doi: 10.3390/molecules30092072 (PMC12073109; doi:10.3390/molecules30092072)
Supplement: Supplementary file 1 [file molecules-30-02072-s001.zip › molecules-3602276-supplementary.pdf]

Figure S1: Heatmaps showing (A) shikimates & phenylpropanoids repartition in superclasses and (B) flavonoids repartition in classes, in negative and positive ionization modes

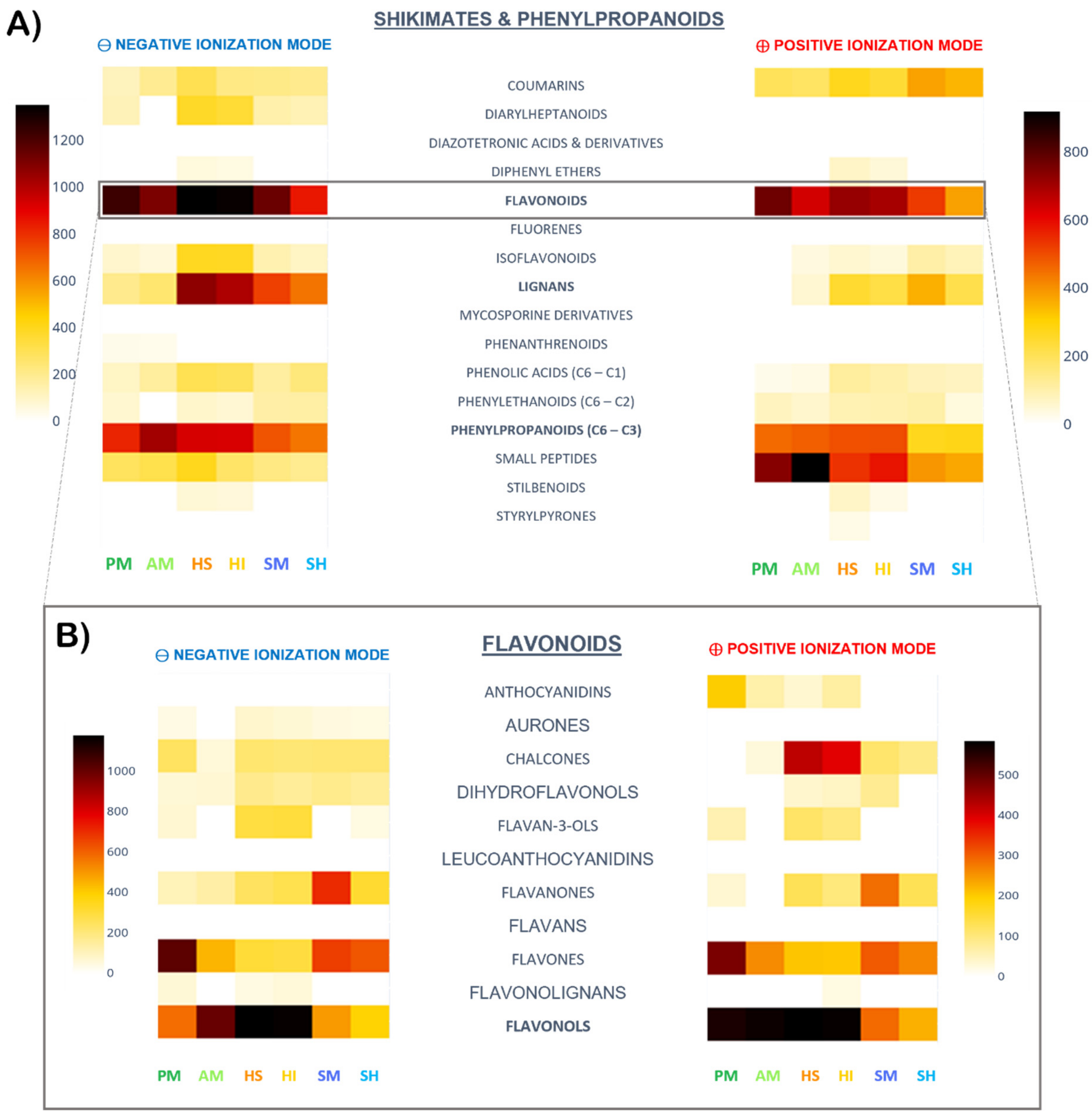

Figure S2: Base Peak Chromatograms of each pair in negative (NEG) and positive (POS) ionization modes

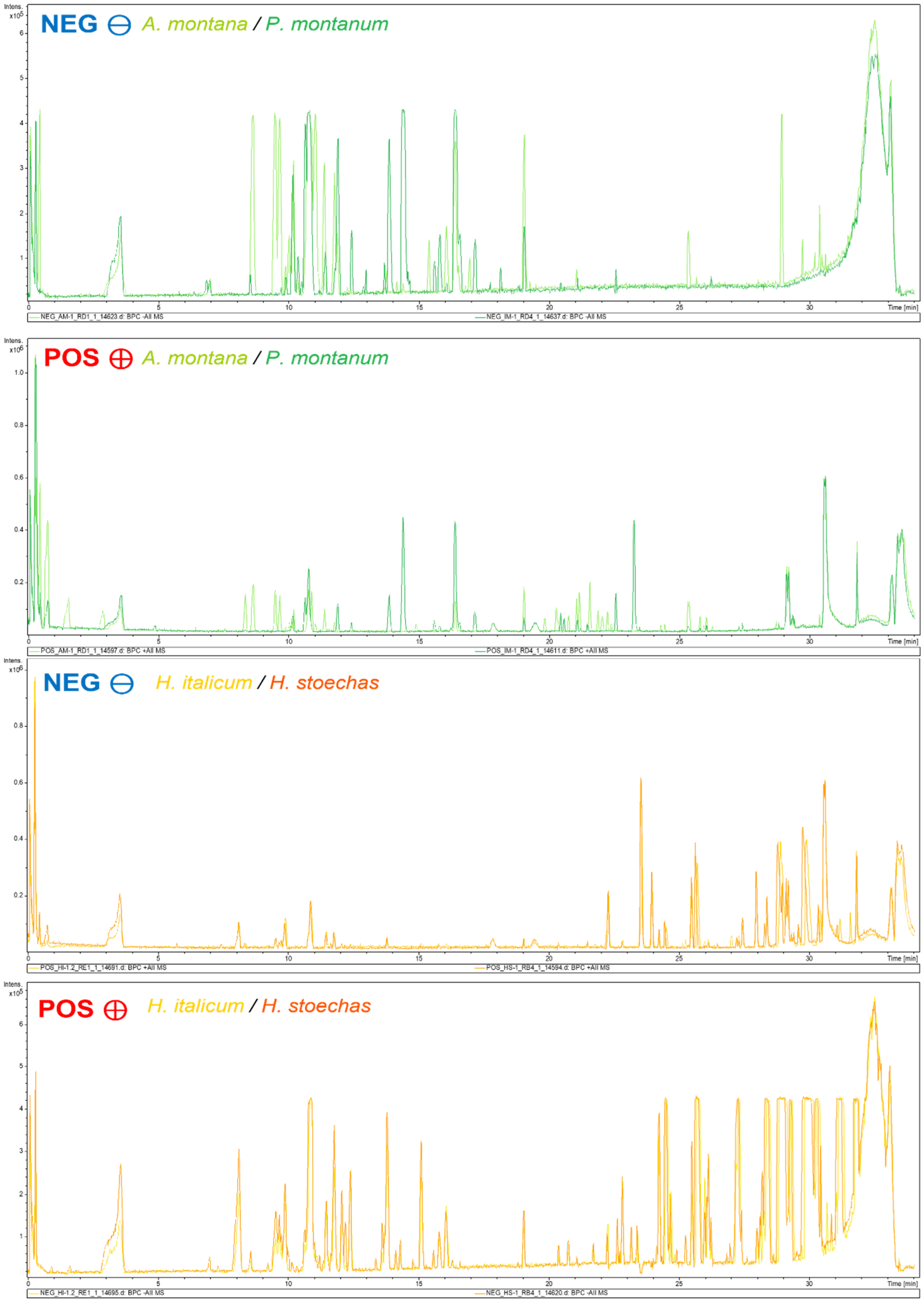

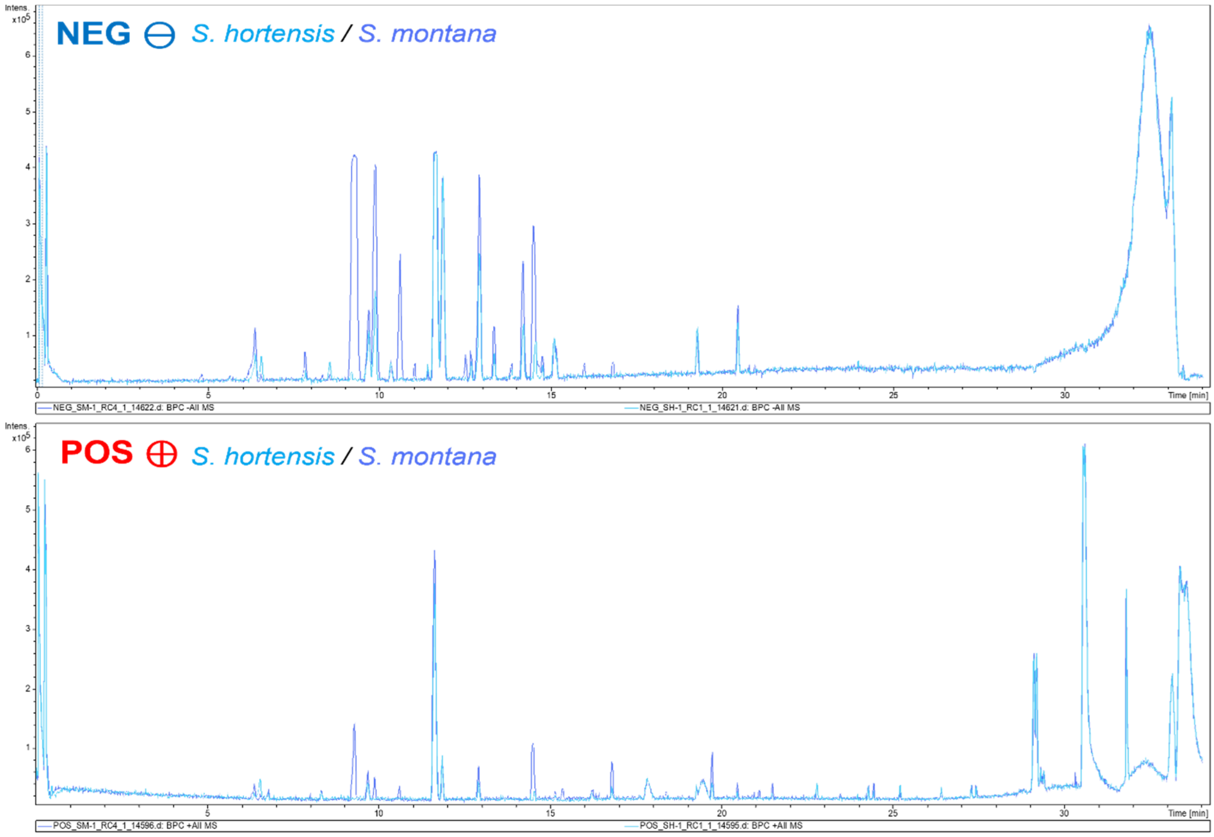

Figure S3: Well-plate layout for DPPH assay

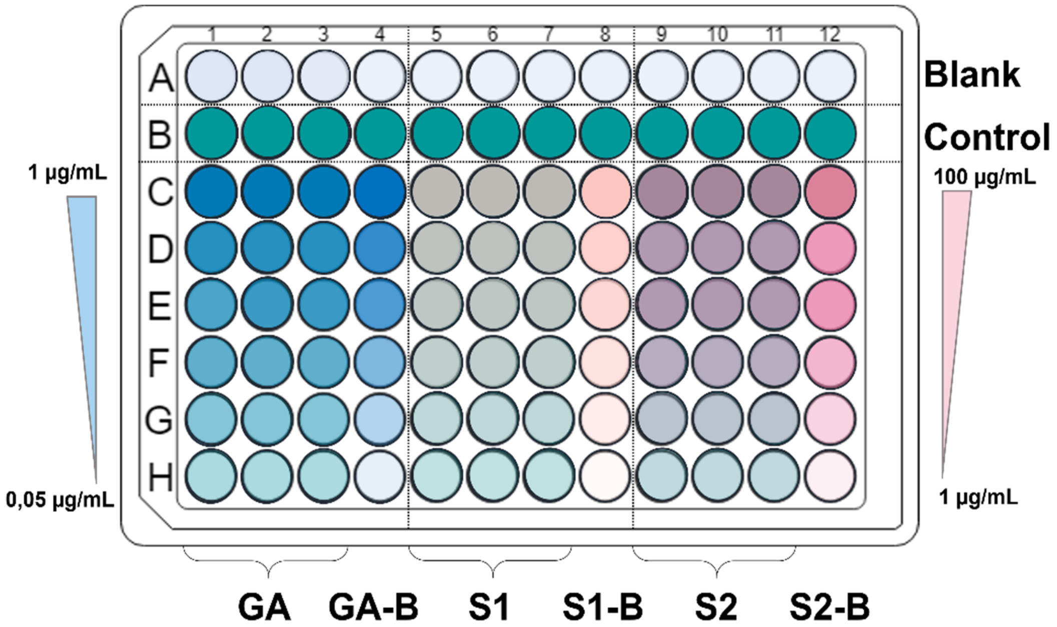

with: GA = Gallic acid, GA-B = Gallic acid blank, S1 = Sample 1, S1-B = Sample 1 Blank, S2 = Sample 2, S2-B = Sample 2 Blank

Figure S4: Experimental setup of the UHPLC-DPPH/ABTS-HRMS/MS On-Line assays

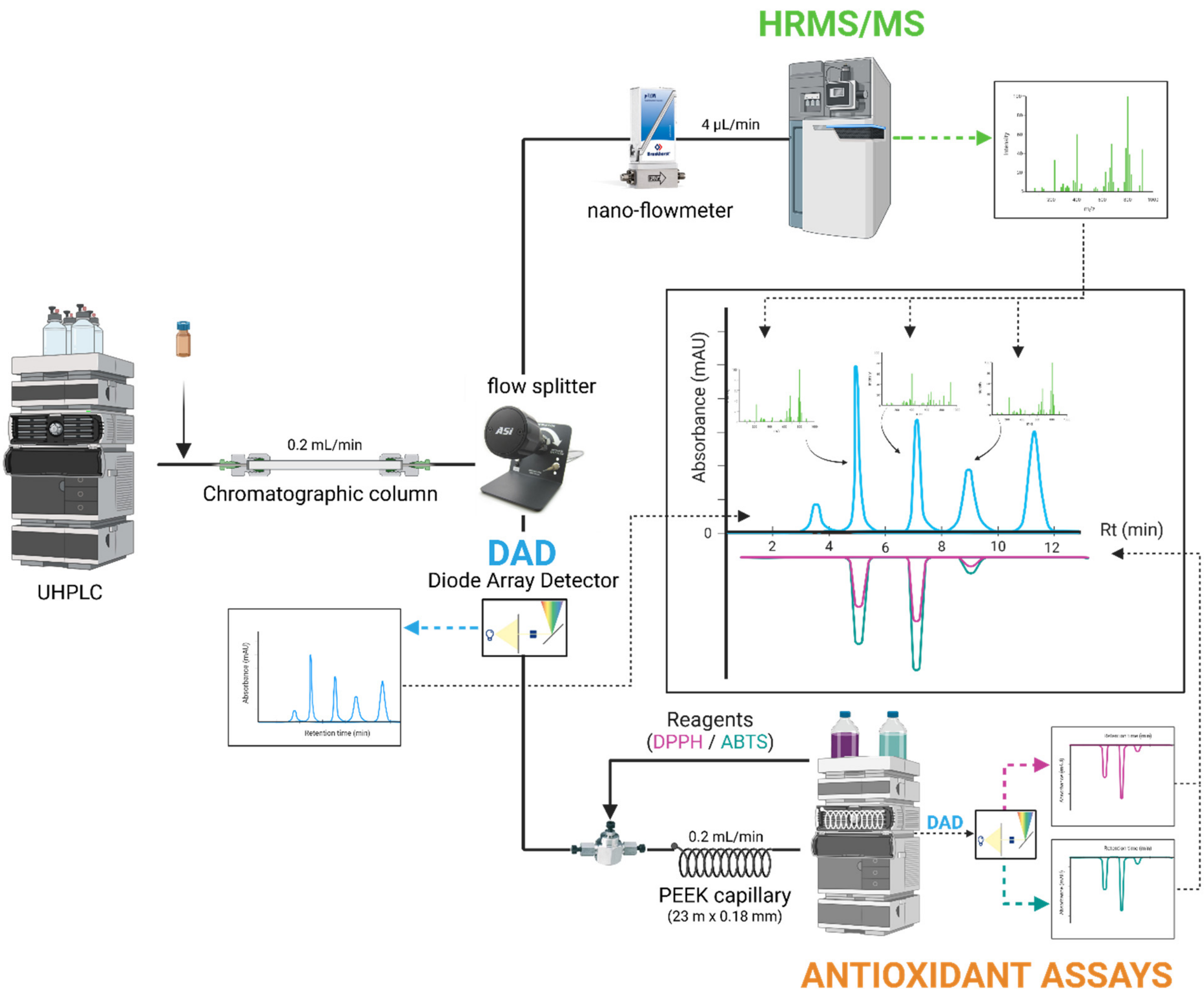

Table S4: UHPLC-HRMS/MS metabolite profiling

AM	PM	HI	HS	SH	SM	Rt (min)	Annotation	CL	Molecular Formula	ID(-)	(M-H]$^-$ (error in ppm)	[M-H]- fragments	ID(+)	(M+H]$^+$ (error in ppm)	[M+H]$^+$ fragments	Ref
				√		0.36	quinic acid	L1	$C_7H_{12}O_6$	8	191.0562 (+0.5)	85.0296 (100), 191.0561 (79), 93.0347 (33), 59.0141 (20), 127.0400 (17), 44.9987 (16), 71.0139 (13)	ND	ND	ND	[1]
√	√					0.42	2-butenedioic acid	L2a	$C_4H_4O_4$	31	115.0037 (+0.2)	73.0298 (100), 71.0137 (32), 59.9861 (28), 55.0191 (7)	ND	ND	ND	[2]
√	√	√	√	√	√	0.46	isoleucine	L2a	$C_6H_{13}NO_2$	ND	ND	ND	43	132.102 (+0.7)	86.0962 (100), 69.0695 (28), 57.0571 (3)	[3]
√	√	√	√		√	0.80	phenylalanine	L2a	$C_9H_{11}NO_2$	51	164.0715 (-1.2)	72.0090 (100), 103.0553 (68), 96.9616 (36), 147.0441 (34)	60	166.0863 (+0.3)	120.0807 (100), 103.0541 (44), 91.0540 (8), 107.0495 (5), 79.0538 (5)	[4]
√	√	√	√	√	√	0.95	protocatechuic acid-O-hexoside	L2a	$C_{13}H_{16}O_9$	56	315.0721 (-0.2)	108.0213 (100), 152.0109 (39), 315.0719 (29), 112.9861 (6)	ND	ND	ND	[5]
√	√	√	√	√	√	1.58	tryptophan	L2a	$C_{11}H_{12}N_2O_2$	72	203.0825 (-0.5)	116.0507 (100), 74.0247 (44), 142.0669 (11)	98	205.0972 (+0.2)	146.0598 (100), 188.0705 (58), 118.0650 (57), 144.0804 (30), 143.0726 (27), 115.0541 (24), 159.0914 (20), 132.0808 (19), 130.0648 (16)	[6]
√		√	√			1.65	3-caffeoylquinic acid	L2a	$C_{16}H_{18}O_9$	75	353.0877 (-0.3)	191.0556 (100), 179.0346 (64), 135.0446 (64), 85.0290 (7)	102	355.1022 (-0.4)	163.0389 (100), 135.0441 (13), 145.0288 (5), 355.0996 (4), 117.0333 (3)	[7]
√		√	√			2.31	dihydroxycoumarin-6-glucoside	L2a	$C_{15}H_{16}O_9$	94	339.0721 (-0.2)	177.0195 (100), 133.0286 (10), 105.0343 (6), 339.0734 (5)	ND	ND	ND	[8]
√						2.90	2,3,4,9-tetrahydro-1H-carboline-3-carboxylic acid	L2a	$C_{12}H_{12}N_2O_2$	101	215.0827 (+0.5)	116.0517 (100), 92.0508 (96), 72.0096 (88), 142.0653 (83), 86.0258 (82), 171.0933 (37), 134.3479 (35)	132	217.097 (-0.7)	144.0802 (100), 130.0648 (4), 74.0229 (3), 117.0694 (2), 103.0537 (2)	[9]
√		√	√		√	3.16	caffeic acid-3-glucoside	L2a	$C_{15}H_{18}O_9$	105	341.0878 (0)	135.0443 (100), 179.0344 (76), 203.0268 (6)	ND	ND	ND	[10]
√			√			3.54	esculetin	L2a	$C_9H_6O_4$	109	177.0192 (-0.7)	105.0344 (100), 177.0192 (95), 133.0291 (78), 89.0396 (51), 93.0352 (26), 149.0235 (25)	148	179.034 (+0.6)	123.0438 (100), 179.0339 (91), 133.0286 (47), 91.0542 (33), 119.0492 (30), 89.0379 (21), 105.0332 (21)	[11]

				√	√	3.58	5-caffeoylquinic acid	L1	$C_{16}H_{18}O_9$	111	353.0879 (+0.3)	191.0571 (100), 353.0894 (73), 179.0360 (65), 135.0459 (27), 161.0252 (6), 173.0465 (4)	151	355.1022 (-0.4)	163.0392 (100), 145.0279 (14), 135.0443 (9), 117.0341 (9)	[7]
√	√	√	√	√	√	3.70	caffeic acid	L1	$C_9H_8O_4$	118	179.0349 (-0.5)	135.0454 (100), 89.0403 (12), 107.0505 (5), 117.0343 (4)	159	181.0494 (-0.7)	163.0398 (100), 135.0453 (83), 89.0391 (67), 108.9593 (38), 145.0288 (38), 131.9750 (31), 126.9696 (27), 117.0347 (20)	[12]
			√			5.91	coumaric acid	L1	$C_9H_8O_3$	158	163.0399 (-1)	199.0500 (100), 93.0345 (10), 120.0537 (4)	ND	ND	ND	[5]
				√	√	6.21	thymoquinol-5-O-glucopyranoside	L3	$C_{16}H_{24}O_7$	173	327.145 (+0.2)	164.0842 (100), 149.0604 (68), 134.0366 (6), 174.9566 (6)	ND	ND	ND	[13]
√						6.38	roseoside	L2a	$C_{19}H_{30}O_8$	ND	ND	ND	244	387.2016 (+0.7)	207.1380 (100), 95.0853 (57), 123.0804 (50), 149.0973 (24), 189.1273 (24), 113.0583 (21), 163.1123 (16), 135.1178 (14)	[14]
	√		√	√	√	6.41	hydroxyjasmonic acid isomer	L2a	$C_{12}H_{18}O_4$	ND	ND	ND	249	227.1278 (+0.1)	149.0958 (100), 131.0851 (90), 191.1070 (52), 107.0852 (48), 91.0535 (46), 121.1012 (34)	[15]
	√	√	√	√	√	6.42	tuberonic acid glucoside	L2a	$C_{18}H_{28}O_9$	182	387.1659 (-0.4)	387.1647 (100), 59.0141 (46), 89.0240 (17), 207.1033 (17), 71.0141 (9)	ND	ND	ND	[16]
	√					6.44	4-O-caffeoylshikimic acid	L2a	$C_{16}H_{16}O_8$	183	335.0767 (-1.6)	135.0445 (100), 179.0344 (67), 161.0229 (15), 93.0340 (13)	253	337.092 (+0.6)	227.1271 (100), 209.1179 (70), 191.1065 (37), 85.0638 (23), 131.0853 (18)	[17]
				√	√	6.46	6-(3-benzoyloxy-2-hydroxypropoxy)-3,4,5-trihydroxyoxane-2-carboxylic acid	L2a	$C_{16}H_{20}O_{10}$	184	371.0982 (-0.5)	249.0613 (100), 121.0283 (70), 59.0146 (55), 371.0992 (47), 113.0238 (36), 164.0847 (33), 87.0081 (29)	ND	ND	ND	[18]
				√	√	6.59	rosmarinic acid derivative	L2b	$C_{18}H_{18}O_9$	189	359,0772 (0)	161.0241 (100), 174.9554 (63), 197.0452 (57), 135.0453 (50)	258	361.0916	163.0391 (100), 139.0391 (27), 181.0495 (23), 135.0438 (15), 361.0925 (5)	[17]
√	√					6.89	1,3-dicaffeoylquinic acid	L2a	$C_{25}H_{24}O_{12}$	208	515.1188 (-1.4)	191.0565 (100), 353.0878 (96), 179.0355 (86), 135.0457 (26), 515.1187 (15), 335.0780 (12), 161.0239 (9)	275	517.1345 (+0.9)	163.0388 (100), 145.0281 (4)	[19]
√	√	√	√			7.01	5-O-feruloylquinic acid	L2a	$C_{17}H_{20}O_9$	214	367.1033 (-0.4)	191.0563 (100), 93.0347 (51), 134.0376 (29), 87.0090 (18), 173.0454 (11), 111.0458 (9), 67.0198 (6)	282	369.1181 (+0.2)	177.0552 (100), 145.0290 (26), 117.0346 (6)	[20,21]
				√	√	7.46	apigenin-6,8-digalactoside	L2a	$C_{27}H_{30}O_{15}$	232	593.1509 (-0.5)	593.1527 (100), 353.0669 (52), 383.0785 (32), 473.1087 (26), 503.1204 (9)	304	595.1668 (+1.8)	325.0719 (100), 379.0823 (93), 409.0932 (61), 355.0822 (58), 337.0715 (57)	[22]
		√	√			7.47	scopoletin	L1	$C_{10}H_8O_4$	233	191.0348 (-1)	104.0268 (100), 176.0117 (71), 148.0162 (55)	303	193.0495 (-0.2)	193.0504 (100), 133.0294 (44), 178.0270 (32), 122.0366 (16), 94.0417 (14)	[23]

		√	√			7.66	myricetin-3-O-hexoside	L2a	$C_{21}H_{20}O_{13}$	245	479.083 (-0.2)	316.0211 (100), 479.0826 (98), 165.9904 (15)	321	481.098 (+0.7)	319.0447 (100), 85.0275 (6), 145.0495 (5)	[24]
				√	√	7.87	luteolin 7-O-beta-D-diglucuronide	L2a	$C_{27}H_{26}O_{18}$	254	637.1044 (-0.4)	285.0415 (100), 637.1054 (89), 351.0577 (68), 113.0252 (16), 193.0364 (11)	326	639.1205 (+2)	287.0554 (100), 639.1224 (5)	[25]
√		√	√			7.98	3,5,7-trihydroxy-2-[4-hydroxy-3-[3,4,5-trihydroxy-6-(hydroxymethyl)oxan-2-yl]oxyphenyl]-2,3-dihydrochromen-4-one	L2a	$C_{21}H_{22}O_{12}$	259	465.1037 (-0.3)	465.1033 (100), 125.0243 (77), 437.1087 (58), 285.0396 (29), 275.0547 (20), 152.0119 (19)	ND	ND	ND	[26]
	√	√	√			8.14	quercetagetin-7-glucoside	L1	$C_{21}H_{20}O_{13}$	271	479.0829 (-0.4)	317.0311 (100), 479.0847 (27), 165.9914 (5), 139.0044 (3)	344	481.0978 (+0.3)	319.0437 (100)	[27]
√	√	√	√	√	√	8.36	loliolide	L1	$C_{11}H_{16}O_3$	ND	ND	ND	362	197.1172 (-0.1)	179.1067 (100), 133.1010 (74), 107.0857 (67), 105.0698 (49), 161.0959 (48), 135.1168 (44), 91.0541 (33), 197.1168 (31)	[28]
	√	√	√	√	√	8.37	dihydroquercetin	L1	$C_{15}H_{12}O_7$	278	303.051 (-0.1)	125.0250 (100), 285.0419 (25), 153.0195 (16), 83.0141 (13)	357	305.0655 (-0.3)	153.0174 (100), 123.0429 (70), 231.0638 (45), 149.0234 (43), 259.0591 (37), 305.1554 (24)	[29]
				√	√	8.40	orientin	L2a	$C_{21}H_{20}O_{11}$	283	447.0933 (+0)	357.0614 (100), 327.0510 (99), 447.0946 (59), 174.9567 (36), 285.0379 (29), 297.0423 (27), 429.0806 (22)	ND	ND	ND	[30]
	√					8.46	methyl-chlorogenic acid	L2a	$C_{17}H_{20}O_9$	286	367.103 (-1.2)	135.0451 (100), 367.1042 (95), 179.0350 (89), 161.0252 (58), 233.0324 (12), 99.0094 (12)	ND	ND	ND	[31]
	√					8.56	2-O-glucopyranosyl-carboxy atracyligenin	L2a	$C_{26}H_{38}O_{11}$	290	525.2335 (-1.2)	481.2431 (100), 119.0345 (10), 89.0240 (10), 59.0138 (6)	ND	ND	ND	[5]
	√	√	√	√	√	8.59	quercetin alloside	L2a	$C_{21}H_{20}O_{12}$	299	463.0882 (+0)	301.0359 (100), 463.0896 (96), 136.9891 (4)	388	465.1033 (+1.2)	303.0509 (100)	[32]
√						8.65	8-decene-4,6-diynyl 2-O-glucopyranosyl glucopyranoside	L3	$C_{22}H_{32}O_{11}$	304	471.1872 (+0)	44.9991 (100), 425.1821 (30), 89.0245 (16), 381.1919 (10), 119.0350 (10), 113.0244 (7)	ND	ND	ND	[33]
√						8.66	dihydrohelenalin	L2b	$C_{15}H_{20}O_4$	ND	ND	ND	395	265.1437 (+1)	173.0970 (100), 145.1020 (94), 201.1283 (82), 131.0863 (42), 107.0865 (41), 105.0708 (40), 119.0863 (35)	[34]
	√					8.76	isorhamnetin-3,4-diglucoside	L2a	$C_{28}H_{32}O_{17}$	312	639.1562 (-0.7)	477.1023 (100), 639.1535 (89), 313.0324 (27), 112.9852 (23), 174.9549 (15), 304.9160 (15)	ND	ND	ND	[35]

					√	8.94	Flavone base + 3O, O-HexA-HexA	L2a	$C_{27}H_{26}O_{17}$	326	621.1101 (+0.6)	351.0561 (100), 113.0227 (21), 621.1140 (20), 269.0437 (18)	ND	ND	ND	[36]
	√	√	√			9.25	quercetin-glucuronide isomer	L3	$C_{21}H_{18}O_{13}$	347	477.0673 (-0.3)	301.0350 (100), 477.0671 (7), 113.0254 (4), 151.0033 (3)	435	479.0827 (+1.4)	303.0493 (100), 479.0856 (3)	[37]
				√	√	9.27	myricetin-3-rhamnoside	L1	$C_{21}H_{20}O_{12}$	351	463.0883 (+0.2)	316.0217 (100), 463.0855 (65), 271.0241 (23), 287.0181 (11)	433	465.1031 (+0.7)	319.0462 (100), 129.0553 (25), 85.0287 (24), 71.0499 (4)	[38]
				√	√	9.29	eriodictyol-7-rutinoside	L2a	$C_{27}H_{32}O_{15}$	354	595.1671 (+0.4)	287.0570 (100), 151.0045 (61), 595.1673 (49), 506.1708 (23), 135.0458 (17), 459.1150 (7)	452	597.1824 (+1.7)	289.0714 (100), 85.0285 (20), 129.0551 (19)	[39,40]
√						9.49	quercetin-3-glucuronide	L2a	$C_{21}H_{18}O_{13}$	374	477.0674 (-0.1)	301.0357 (100), 477.0671 (14), 151.0041 (13), 178.9989 (7)	465	479.0826 (+1.2)	303.0504 (100), 113.0237 (4), 85.0282 (4), 159.0291 (4), 131.0342 (3)	[41]
				√	√	9.55	5,7-dihydroxy-2-(4-hydroxyphenyl)-6-[3,4,5-trihydroxy-6-(hydroxymethyl)oxan-2-yl]-4H-chromen-4-one	L2a	$C_{21}H_{20}O_{10}$	388	431.0984 (+0.1)	311.0557 (100), 431.0991 (54), 283.0631 (53), 341.0672 (27), 323.0614 (16), 295.0606 (13), 158.9826 (12)	ND	ND	ND	[42]
	√	√	√			9.58	quercetin-3-O-glucoside	L1	$C_{21}H_{20}O_{12}$	391	463.0883 (+0.2)	301.0363 (100), 463.0897 (3)	470	465.103 (+0.5)	303.0489 (100)	[43]
√	√	√	√			9.66	isoquercitrin	L1	$C_{21}H_{20}O_{12}$	397	463.0881 (-0.2)	287.0553 (100), 463.0887 (3), 303.0504 (3)	474	465.103 (+0.5)	303.0507 (100), 85.0285 (7), 145.0500 (5), 127.0395 (4), 97.0286 (3)	[44]
	√			√	√	9.72	luteolin-7-glucuronide	L2a	$C_{21}H_{18}O_{12}$	405	461.0727 (+0.3)	285.0417 (100), 461.0743 (11), 300.0283 (3)	487	463.0877 (+1.3)	287.0558 (100), 463.0887 (3)	[45]
	√					9.77	isorhamnetin-3,7-diglucoside	L2a	$C_{28}H_{32}O_{17}$	417	639.1565 (-0.3)	477.1028 (100), 315.0487 (58), 639.1617 (24)	ND	ND	ND	[46]
√	√	√	√			9.82	dicaffeoylquinic acid glucoside	L2a	$C_{31}H_{34}O_{17}$	421	677.1724 (+1.8)	515.1415 (100), 323.0779 (69), 191.0557 (47), 677.1682 (20), 179.0338 (9), 161.0229 (8)	ND	ND	ND	[5]
√	√	√	√	√	√	9.87	luteolin-7-glucoside	L1	$C_{21}H_{20}O_{11}$	425	447.0933 (+0)	284.0335 (100), 447.0947 (91), 136.9890 (6)	502	449.1082 (+0.8)	287.0561 (100)	[47,48]
				√	√	9.90	luteolin-7-rutinoside	L2a	$C_{27}H_{30}O_{15}$	433	593.1513 (+0.2)	285.0412 (100), 593.1519 (99)	514	595.1669 (+1.9)	287.0560 (100), 449.1097 (16), 450.1134 (4)	[10,49]
		√	√			9.90	myricetin-acetylhexoside	L2a	$C_{23}H_{22}O_{14}$	436	521.0938 (+0.2)	317.0290 (100), 521.0952 (45), 329.1405 (10), 165.9915 (7), 463.0835 (6)	ND	ND	ND	[50]
		√	√			9.91	myricetin-malonyl hexoside	L2a	$C_{24}H_{22}O_{16}$	438	565.0835 (0)	317.0297 (100), 521.0932 (68)	516	567.0988 (+1.3)	319.0445 (100), 567.0991 (5)	[5]

		√	√			10.01	caffeoyl derivative	L3	$C_{34}H_{36}O_{19}$	445	747.1783 (+0.7)	747.1770 (100), 423.0909 (22), 585.1286 (22), 459.1281 (17), 323.0748 (13), 379.1025 (11)	526	749.1933 (+1.3)	163.0398 (100), 263.0562 (25), 325.0924 (11), 181.0502 (7)	[5]
√	√					10.02	patuletin-3-glucuronide	L2a	$C_{22}H_{20}O_{14}$	448	507.0779 (-0.3)	331.0447 (100), 316.0210 (60), 507.0768 (19), 287.0187 (17), 270.0166 (9)	528	509.0931 (+1)	333.0613 (100), 318.0379 (14), 113.0236 (5), 85.0281 (4)	[51]
√		√	√			10.05	quercetin-3-arabinoside	L1	$C_{20}H_{18}O_{11}$	455	433.0776 (-0.1)	300.0281 (100), 433.0789 (38), 271.0254 (30), 255.0298 (13), 243.0313 (8)	ND	ND	ND	[52]
√						10.11	3,14-dihydroxycostunolide-3-O-glycopyranoside	L3	$C_{21}H_{30}O_9$	459	425.1812 (-1.2)	119.0352 (100), 89.0248 (98), 101.0243 (82), 59.0146 (53), 381.1954 (50), 71.0140 (49), 113.0248 (49), 425.1796 (35), 245.1182 (29), 219.1394 (26), 85.0301 (21), 204.1164 (19)	ND	ND	ND	[53]
√	√	√	√			10.19	methoxy-myricetin-3-O-hexoside	L2a	$C_{22}H_{22}O_{13}$	468	493.0988 (+0.1)	330.0392 (100), 493.0995 (91), 315.0157 (89), 287.0206 (25)	546	495.1137 (+0.8)	333.0613 (100), 318.0380 (16), 85.0285 (7), 145.0497 (4)	[54]
				√	√	10.32	[2-[[3,4-dihydroxy-4-(hydroxymethyl)oxolan-2-yl]oxymethyl]-4-[4,5-dihydroxy-6-methyl-3-[3,4,5-trihydroxyoxan-2-yl]oxyoxan-2-yl]oxy-6-[2-(3,4-dihydroxyphenyl)ethoxy]-5-hydroxyoxan-3-yl]-3-(3,4-dihydroxyphenyl)prop-2-enoate	L2a	$C_{34}H_{44}O_{19}$	486	755.2406 (+0.3)	755.2409 (100), 593.2088 (14), 161.0254 (12)	ND	ND	ND	[55]
√	√	√	√	√	√	10.36	azelaic acid	L2a	$C_9H_{16}O_4$	496	287.0563 (+0.7)	125.0980 (100), 97.0659 (22), 169.0884 (16), 187.0981 (6)	564	189.1122 (+0.3)	97.1018 (100), 125.0964 (53), 83.0859 (28), 107.0867 (22)	[53,56]
		√	√			10.36	6-hydroxyquercetin	L1	$C_{15}H_{10}O_8$	489	187.0976 (+0.1)	317.0319 (100), 139.0040 (23), 111.0096 (18), 166.9987 (16), 194.9931 (6)	563	189.1122 (+0.3)	319.0447 (100), 137.0236 (8), 273.0392 (7), 245.0429 (7), 181.0109 (7)	[57]
	√		√	√	√	10.36	dihydrokaempferol	L2a	$C_{15}H_{12}O_6$	491	287.0563 (+0.7)	125.0246 (100), 259.0618 (41), 177.0560 (20), 83.0136 (17), 243.0672 (14), 287.0561 (11), 107.0143 (10)	ND	ND	ND	[58]
√	√					10.38	3,4-dicaffeoylquinic acid	L2a	$C_{25}H_{24}O_{12}$	498	515.1192 (-0.6)	353.0894 (100), 173.0463 (83), 179.0357 (71), 203.0358 (28), 191.0569 (27), 135.0455 (24), 255.0669 (11), 299.0572 (10), 161.0245 (9), 515.1198 (7)	568	517.1345 (+0.9)	163.0383 (100), 135.0433 (3), 145.0282 (3), 117.0327 (2)	[59]
	√	√	√			10.38	quercetin 3-O-malonylglucoside	L2a	$C_{24}H_{22}O_{15}$	499	549.089 (+0.7)	505.0977 (89), 301.0345 (81)	583	551.1043 (+2.1)	303.0489 (100), 551.0997 (1)	[60]

				√	√	10.40	salviaflaside	L2a	$C_{24}H_{26}O_{13}$	501	521.13 (-0.1)	161.0238 (100), 323.0757 (78), 359.0758 (62), 179.0338 (36), 197.0451 (29)	ND	ND	ND	[61]
	√					10.51	isorhamnetin-3-O-rutinoside	L2a	$C_{28}H_{32}O_{16}$	515	623.1612 (-0.9)	623.1619 (100), 315.0497 (69), 300.0256 (25), 300.0256 (16), 112.9860 (9)	ND	ND	ND	[62]
	√					10.52	isorhamnetin-3-O-glucuronide	L2a	$C_{22}H_{20}O_{13}$	519	491.0832 (+0.2)	315.0517 (100), 300.0286 (81), 491.0847 (13), 323.0780 (3)	589	493.0979 (+0.5)	317.0662 (100), 302.0426 (20), 493.0984 (3)	[25]
				√	√	10.59	verbascoside	L2a	$C_{29}H_{36}O_{15}$	525	623.1984 (+0.4)	271.0622 (100), 579.1727 (64)	ND	ND	ND	[63]
√	√	√	√	√		10.61	nepetin-7-glucoside	L1	$C_{22}H_{22}O_{12}$	527	477.1036 (-0.5)	477.1053 (100), 299.0210 (78), 315.0519 (39)	609	479.1187 (+0.6)	317.0651 (100), 302.0414 (22)	[64]
				√	√	10.63	naringenin-7-rutinoside	L2a	$C_{27}H_{32}O_{14}$	533	579.1724 (+0.8)	271.0622 (100), 579.1726 (29), 151.0042 (13), 313.0730 (3)	600	581.1872 (+1.2)	273.0757 (100), 129.0546 (28), 85.0282 (22), 147.0652 (11)	[65]
√	√	√	√	√		10.82	1,5-dicaffeoylquinic acid	L1	$C_{25}H_{24}O_{12}$	562	515.1197 (+0.4)	191.0571 (100), 353.0894 (73), 179.0360 (65), 135.0459 (27), 161.0252 (6)	616	517.1346 (+1.1)	163.0381 (100), 145.0277 (6), 135.0432 (6)	[19]
√		√	√			10.92	kaempferol-3-O-glucuronide	L2a	$C_{21}H_{18}O_{12}$	580	461.0724 (-0.3)	285.0394 (100), 229.0498 (10), 257.0448 (8), 113.0238 (7), 85.0288 (4)	626	463.0876 (+1.1)	287.0538 (100), 85.0275 (5), 113.0227 (4)	[66]
	√					10.93	tricaffeoyl hexaric acid	L2a	$C_{33}H_{28}O_{17}$	583	695.1254 (+0)	209.0313 (100), 371.0631 (65), 533.0954 (33), 534.0991 (11), 695.1268 (10), 191.0206 (9)	ND	ND	ND	[5]
√		√	√			11.01	kaempferol-3-O-glucoside	L1	$C_{21}H_{20}O_{11}$	588	447.0932 (-0.2)	447.0921 (100), 284.0313 (85), 255.0286 (57), 227.0337 (40)	630	449.1084 (+1.3)	287.0559 (100), 85.0286 (7)	[67,68]
√		√	√			11.05	1,5-dicaffeoyl-3-methoxyoxaloylquinic acid	L2a	$C_{28}H_{26}O_{15}$	598	601.1197 (-0.3)	233.0660 (100), 395.0974 (36), 191.0554 (8), 439.0868 (7), 179.0343 (6), 59.0139 (6)	635	603.1354 (+1.6)	163.0397 (100), 145.0289 (3)	[5]
				√	√	11.06	apigenin-7-rhamnoglucoside	L2a	$C_{27}H_{30}O_{14}$	603	577.1564 (+0.2)	269.0450 (100), 577.1562 (19)	634	579.1725 (+2.9)	271.0610 (100), 433.1149 (12), 85.0285 (4), 129.0553 (3)	[69]
√						11.09	quercetin-4'-glucoside	L1	$C_{21}H_{20}O_{12}$	606	463.0872 (-2.2)	301.0365 (100), 151.0041 (19), 179.0000 (12)	ND	ND	ND	[70]
√						11.14	kaempferol-3-galactoside	L2a	$C_{21}H_{20}O_{11}$	613	447.0925 (-1.8)	447.0938 (100), 284.0332 (82), 255.0305 (50), 227.0355 (39)	ND	ND	ND	[71]
	√	√	√	√	√	11.16	apigenin-7-O-glucuronide	L2a	$C_{21}H_{18}O_{11}$	617	445.0776 (-0.1)	269.0438 (100), 285.0397 (45), 113.0247 (9), 174.9542 (7), 85.0291 (6)	653	447.0926 (+0.9)	271.0592 (100), 287.0544 (18), 447.0902 (2)	[72]
		√	√			11.22	kaempferol-3-O-arabinoside	L2a	$C_{20}H_{18}O_{10}$	625	417.083 (+0.7)	417.0831 (100), 284.0321 (87), 255.0308 (67), 227.0359 (63)	659	419.0978 (+1.3)	287.0539 (100), 73.0277 (8), 115.0390 (2)	[73]
√	√	√	√	√	√	11.22	apigenin-7-O-glucoside	L1	$C_{21}H_{20}O_{10}$	626	431.0985 (+0.3)	268.0388 (100), 431.1002 (90)	663	433.1134 (+1.1)	271.0607 (100)	[74]

√		√	√			11.27	eriodictyol-7-glucoside	L2a	$C_{21}H_{22}O_{11}$	633	449.1086 (-0.7)	287.0553 (100), 151.0037 (99), 135.0454 (51), 449.1077 (42)	ND	ND	ND	[75]
√						11.36	7-methoxy-quercetin-3-O-glucuronide isomer	L2a	$C_{22}H_{20}O_{13}$	639	491.0825 (-1.3)	315.0502 (100), 300.0263 (41), 271.0241 (10), 113.0240 (7), 255.0287 (5)	677	493.0985 (+1.7)	317.0667 (100), 85.0284 (4), 302.0434 (4), 131.0339 (4), 113.0242 (4)	[76]
√	√	√	√			11.38	isorhamnetin-3-O-glucoside	L2a	$C_{22}H_{22}O_{12}$	641	477.1037 (-0.3)	477.1024 (100), 314.0420 (81), 299.0184 (58), 271.0238 (36), 243.0287 (17)	679	479.1189 (+1)	317.0644 (100), 302.0411 (14), 85.0276 (6)	[64]
		√	√			11.38	luteolin-glucoside isomer	L2a	$C_{21}H_{20}O_{11}$	640	447.0934 (+0.3)	284.0331 (100), 447.0939 (91), 151.0047 (12)	ND	ND	ND	[77]
		√	√			11.41	caffeic acid hexoside derivative	L2a	$C_{27}H_{36}O_{13}$	647	567.2081 (-0.4)	341.1403 (100), 326.1166 (63), 89.0249 (42), 119.0353 (33), 179.0574 (20)	ND	ND	ND	[5]
√						11.42	7-methoxy-quercetin-3-O-glucuronide isomer	L2a	$C_{22}H_{20}O_{13}$	651	491.0825 (-1.3)	315.0515 (100), 300.0281 (38), 113.0254 (13), 271.0253 (7), 85.0302 (6)	694	493.0987 (+2.1)	317.0667 (100), 85.0284 (4), 302.0434 (4), 131.0339 (4), 113.0242 (4)	[76]
		√	√			11.48	quercetin-3-O-glucosyl-6′-acetate	L2a	$C_{23}H_{22}O_{13}$	666	ND	301.0355 (100), 505.1006 (2)	ND	551.104 (+1.5)	ND	[78]
		√	√			11.48	flavonol base + 4O, O-malonylhex	L2a	$C_{24}H_{22}O_{15}$	ND	505.0988 (+0.1)	ND	705	ND	303.0499 (100), 551.1057 (2), 127.0389 (1)	[79]
	√			√	√	11.49	2-[4-[3-(4-hydroxy-3,5-dimethoxyphenyl)-1,3,3a,4,6,6a-hexahydrofuro[3,4-c]furan-6-yl]-2,6-dimethoxyphenoxy]-6-(hydroxymethyl)oxane-3,4,5-triol	L2a	$C_{27}H_{32}O_{14}$	673	579.2083 (+62.8)	417.1546 (100), 181.0498 (39), 402.1302 (18)	ND	ND	ND	[80]
		√	√			11.57	quercetin malonylhexoside isomer	L2a	$C_{24}H_{22}O_{15}$	678	549.0885 (-0.2)	505.0990 (100), 301.0359 (86), 151.0052 (15)	ND	ND	ND	[5,81]
	√			√	√	11.58	diosmetin-7-O-neohesperidoside	L2a	$C_{28}H_{32}O_{15}$	679	607.1665 (-0.6)	299.0554 (100), 607.1612 (12), 284.0325 (8)	ND	ND	ND	[81]
√	√					11.61	5-hydroxy-2-(4-hydroxy-3-methoxyphenyl)-7-[3,4,5-trihydroxy-6-(hydroxymethyl)(2H-3,4,5,6-tetrahydropyran-2-yloxy)]chromen-4-one	L2a	$C_{22}H_{22}O_{11}$	683	461.1085 (-0.9)	461.1442 (100), 161.0235 (59), 133.0299 (15), 281.0661 (14)		463.1228 (-1.5)	301.0715 (100), 286.0475 (16)	[82]
				√	√	11.63	rosmarinic acid	L1	$C_{18}H_{16}O_8$	685	359.0778 (+1.6)	161.0252 (100), 197.0464 (38), 135.0458 (31), 179.0358 (21), 72.9937 (21), 123.0458 (16)	729	361.0921 (+0.8)	163.0396 (100), 135.0447 (18), 139.0396 (9), 145.0290 (8), 181.0503 (6)	[10,83]
		√	√			11.67	kaempferol acetylglycoside	L2a	$C_{23}H_{22}O_{12}$	692	489.1039 (+0.1)	285.0405 (100), 489.1052 (6), 267.0319 (1), 185.0611 (1)	ND	535.109 (+1.4)	ND	[5]

		√	√			11.67	flavone base + 4O, O-MalonylHex	L3	$C_{24}H_{22}O_{14}$	ND	ND	ND	735	ND	287.0540 (100), 535.1075 (2)	[84]
				√	√	11.72	hesperidin	L1	$C_{28}H_{34}O_{15}$	698	609.1825 (+0)	301.0722 (100), 609.1825 (16)	738	611.1982 (+1.9)	303.0873 (100), 129.0553 (23), 85.0285 (18), 147.0657 (7), 153.0187 (7)	[85]
√		√	√	√	√	11.73	7-hydroxycoumarin	L2a	$C_9H_6O_3$	701	161.0244 (-0.1)	133.0295 (100), 105.0347 (12)	743	163.039 (+0.2)	89.0388 (100), 135.0447 (87), 117.0342 (56), 163.0398 (34), 145.0292 (21), 107.0497 (14), 79.0545 (9)	[86]
√	√	√	√			11.79	3,5-dicaffeoylquinic acid	L2a	$C_{25}H_{24}O_{12}$	707	515.1194 (-0.2)	353.0881 (100), 173.0455 (61), 179.0349 (49), 191.0562 (25), 135.0449 (16), 515.1205 (9)	751	517.1345 (+0.9)	163.0395 (100), 145.0292 (4), 135.0447 (4)	[19]
		√	√			11.82	myricetin-3-O-hexosyl(1-2)deoxyhexoside	L2a	$C_{27}H_{30}O_{17}$	713	625.1197 (-34.1)	625.1205 (100), 316.0232 (98), 479.0843 (41)	756	627.1353 (-32.3)	147.0440 (100), 319.0454 (16), 309.0977 (7), 165.0543 (6), 291.0862 (5)	[87]
√						11.84	syringetin-3-O-glucoside	L2a	$C_{23}H_{24}O_{13}$	717	507.1142 (-0.4)	507.1139 (100), 329.0311 (89), 301.0363 (51), 344.0547 (47)	759	509.1294 (+0.8)	347.0772 (100), 332.0545 (13), 85.0285 (7), 145.0495 (4)	[88]
√				√		11.88	kaempferol 3-alpha-L-arabinopyranoside	L2a	$C_{20}H_{18}O_{10}$	724	607.167 (+0.3)	417.0810 (100), 284.0315 (90), 256.0330 (6)	ND	ND	ND	[89]
				√	√	11.88	diosmetin-7-rutinoside	L1	$C_{28}H_{32}O_{15}$	720	417.0826 (-0.3)	299.0566 (100), 607.1676 (18), 284.0330 (18)	762		301.0717 (100), 463.1258 (16), 609.1850 (3), 85.0288 (3)	[90]
√		√	√			11.89	3,5-dicaffeoyl-4-methoxyaloylquinic acid	L2a	$C_{28}H_{26}O_{15}$	727	601.1197 (-0.3)	233.0668 (100), 173.0457 (33), 395.0975 (31)	769	603.1355 (+1.7)	163.0392 (100)	[5]
		√	√			11.90	luteolin-7-(6''-malonylglucoside)	L2a	$C_{24}H_{22}O_{14}$	729	533.0936 (-0.1)	284.0327 (100), 489.1025 (78)	771	535.1089 (+1.2)	287.0548 (100), 535.113 (2), 163.0378 (1)	[91]
√	√	√	√			11.91	hispidulin-4-glucoside	L2a	$C_{22}H_{22}O_{11}$	735	461.1088 (-0.3)	461.1108 (100), 283.0261 (99), 297.0416 (15)	774	463.1239 (+0.9)	301.0703 (100), 286.0466 (25)	[92]
	√				√	11.93	hispidulin-7-glucuronide	L2a	$C_{22}H_{20}O_{12}$	745	475.0883 (+0.2)	284.0329 (100), 299.0566 (83), 113.0249 (11), 59.0142 (7)	ND	ND	ND	[93]
	√					12.00	tricaffeoyl hexaric acid isomer	L2a	$C_{33}H_{28}O_{17}$	743	695.1254 (+0)	209.0308 (100), 371.0644 (97), 533.0949 (49), 695.1291 (16)	ND	ND	ND	[78]
	√	√	√	√	√	12.02	luteolin 4'-O-glucoside	L2a	$C_{21}H_{20}O_{11}$	746	447.0933 (+0)	285.0391 (100), 447.0902 (26), 174.9550 (9)	784	449.1086 (+1.7)	287.0540 (100), 449.1088 (11), 317.0654 (7)	[94]
√		√	√			12.08	quercetin-hexoside	L2b	$C_{21}H_{20}O_{12}$	751	463.0882 (+0)	301.0362 (100), 463.0897 (32), 151.0044 (24), 178.9994 (16)	786	465.1034 (+1.4)	303.0492 (100), 85.0276 (9)	[5,95]
√		√	√			12.27	3,5-dicaffeoyl-1,4-dimethoxyoxaloyl quinic acid	L2a	$C_{31}H_{28}O_{18}$	773	687.1201 (-0.3)	275.0761 (100), 233.0661 (22), 437.1081 (13), 395.0972 (8), 215.0551 (7), 179.0339 (6)	ND	ND	ND	[96]

		√	√			12.30	6-hydroxyapigenin	L2a	$C_{15}H_{10}O_6$	782	285.0405 (+0.1)	285.0410 (100), 117.0345 (42), 139.0032 (8)	805	287.0551 (+0.3)	287.0538 (100), 119.0484 (16), 123.0068 (15), 141.0173 (10)	[97]
	√					12.39	3,4,5-trihydroxy-6-[5-hydroxy-2-(4-hydroxy-3-methoxyphenyl)-3-methoxy-4-oxochromen-7-yl]oxyoxane-2-carboxylic acid	L2a	$C_{23}H_{22}O_{13}$	790	505.0982 (-1.1)	329.0671 (100), 314.0451 (99), 299.0217 (22), 174.9576 (12), 304.9125 (11), 113.0247 (10)	ND	ND	ND	[98]
√		√	√			12.41	1,3-dicaffeoyl-4-methoxyoxaloylquinic acid	L2a	$C_{28}H_{26}O_{15}$	798	601.1199 (+0)	395.0989 (100), 233.0671 (66), 353.0882 (44), 173.0459 (40), 191.0566 (28), 179.0353 (26), 439.0883 (9)	813	603.1355 (+1.7)	163.0397 (100)	[96]
	√					12.44	jaceoside	L2a	$C_{23}H_{24}O_{12}$	804	491.1192 (-0.6)	491.1212 (100), 313.0366 (38), 476.0973 (14), 328.0587 (9)	820	493.1345 (+0.9)	331.0802 (100), 316.0566 (20)	[99]
					√	12.53	salvianolic acid B derivative	L2a	$C_{36}H_{30}O_{16}$	815	717.1464 (+0.4)	357.0622 (100), 519.0941 (38), 717.1484 (8), 331.0831 (7), 339.0515 (7)	ND	ND	ND	[100]
	√					12.64	dehydrocostus lactone	L2a	$C_{15}H_{18}O_2$	ND	ND	ND	837	231.1378 (-0.7)	143.0857 (100), 105.0707 (91), 91.0551 (86), 131.0855 (68), 157.1023 (67), 185.1333 (64)	[101]
	√			√	√	12.7	medioresinol-O-hexoside	L2a	$C_{27}H_{34}O_{12}$	836	549.1977 (-0.1)	549.1976 (100), 387.1669 (99), 161.0254 (71), 207.1040 (15), 357.0627 (12)	845	551.213 (+1.3)	163.0379 (100), 325.0895 (12), 181.0486 (6), 307.0799 (2)	[102]
		√		√	√	12.87	eriodictyol	L2a	$C_{15}H_{12}O_6$	845	287.0561 (0)	135.0455 (100), 151.0042 (38), 107.0144 (10), 65.0036 (6), 83.0140 (5)	852	289.0707 (+0.1)	153.0189 (100), 163.0401 (66), 289.0720 (58), 135.0448 (19), 117.0338 (7)	[5,103]
√	√	√	√			12.89	3-feruloyl-5-caffeoylquinic acid	L2a	$C_{26}H_{26}O_{12}$	853	529.1351 (-0.1)	191.0565 (100), 367.1037 (71), 173.0458 (13)	851	531.1504 (+1.3)	177.0540 (100), 163.0382 (63), 145.0278 (15), 117.0328 (2)	[5]
				√	√	12.94	lithospermic acid A	L2a	$C_{27}H_{22}O_{12}$	861	537.104 (+0.3)	135.0460 (100), 295.0620 (97), 161.0253 (79), 359.0782 (75), 179.0360 (35), 197.0466 (33)	861	539.1195 (+2)	323.0559 (100), 163.0398 (47), 297.0765 (37), 181.0519 (19), 139.0404 (16)	[104]
		√	√			13.05	quercetin 3-O-(6''-acetyl-glucoside)	L2b	$C_{23}H_{22}O_{13}$	878	505.0988 (+0.1)	301.0358 (100), 505.0944 (3)	ND	ND	ND	[78]
				√	√	13.40	salvianolic acid B	L2a	$C_{36}H_{30}O_{16}$	917	717.1456 (-0.7)	519.0943 (100), 321.0415 (94), 339.0521 (44), 295.0621 (15), 717.1447 (5)	ND	ND	ND	[105]
	√					13.46	spinacetin	L2a	$C_{17}H_{14}O_8$	922	345.0615 (-0.3)	330.0363 (100), 315.0129 (84), 287.0173 (26), 164.9824 (14), 345.0616 (12), 153.0190 (9)	903	347.0759 (-0.7)	347.0737 (100), 258.0513 (52), 286.0461 (46), 209.1164 (35), 163.1100 (31), 314.0415 (17), 332.0527 (12)	[106,107]
√		√	√			13.62	4-feruloyl-5-caffeoylquinic acid	L2a	$C_{26}H_{26}O_{12}$	933	529.1347 (-0.9)	173.0449 (100), 367.1014 (60), 193.0492 (11)	909	531.1514 (+3.2)	177.0546 (100), 163.0394 (14), 145.0288 (11), 287.0503 (1)	[5]

√	√	√	√	√	√	13.74	quercetin	L1	$C_{15}H_{10}O_7$	952	301.0353 (-0.3)	151.0042 (100), 301.0356 (64), 178.9995 (41), 121.0298 (38), 107.0141 (30), 65.0037 (19), 83.0141 (14)	921	303.05 (+0.2)	303.0505 (100), 153.0188 (18), 229.0505 (12), 137.0241 (9), 201.0549 (5), 257.0458 (4), 173.0622 (4)	[108]
		√	√			13.81	quercetin-3-O-robinobioside	L2a	$C_{30}H_{26}O_{14}$	960	609.1251 (+0.2)	300.0282 (100), 609.1265 (92), 463.0894 (46)	933	611.1404 (+1.4)	147.0433 (100), 303.0492 (25), 309.0961 (14), 165.0540 (8)	[109]
√	√	√	√	√	√	13.87	luteolin	L1	$C_{15}H_{10}O_6$	965	285.0405 (+0.1)	285.0415 (100), 133.0301 (45), 151.0044 (6), 107.0144 (4)	937	287.055 (-0.1)	287.0547 (100), 153.0177 (15), 135.0437 (5)	[110]
					√	13.99	2-(3,4-dihydroxyphenyl)-5,7-dihydroxy-6,8-dimethoxychromen-4-one	L2a	$C_{17}H_{14}O_8$	977	345.0617 (+0.3)	330.0407 (100), 315.0155 (56), 215.0362 (46), 133.0303 (37), 345.0589 (30)	ND	ND	ND	[111]
		√	√			14.15	rutin	L2a	$C_{30}H_{26}O_{14}$	992	609.125 (+0)	300.0282 (100), 609.1262 (76), 463.0892 (38), 271.0253 (5)	952	611.1404 (+1.4)	147.0443 (100), 303.0496 (34), 119.0494 (4)	[109]
√	√					14.18	patuletin	L2a	$C_{16}H_{12}O_8$	993	331.0457 (-0.7)	316.0231 (100), 165.9919 (39), 110.0018 (34), 331.0477 (24), 139.0039 (22), 181.0157 (10), 121.0308 (9)	955	333.0604 (-0.3)	99.5316 (100), 195.1228 (53), 173.0790 (52), 88.0230 (51), 333.0616 (46)	[112]
				√	√	14.21	salvianolic acid B derivative	L3	$C_{27}H_{22}O_{12}$	999	537.1047 (+1.6)	339.0518 (100), 357.0623 (60), 519.0936 (52), 283.0619 (20), 197.0462 (17), 295.0617 (15)	ND	ND	ND	[113]
√	√					14.39	nepetin	L1	$C_{16}H_{12}O_7$	1016	315.051 (-0.1)	300.0283 (100), 315.0516 (14), 136.9886 (10), 201.0201 (6), 65.0039 (4)	970	317.0656 (+0.1)	317.0656 (100), 302.0421 (86), 168.0049 (31), 186.0157 (18)	[114]
√	√					14.52	carboxyatractyloside III	L2a	$C_{31}H_{46}O_{12}$	1032	609.2916 (-0.1)	565.3006 (100), 301.1806 (27), 463.2350 (26), 135.0468 (14), 101.0251 (14), 481.2437 (13)	ND	ND	ND	[115]
				√	√	14.60	scutellarein-4-methyl ether	L2a	$C_{16}H_{12}O_6$	1046	299.0561 (0)	284.0321 (100), 299.0575 (30), 136.9865 (16), 65.0026 (15)	990	301.0711 (+1.4)	301.0715 (100), 286.0472 (43), 186.0165 (29), 177.0556 (12), 89.0598 (11), 133.0863 (11), 168.0059 (11), 140.0094 (5)	[116]
	√					14.6	tetracaffeoylhexaric acid	L2a	$C_{42}H_{34}O_{20}$	1043	857.156 (-1.2)	371.0600 (100), 209.0291 (33), 857.1550 (29), 533.0913 (23), 695.1227 (11), 191.0189 (6)	ND	ND	ND	[117]
				√	√	14.62	salvianolic acid A	L2a	$C_{26}H_{22}O_{10}$	1051	493.114 (0)	161.0246 (100), 135.0453 (75), 359.0777 (56), 197.0459 (29), 179.0352 (27), 295.0613 (13)	ND	ND	ND	[113]
		√	√			14.67	6-hydroxykaempferol 7-O-(6″-caffeoylglucopyranoside)	L2a	$C_{30}H_{26}O_{15}$	1055	625.1197 (-0.3)	301.0368 (100), 625.1189 (11), 323.0779 (10), 161.0247 (8)	1004	627.1355 (+1.7)	303.0504 (100), 163.0390 (25), 325.0924 (13), 181.0499 (3)	[118]

				√	√	14.78	sagecoumarin	L2b	$C_{27}H_{20}O_{12}$	1062	535.088 (-0.4)	177.0201 (100), 161.0250 (39), 535.0888 (21), 359.0772 (20), 135.0457 (14), 107.0466 (11)	ND	ND	ND	[119]
√						14.90	acetyl-dihydrohelenalin	L2a	$C_{17}H_{22}O_5$	ND	ND	ND	1010	307.1541 (+0.3)	308.2235 (100), 247.1333 (33), 290.2135 (32), 173.0972 (25), 201.1287 (25), 136.0764 (22), 122.0605 (21), 145.1026 (21), 179.1320 (18), 309.2249 (17), 229.1244 (17), 262.2169 (14), 131.0861 (13), 173.1331 (11)	[34]
		√	√			15.12	tiliroside	L1	$C_{30}H_{26}O_{13}$	1080	593.1301 (+0.1)	593.1312 (100), 284.0334 (78), 255.0305 (6)	1026	595.1455 (+1.5)	147.0443 (100), 287.0553 (20), 309.0968 (8), 165.0549 (6)	[120]
√		√	√	√	√	15.14	naringenin	L1	$C_{15}H_{12}O_5$	1082	271.0612 (+0)	119.0505 (100), 151.0043 (68), 107.0143 (26), 271.0617 (25), 83.0143 (20), 65.0039 (19), 93.0351 (17)	1021	273.0757 (-0.2)	153.0174 (100), 273.0750 (51), 147.0426 (43), 119.0492 (23), 274.0774 (12)	[121]
				√	√	15.18	salvianolic acid A isomer	L3	$C_{26}H_{22}O_{10}$	1089	493.1141 (+0.2)	135.0459 (100), 161.0254 (89), 295.0621 (86), 359.0788 (40), 197.0467 (39), 179.0355 (34)	ND	ND	ND	[113]
√						15.41	1,4,5-tricaffeoyl-3-methoxyoxaloylquinic acid	L2a	$C_{37}H_{32}O_{18}$	1108	763.1509 (-0.9)	395.0978 (100), 353.0874 (23), 233.0661 (18), 557.1305 (17), 515.1189 (16), 677.1512 (15), 763.1509 (12), 179.0346 (12), 601.1195 (11)	ND	ND	ND	[122]
√	√	√	√			15.44	3,4,5-tricaffeoylquinic acid	L2a	$C_{34}H_{30}O_{15}$	1111	677.1509 (-0.4)	353.0871 (100), 515.1191 (73), 179.0344 (42), 173.0448 (39), 497.1084 (33), 677.1510 (29), 203.0340 (11)	ND	ND	ND	[123]
		√	√			15.50	methoxy-quercetin-7-O-hexosyl-deoxyhexoside isomer	L2a	$C_{31}H_{28}O_{14}$	1113	623.1404 (-0.4)	623.1408 (100), 315.0516 (83), 299.0194 (14)	1055	625.1566 (+2.3)	147.0434 (100), 317.0656 (15), 309.0961 (11), 165.0535 (6), 291.0854 (4), 119.0487 (3)	[124]
	√	√	√			15.58	methoxy-quercetin-7-O-hexosyl-deoxyhexoside isomer	L2a	$C_{31}H_{28}O_{14}$	1124	623.1404 (-0.4)	623.1408 (100), 315.0516 (83), 299.0194 (14)	ND	625.1566 (+2.3)	ND	[124]
	√					15.62	3,6-dimethoxyapigenin	L2a	$C_{17}H_{14}O_7$	1125	329.0665 (-0.5)	314.0445 (100), 299.0212 (96), 271.0261 (34), 215.0365 (23), 164.9838 (23)	1060	331.0815 (+0.8)	331.0805 (100), 242.0565 (46), 270.0515 (33), 316.0566 (10), 298.0463 (10), 121.0276 (6)	[125]

√						15.66	acetyl-helenalin	L2a	$C_{17}H_{20}O_5$	ND	ND	ND	1065	305.1384 (+0.2)	199.1123 (100), 227.1074 (78), 245.1190 (72), 135.0807 (49), 173.0965 (37), 184.0901 (34), 109.0649 (32), 107.0852 (28), 129.0702 (27), 145.1009 (25), 143.0852 (24)	[34]
√	√	√	√	√	√	15.81	apigenin	L1	$C_{15}H_{10}O_5$	1137	269.0456 (+0.2)	269.0464 (100), 117.0352 (56), 151.0039 (11), 107.0146 (6)	1073	271.0602 (+0.4)	271.0593 (100), 153.0176 (17), 119.0486 (7), 171.0288 (3)	[126]
				√	√	15.99	4,6-dihydroxy-2-[(4-hydroxy-3,5-dimethoxyphenyl)methylidene]-1-benzofuran-3-one	L2a	$C_{17}H_{14}O_7$	1143	329.067 (+1)	299.0208 (100), 314.0443 (75), 178.9996 (31), 271.0259 (26), 117.0345 (18), 329.0668 (16)	1078	331.0813 (+0.2)	331.0821 (100), 301.0347 (48), 298.0484 (25), 226.0630 (12), 170.0718 (10)	[127]
√	√	√	√	√	√	16.07	kaempferol	L1	$C_{15}H_{10}O_6$	1149	285.0405 (+0.1)	285.0408 (100), 93.0350 (3), 117.0348 (3), 185.0613 (2)	1081	287.0551 (+0.3)	287.0555 (100), 153.0187 (15), 121.0295 (6)	[128]
	√					16.34	chrysoplenol C	L2a	$C_{18}H_{16}O_8$	1161	359.077 (-0.7)	344.0538 (100), 329.0305 (65), 286.0116 (25), 230.0217 (18), 258.0157 (13)	1094	361.0921 (+0.8)	361.0920 (100), 272.0676 (51), 300.0626 (35), 346.0667 (14), 331.0446 (12), 328.0568 (11), 151.0387 (4)	[129]
√	√	√	√			16.46	hispidulin	L1	$C_{16}H_{12}O_6$	1173	299.0562 (+0.3)	284.0335 (100), 299.0568 (26), 256.0393 (17), 227.0356 (10), 151.0037 (8)	1101	301.0711 (+1.4)	301.0716 (100), 286.0479 (91), 168.0060 (39), 186.0169 (23), 121.0291 (5)	[130]
	√					16.51	2,3,4-tris[[-3-(3,4-dihydroxyphenyl)prop-2-enoyl]oxy]-5-(2-methylpropanoyloxy)hexanedioic acid	L2a	$C_{37}H_{34}O_{18}$	1178	765.1671 (-0.2)	279.0729 (100), 441.1034 (58), 603.1349 (40), 765.1692 (15), 191.0197 (14)	ND	ND	ND	[131]
√	√	√				16.53	3-methoxyquercetin	L1	$C_{16}H_{12}O_7$	1179	315.051 (-0.1)	243.0307 (100), 300.0277 (90), 271.0261 (67), 165.9909 (28), 315.0521 (27), 110.0017 (20)	1108	317.0658 (+0.7)	317.0659 (100), 302.0421 (57), 274.0489 (12), 121.0291 (10)	[132]
√	√	√	√			16.81	4-methoxyquercetin	L1	$C_{16}H_{12}O_7$	1200	315.051 (-0.1)	300.0278 (100), 315.0514 (61), 112.9865 (24), 151.0040 (12), 107.0143 (11), 271.0288 (9)	1133	317.0653 (-0.9)	317.0668 (100), 153.0185 (26), 302.0402 (15), 229.0471 (10), 285.0398 (9), 217.0486 (8)	[133]
				√	√	16.82	jaceidin	L2a	$C_{18}H_{16}O_8$	1201	359.0773 (+0.2)	344.0547 (100), 329.0308 (84), 359.0769 (27), 314.0067 (21), 178.9990 (12), 286.0112 (12), 301.0364 (12), 214.0275 (9)	1134	361.092 (+0.6)	361.0931 (100), 331.0460 (44), 328.0586 (19), 346.0692 (11), 256.0739 (9), 213.0400 (5), 149.0603 (4)	[134]
√	√	√	√	√	√	16.96	tricoumaroylspermidine	L2a	$C_{34}H_{37}N_3O_6$	1209	582.2607 (-0.4)	582.2592 (100), 462.2038 (67), 119.0500 (66), 342.1457 (61), 145.0294 (25), 463.2062 (24), 316.1663 (18), 174.9559 (11)	1142	584.2762 (+1.2)	147.0445 (100), 204.1025 (94), 438.2391 (73), 292.2030 (56), 420.2307 (28), 275.1755 (27), 584.2761 (15)	[135]

√	√			√	√	17.18	5,6,4'-Trihydroxy-3,7-dimethoxyflavone	L2a	$C_{17}H_{14}O_7$	1224	329.0667 (+0.1)	314.0438 (100), 271.0255 (67), 299.0209 (40), 329.0679 (14)	1158	331.0813 (+0.2)	331.0818 (100), 316.0582 (71), 301.0348 (20), 168.0052 (17), 273.0399 (16), 186.0162 (15), 245.0451 (9)	[136]
√	√	√	√	√	√	17.43	9,12,13-trihydroxyoctadeca-10,15-dienoic acid	L2a	$C_{18}H_{32}O_5$	1236	327.2177 (+0)	327.2177 (100), 229.1447 (55), 211.1331 (74), 171.1029 (32)	ND	ND	ND	[137]
	√					17.88	quercetin-3,7-dimethyl ether	L2a	$C_{17}H_{14}O_7$	1258	329.0664 (-0.8)	314.0420 (100), 243.0292 (69), 271.0235 (76), 329.0656 (34), 257.0473 (34), 299.0160 (27), 285.0402 (20)	1191	331.0818 (+1.7)	331.0819 (100), 109.0284 (74), 301.0587 (54), 127.0391 (30), 250.9387 (29), 57.0704 (18)	[138]
√	√	√	√			18.12	isomer 3-(4-hydroxyphenyl)-N-[3-[[3-(4-hydroxyphenyl)prop-2-enoyl]-[4-[[3-(4-hydroxyphenyl)prop-2-enoyl]-[3-[[3-(4-hydroxyphenyl)prop-2-enoyl]amino]propyl]amino]butyl]amino]propyl]prop-2-enamide	L2a	$C_{46}H_{50}N_4O_8$	1271	785.3553 (-0.4)	785.3551 (100), 545.2401 (95), 665.2974 (44), 119.0505 (10)	ND	ND	ND	[139]
√	√	√	√			18.43	isomer 3-(4-hydroxyphenyl)-N-[3-[[3-(4-hydroxyphenyl)prop-2-enoyl]-[4-[[3-(4-hydroxyphenyl)prop-2-enoyl]-[3-[[3-(4-hydroxyphenyl)prop-2-enoyl]amino]propyl]amino]butyl]amino]propyl]prop-2-enamide	L2a	$C_{46}H_{50}N_4O_8$	1281	785.3556 (+0)	545.2402 (100), 785.3542 (97), 665.2972 (62)	ND	ND	ND	[139]
√	√	√	√			18.80	isomer 3-(4-hydroxyphenyl)-N-[3-[[3-(4-hydroxyphenyl)prop-2-enoyl]-[4-[[3-(4-hydroxyphenyl)prop-2-enoyl]-[3-[[3-(4-hydroxyphenyl)prop-2-enoyl]amino]propyl]amino]butyl]amino]propyl]prop-2-enamide	L2a	$C_{46}H_{50}N_4O_8$	1291	785.3557 (+0.1)	785.3551 (100), 545.2408 (80), 665.2990 (57), 119.0502 (12)	ND	ND	ND	[139]
√	√	√	√	√	√	18.83	9,12,13-trihydroxyoctadec-10-enoic acid	L2a	C18H34O5	1293	329.2334 (+506.7)	329.2326 (100), 211.1334 (57), 229.1439 (43), 99.0812 (14), 183.1376 (10)	ND	ND	ND	[140]
√	√	√	√			19.06	N1,N5,N10,N14-tetra-trans-p-coumaroylspermine	L2a	$C_{46}H_{50}N_4O_8$	1303	785.3554 (-0.2)	785.3547 (100), 545.2404 (81), 665.2977 (55), 145.0296 (9)	1248	787.3712 (+1.3)	641.3351 (100), 787.3715 (28), 275.1760 (22), 204.1025 (17), 623.3253 (14)	[139]
				√	√	19.29	cirsimaritin isomer	L2a	$C_{17}H_{14}O_6$	1314	313.0718 (+0.1)	283.0245 (100), 298.0485 (58), 117.0339 (25), 313.0725 (25), 255.0294 (21), 163.0031 (15)	1261	315.0864 (+0.3)	250.1784 (100), 152.0241 (29), 169.9900 (25), 101.0607 (24), 134.0144 (21)	[141]

√						19.85	methacryl-dihydrohelenalin	L2a	$C_{19}H_{24}O_5$	ND	ND	ND	1307	333.1698 (+0.4)	247.1336 (100), 173.0966 (88), 201.1275 (73), 145.1012 (63), 229.1226 (24), 131.0856 (22), 173.1331 (22)	[34]
√						20.28	isobutyryl-dihydrohelenalin	L2a	$C_{19}H_{26}O_5$	ND	ND	ND	1337	335.1854 (+0.3)	247.1338 (100), 173.0969 (63), 201.1280 (52), 145.1019 (49), 229.1232 (34), 131.0863 (27), 219.1385 (18), 159.1168 (16), 123.0806 (14), 183.1168 (13), 109.0659 (13)	[34]
√						20.33	methacryl-helenalin	L2b	$C_{19}H_{22}O_5$	ND	ND	ND	1344	331.1542 (+0.6)	199.1120 (100), 245.1176 (85), 227.1075 (80), 135.0808 (61), 173.0965 (58), 184.0893 (32), 157.1005 (31), 181.1014 (30), 91.0548 (29), 159.0806 (28), 143.0858 (28), 107.0862 (24), 105.0704 (24), 175.0704 (24)	[34]
		√	√			20.39	3,5,4'-trihydroxy-6,7,8-trimethoxyflavone	L2b	$C_{18}H_{16}O_8$	1357	359.0774 (+0.4)	329.0305 (100), 344.0534 (80), 194.9933 (77), 179.97 (50), 359.0762 (39), 286.0109 (38), 210.0161 (29), 316.0585 (19)	1353	361.0919 (+0.3)	361.0922 (100), 303.0504 (69), 331.0454 (44), 362.0949 (24), 288.0266 (23), 313.0354 (23)	[142]
		√				20.43	pinocembrin	L2a	$C_{15}H_{12}O_4$	1362	255.0666 (+1.2)	255.0675 (100), 151.0049 (20), 65.0039 (19), 213.0562 (14)	1357	257.0813 (+1.8)	153.0180 (100), 257.0811 (81), 103.0543 (39), 234.9613 (34), 198.0017 (33)	[143]
	√					20.45	myoinositol,1,5-diangelate-4,6-diacetate	L3	$C_{20}H_{28}O_{10}$	1367	427.1607 (-0.6)	99.0455 (100), 224.9396 (63), 146.9606 (51), 174.9548 (50), 288.9351 (46), 114.9873 (30), 89.0230 (30), 160.9762 (28)	1361	429.176 (+1.1)	83.0490 (100), 55.0539 (4), 329.1223 (3), 369.1560 (2), 127.0388 (2)	[144]
		√	√	√	√	20.47	eupatilin	L2a	$C_{18}H_{16}O_7$	1364	343.0824 (+0.2)	313.0359 (100), 328.0592 (52), 298.0118 (46), 270.0177 (20), 343.0835 (17)	1364	345.097 (+0.3)	345.0961 (100), 315.0493 (84), 297.0393 (29), 215.0181 (16), 197.0082 (12), 287.0541 (8)	[145]
	√					20.58	myoinositol,1,6-diangelate-4,5-diacetate	L3	$C_{20}H_{28}O_{10}$	ND	ND	ND	1380	429.1762 (+1.6)	83.0490 (100), 55.0544 (7), 369.1558 (3), 329.1225 (3), 127.0393 (2)	[144]
		√	√			20.62	4-methylkaempferol	L1	$C_{16}H_{12}O_6$	1374	299.0563 (+0.6)	284.0329 (100), 110.0009 (12)	ND	ND	ND	[5]
		√	√			20.76	galangin	L1	$C_{15}H_{10}O_5$	1381	269.0457 (+0.6)	269.0457 (100), 211.0398 (4), 171.0450 (3)	1405	271.0601 (+0)	271.0600 (100), 153.0182 (18), 105.0340 (7), 141.0705 (4)	[146]
√						20.76	isobutyryl-helenalin	L2b	$C_{19}H_{24}O_5$	ND	ND	ND	1400	333.1699 (+0.7)	199.1118 (100), 245.1171 (83), 227.1071 (70), 173.0961 (51), 135.0801 (37), 171.1167 (23), 105.0704 (21), 159.0806 (18), 209.0958 (17), 109.0655 (17)	[34]

				√	√	20.97	5,2'-dihydroxy-6,7,8,6'-tetramethoxyflavone	L2a	$C_{19}H_{18}O_8$	1395	373.0928 (-0.2)	343.0459 (100), 358.0689 (91), 328.0230 (71), 285.0041 (26), 312.9982 (25), 300.0283 (24), 373.0929 (22)	1425	375.1075 (+0.1)	375.1071 (100), 345.0597 (93), 327.0493 (28), 197.0080 (11), 330.0346 (10), 215.0173 (10)	[147]
√						21.06	pectolinarigenin	L2a	$C_{17}H_{14}O_6$	1404	313.0718 (+0.1)	283.0242 (100), 298.0478 (100), 117.0343 (25), 163.0033 (24), 255.0283 (22), 313.0697 (13), 174.9551 (9)	1436	315.0865 (+0.6)	315.0870 (100), 300.0634 (80), 186.0165 (35), 168.0058 (32), 135.0445 (6)	[148]
	√					21.13	myoinositol-1-angelate-4,5-diacetate-6-(2-methylbutyrate)	L3	$C_{20}H_{30}O_{10}$	ND	ND	ND	1451	431.1919 (+1.7)	288.2907 (100), 106.0865 (34), 88.0757 (15)	[144]
	√					21.17	myoinositol-1-angelate-4,5-diacetate-isovalerate	L3	$C_{20}H_{30}O_{10}$	ND	ND	ND	1455	431.1918 (+1.5)	288.2907 (100), 106.0865 (34), 88.0757 (15)	[144]
√						21.17	tigloyl-dihydrohelenalin	L2b	$C_{20}H_{26}O_5$	ND	ND	ND	1459	347.1854 (+0.3)	247.1328 (100), 173.0960 (74), 83.0488 (62), 201.1275 (57), 145.1009 (34), 229.1228 (30), 131.0854 (23), 159.0802 (17), 219.1382 (15), 119.0858 (12), 109.0645 (12)	[34]
√						21.57	tigloyl-helenalin	L2b	$C_{20}H_{24}O_5$	ND	ND	ND	1493	345.1701 (+1.3)	83.0491 (100), 199.1117 (21), 245.1175 (19), 227.1068 (16), 55.0539 (10), 173.0963 (9), 84.0522 (8)	[34]
√						21.88	methylbutyryl-dihydrohelenalin	L2b	$C_{20}H_{28}O_5$	ND	ND	ND	1526	349.2012 (+0.7)	247.1331 (100), 173.0967 (67), 201.1277 (52, 145.1013 (49), 131.0859 (35), 229.1220 (32), 173.1338 (22), 159.0800 (19), 219.1380 (16), 155.0857 (15), 159.1169 (14)	[34]
√						22.00	dehydrophytosphingosine	L2a	$C_{18}H_{37}NO_3$	ND	ND	ND	1543	316.2847 (+0.3)	316.2848 (100), 60.0443 (74), 280.2638 (72), 298.2758 (63), 95.0858 (30), 109.1010 (22), 81.0697 (17), 281.2662 (12), 93.0697 (12)	[149]
√						22.04	isovaleryl-dihydrohelenalin	L2b	$C_{20}H_{28}O_5$	ND	ND	ND	1548	349.2012 (+0.7)	247.1329 (100), 173.0965 (57), 201.1278 (53), 145.1017 (33), 229.1227 (28), 173.1327 (25), 219.1386 (16), 131.0856 (15), 159.1174 (11), 119.0861 (10), 183.1166 (16)	[34]
		√	√			22.29	bisnorhelipyrone	L3	$C_{15}H_{16}O_6$	1485	291.0873 (-0.4)	95.0502 (100), 139.0400 (66)	1586	293.102 (+0.1)	141.0547 (100), 153.0548 (31), 125.0597 (7), 293.1025 (4)	[142]
√						22.29	methylbutyryl-helenalin	L2b	$C_{20}H_{26}O_5$	ND	ND	ND	1580	347.1854 (+0.3)	245.1180 (100), 199.1125 (95), 227.1069 (73), 57.0699 (56), 173.0968 (37), 135.0811 (33), 159.0809 (22), 85.0648 (21)	[34]

√						22.34	isovaleroyl-helenalin	L2b	$C_{20}H_{26}O_5$	ND	ND	ND	1602	347.1855 (+0.6)	245.1177 (100), 85.0650 (74), 199.1121 (61), 227.1063 (57), 173.0951 (31), 184.0880 (26), 159.0808 (25), 181.1007 (25), 171.1178 (24), 135.0810 (24), 109.0647 (23)	[34]
	√					22.57	eldarin	L3	$C_{17}H_{24}O_4$	1499	291.16 (-0.6)	59.0141 (100), 249.1495 (43), 83.0503 (37), 231.1392 (21), 205.1594 (18), 291.1595 (10)	1616	293.1749 (+0.6)	233.1540 (100), 151.0759 (85), 105.0703 (75), 81.0699 (60), 187.1488 (56), 91.0539 (48), 215.1434 (42), 161.1318 (41), 95.0861 (34), 153.0915 (32), 107.0852 (26)	[144]
	√					22.57	iso-alantolactone	L2a	$C_{15}H_{20}O_2$	ND	ND	ND	1619	233.1537 (+0.4)	105.0700 (100), 187.1480 (98), 91.0543 (90), 95.0855 (87), 161.1324 (86), 119.0857 (69), 131.0858 (66), 107.0856 (62), 81.0698 (60), 93.0699 (59)	[150]
		√	√			23.03	dadahol A	L2a	$C_{39}H_{38}O_{12}$	1529	697.2289 (-0.2)	163.0399 (100), 697.22272 (70), 145.0298 (29), 119.0501 (29), 112.9844 (13)	ND	ND	ND	[151]
	√					23.26	costunolide	L2a	$C_{15}H_{20}O_2$	ND	ND	ND	1678	233.1537 (+0.4)	187.1482 (100), 105.0701 (59), 131.0856 (51), 145.1011 (43), 91.0542 (39), 81.0698 (28), 107.0855 (26), 159.1166 (26), 95.0856 (25)	[152]
		√	√			23.54	3,5-dihydroxy-6,7,8-trimethoxyflavone	L2a	$C_{18}H_{16}O_7$	1562	343.0824 (+0.2)	313.0352 (100), 270.0179 (63), 328.0588 (34), 186.0321 (31), 285.0409 (21), 242.0218 (20), 298.0126 (16)	1709	345.0972 (+0.9)	345.0976 (100), 287.0553 (67), 315.0502 (47), 297.0398 (24), 272.0317 (20), 330.0739 (14), 269.0447 (12)	[142,153]
		√	√			23.95	norhelipyrone	L3	$C_{16}H_{18}O_6$	1588	305.103 (-0.2)	95.0501 (100), 109.0659 (91), 139.0401 (66), 153.0554 (60)	1743	307.1178 (+0.6)	141.0546 (100), 155.0703 (98), 167.0703 (28)	[142]
		√		√	√	23.98	carnosol	L2a	$C_{20}H_{26}O_4$	1596	329.1758 (-0.1)	285.1857 (100), 201.0919 (8), 270.1602 (2), 214.1020 (1)	1747	331.1906 (+0.6)	285.1849 (100), 215.1064 (79), 267.1750 (36), 243.1383 (28), 191.1065 (25), 331.1900 (23), 173.0603 (18)	[154]
√	√	√	√	√	√	24.37	stearidonic acid isomer	L2a	$C_{18}H_{28}O_2$	ND	ND	ND	1787	277.2163 (+0.3)	93.0698 (100), 135.1168 (71), 121.1014 (56), 107.0857 (53), 79.0541 (45), 91.0541 (33), 95.0852 (22), 149.1326 (22), 109.1015 (16)	[155]
√	√	√	√	√	√	24.37	stearidonic acid isomer	L2a	$C_{18}H_{28}O_2$	ND	ND	ND	1786	277.2164 (+0.7)	93.0699 (100), 135.1170 (87), 121.1009 (64), 107.0854 (57), 79.0542 (52), 91.0541 (46), 95.0854 (30), 149.1326 (25)	[155]

		√	√			24.49	arenol	L2a	$C_{21}H_{24}O_7$	1638	387.1451 (+0.4)	235.0982 (100), 247.0980 (88), 191.1080 (17), 139.0403 (15), 95.0504 (14)	1794	389.1599 (+1.1)	181.0497 (100), 141.0548 (47), 249.1122 (37), 193.0494 (36), 237.1123 (26), 333.0972 (25), 153.0548 (25), 163.0391 (15)	[78]
√						25.12	1-palmitoyl-2-hydroxy-sn-glycero-3-phosphoethanolamine	L2a	$C_{21}H_{44}NO_7P$	1694	452.2779 (-0.8)	255.2329 (100), 452.2765 (27), 112.9857 (25), 196.0376 (12)	1881	454.2932 (+0.8)	313.2740 (100), 282.2786 (21), 216.0625 (8), 98.9847 (8), 173.0213 (7)	[156]
			√			25.17	linolenic acid	L2a	$C_{18}H_{30}O_2$	ND	ND	ND	1897	279.2321 (+0.9)	95.0858 (100), 81.0699 (74), 109.1018 (50), 123.1167 (28), 279.0935 (27), 67.0544 (24), 137.1333 (16), 219.0582 (15)	[157]
	√		√			25.25	13-Hydroxy-9,11-octadecadienoic acid	L2a	$C_{18}H_{32}O_3$	1703	295.2276 (-0.9)	295.2273 (100), 277.2171 (48), 195.1388 (38), 114.9888 (8), 158.9792 (5)	ND	ND	ND	[158]
		√	√			25.29	heliarzanol 1	L2b	$C_{24}H_{30}O_8$	1709	445.1867 (-0.2)	295.2292 (100), 277.2180 (58), 195.1385 (42), 183.1025 (20), 116.9289 (11)	ND	ND	ND	[5]
		√	√			25.49	helipyrone A	L2a	$C_{17}H_{20}O_6$	1730	319.1185 (-0.7)	109.0655 (100), 153.0551 (57)	1932	321.1336 (+1)	155.0709 (100), 167.0710 (25), 139.0759 (6), 321.1340 (4)	[159]
		√	√	√		25.67	arzanol	L2a	$C_{22}H_{26}O_7$	1746	401.1606 (+0.1)	235.0972 (100), 247.0973 (91), 191.1074 (23), 109.0655 (20), 153.0553 (18), 205.0866 (11)	1965	403.1756 (+1.2)	181.0502 (100), 155.0709 (48), 193.0503 (43), 249.1130 (42), 237.1130 (34), 167.0709 (32), 347.1135 (26)	[159]
√	√	√	√	√	√	25.68	1-hexadecanoyl-sn-glycero-3-phospho-(1'-myo-inositol)	L2a	$C_{25}H_{49}O_{12}P$	1747	571.2885 (-0.7)	571.2893 (100), 255.2334 (65), 152.9959 (27), 241.0121 (23), 315.0491 (13)	ND	ND	ND	[160]
	√		√	√	√	25.87	13-Oxo-9,11-octadecadienoic acid isomer	L2a	$C_{18}H_{30}O_3$	ND	ND	ND	1991	295.2268 (+0.1)	151.1120 (100), 277.2160 (38), 81.0329 (33), 107.0856 (28), 95.0491 (27), 93.0698 (26), 109.1016 (25), 133.1015 (22), 83.0490 (19)	[161]
√	√	√	√	√	√	25.9	13-Oxo-9,11-octadecadienoic acid isomer	L2a	$C_{18}H_{30}O_3$	ND	ND	ND	1993	295.2269 (+0.4)	179.1426 (100), 99.0799 (52), 93.0696 (35), 135.1166 (31), 277.2156 (25), 121.1008 (24), 95.0855 (23), 107.0856 (20), 81.0692 (18)	[161]
√	√	√	√		√	25.98	13-Oxo-9,11-octadecadienoic acid isomer	L2a	$C_{18}H_{30}O_3$	ND	ND	ND	2005	295.2269 (+0.4)	151.1112 (100), 277.2157 (35), 81.0330 (29), 93.0696 (23), 81.0690 (22), 109.1009 (20), 95.0489 (19), 107.0850 (19), 133.1009 (17), 79.0537 (16)	[161]
		√	√			26.09	cycloarzanol	L2a	$C_{22}H_{26}O_7$	1790	401.1602 (-0.9)	249.1132 (100), 261.1135 (89), 205.1237 (16), 139.0401 (12), 95.0499 (10)	2018	403.1757 (+1.4)	195.0656 (100), 263.1283 (55), 141.0551 (54), 251.1279 (44), 177.0552 (44), 347.1128 (40), 153.0550 (34), 207.0656 (30), 189.0540 (25)	[5]

		√				26.28	pyrenophorol	L2a	$C_{13}H_{28}O_8$	1809	311.1681 (-9.8)	311.1686 (100), 183.0117 (68), 216.0091 (10)	ND	ND	ND	[162]
		√	√			26.3	arzanol derivative	L2a	$C_{31}H_{36}O_{10}$	1813	567.2233 (-0.5)	293.1393 (100), 153.0554 (51), 109.0659 (29), 235.0979 (17), 305.1389 (8), 174.9555 (5)	2046	569.2389 (+1.4)	223.1333 (100), 167.0701 (75), 155.0702 (67), 181.0495 (58), 347.1115 (38), 403.1773 (36), 249.1107 (29), 235.1326 (24), 415.1754 (22), 361.1646 (11), 193.0488 (11)	[5]
		√	√			26.68	heliarzanol isomer	L2b	$C_{24}H_{30}O_8$	1833	445.1862 (-1.3)	279.1240 (100), 153.0560 (55), 109.0660 (45), 193.0874 (44), 205.0869 (27), 180.1278 (18)	2088	447.2019 (+1.2)	167.0703 (100), 155.0704 (95), 213.0757 (70), 281.1388 (50), 293.1407 (33), 225.0760 (23), 379.1392 (23), 195.0652 (15)	[5]
		√	√			27.19	3-methylarzanol	L2a	$C_{23}H_{28}O_7$	1869	415.1763 (+0.2)	249.1131 (100), 261.1131 (82), 275.1288 (21), 205.1234 (14), 109.0657 (12)	2148	417.1912 (+1)	195.0652 (100), 155.0704 (45), 263.1276 (43), 251.1280 (38), 177.0548 (36), 167.0705 (34), 207.0653 (30), 361.1282 (24)	[5]
		√	√			28.11	pentahydroxy-neryl or -geranyl-dihydrochalcone	L3	$C_{25}H_{30}O_6$	1952	425.1968 (-0.4)	327.1602 (100), 203.0350 (24), 425.1964 (8)	2267	427.212 (+1.1)	303.0874 (100), 243.0660 (33), 261.0772 (17), 165.0554 (10), 219.0658 (8), 225.0556 (6)	[163]
		√	√			28.32	6-O-desmethyl-auricepyrone	L2a	$C_{24}H_{30}O_7$	1970	429.1919 (+0.1)	263.1285 (100), 275.1286 (89), 219.1386 (14), 109.0655 (13), 153.0552 (12), 429.1915 (10), 205.0866 (5)	2284	431.2068 (+0.9)	209.0817 (100), 155.0710 (61), 277.144 (57), 191.0711 (55), 265.1443 (47), 167.0709 (44), 203.0711 (37), 375.1449 (26), 221.0819 (23)	[78]
√	√	√	√			28.41	16-hydroxypalmitic acid	L2a	$C_{16}H_{32}O_3$	1980	271.2278 (-0.3)	225.2230 (100), 271.2290 (52), 253.2185 (4)	ND	ND	ND	[164]
√	√	√		√	√	28.71	linolenic acid	L2a	$C_{18}H_{30}O_2$	2013	277.2174 (+0.3)	277.2179 (100), 75.0085 (4), 182.9005 (3)	2331	279.2319 (+0.2)	81.0695 (100), 95.0855 (100), 109.1013 (85), 123.1168 (61), 279.2317 (24), 137.1324 (20), 67.0542 (19), 107.0860 (15)	[165]
		√	√			28.79	methylpyrone	L2a	$C_{26}H_{32}O_7$	2017	455.2079 (+0.8)	303.1606 (100), 315.1605 (98), 455.2077 (27), 259.1704 (21), 139.0401 (18)	2356	457.2226 (+1.1)	181.0497 (100), 141.0548 (64), 333.0973 (60), 193.0498 (33), 317.1752 (31), 305.1753 (28), 153.0547 (20), 163.0390 (12)	[142]
√	√			√	√	29.14	13-docosenamide	L2a	$C_{22}H_{43}NO$	ND	ND	ND	2393	338.3418 (+0.2)	338.3413 (100), 97.1006 (96), 83.0848 (64), 95.0850 (60), 111.1163 (56), 114.0908 (46), 100.0750 (45), 135.1166 (42), 321.3148 (40), 303.3037 (39)	[166]
		√	√			29.27	23-methyl-6-O-desmethylauricepyrone	L2a	$C_{25}H_{32}O_7$	2066	443.2076 (+0.2)	99.9262 (100), 44.9992 (72), 68.9956 (27), 115.9194 (12)	2404	445.2225 (+0.9)	223.0967 (100), 205.0862 (63), 155.0704 (62), 291.1594 (62), 279.1594 (51), 167.0704 (46), 217.0860 (44), 389.1597 (27), 235.0964 (23)	[159]

		√	√			29.52	italipyrone	L2b	$C_{22}H_{24}O_7$	2083	399.1448 (-0.3)	233.0820 (100), 109.0658 (21), 153.0558 (20), 245.0822 (17), 189.0913 (4)	2436	401.1597 (+0.5)	235.0967 (100), 247.0964 (74), 167.0701 (27), 155.0700 (18), 229.0857 (7)	[5]
√	√					29.55	santinol A1	L3	$C_{23}H_{32}O_3$	ND	ND	ND	2439	357.2425 (-0.1)	153.0547 (100), 125.0597 (9), 357.2433 (6), 149.1327 (4), 205.1954 (3)	[167]
		√	√			29.8	ethylpyrone (R1=CH3 ; R2 = geranyl ; R3 = CH2-CH3)	L2a	$C_{27}H_{34}O_7$	2107	469.2228 (-0.8)	303.1602 (100), 315.1597 (99), 469.2228 (36), 153.0553 (21), 259.1697 (21), 166.0266 (16)	2486	471.2383 (+1.2)	181.0496 (100), 155.0704 (61), 347.1128 (56), 317.1750 (33), 305.1750 (33), 193.0499 (32), 167.0705 (22)	[78]
		√	√			30.26	phloroglucinol pyrone derivative	L3	$C_{27}H_{34}O_7$	2149	469.2233 (+0.3)	303.1601 (100), 315.1599 (91), 469.2229 (23), 259.1701 (13)	2544	471.2381 (+0.8)	195.0653 (100), 141.0547 (76), 347.1132 (59), 331.1905 (28), 207.0655 (27), 319.1906 (26), 177.0546 (26), 153.0547 (20)	[142]
		√	√			31.14	isopropylpyrone	L2b	$C_{28}H_{36}O_7$	2241	483.2387 (-0.3)	317.1747 (100), 329.1747 (77), 483.2368 (20), 343.1907 (16), 153.0549 (10)	2626	485.2537 (+0.7)	195.0654 (100), 155.0703 (69), 361.1283 (59), 331.1907 (31), 319.1901 (30), 207.0653 (28), 177.0547 (24)	[78]

AM: *A. montana*, PM: *P. montanum*, HI: *H. italicum*, HS: *H. stoechas*, SH: *S. hortensis*, SM: *S. montana*

Rt: Retention time, CL: Confidence level; ID(-): MZmine ID in negative ionization mode, ID(+): MZmine ID in positive ionization mode, Ref: references

Table S2: Extraction yields

Pair	*Arnica* pair		*Helichrysum* pair		*Satureja* pair	
	Phytotherapy	Provence	Phytotherapy	Provence	Phytotherapy	Provence
Species	*Arnica montana*	*Pentanema montanum*	*Helichrysum italicum*	*Helichrysum stoechas*	*Satureja hortensis*	*Satureja montana*
Sample name	AM	PM	HI	HS	SH	SM
Extraction yield (in % *w/w*)	14.4 ± 1.3	11.0 ± 0.4	13.9 ± 1.1	13.7 ± 0.3	9.5 ± 1.4	12.2± 1.2

Data are expressed as the mean of triplicates ± standard deviation.

Table S3: MZmine parameters

**Raw data methods → Raw data import**	import all .mzXML files
**Raw data methods → Mass detection**	
Scan filters:	Scan MS level = 1, Polarity (Any), Spectrum type (ANY)
Mass detector:	Centroid
Noise level:	NEG : 3E2 ; POS : 1.5E2
m/z tolerance:	5.0E-4 m/z or 10.0 ppm
**Raw data methods → Mass detection**	
Scan filters:	Scan MS level = 2, Polarity (Any), Spectrum type (ANY)
Mass detector:	Centroid
Noise level:	NEG : 1E1 ; POS : 1E1
m/z tolerance:	5.0E-4 m/z or 10.0 ppm
**Feature detection → LC-MS → ADAP Chromatogram Builder**	
Scan filters:	Scan MS level = 1, Polarity (Any), Spectrum type (ANY)
Minimum consecutive scans:	5
Minimum intensity for consecutive scans:	NEG : 9E2 ; POS : 4.5E2
Minimum absolute height:	NEG : 3E3 ; POS : 1.5E3
m/z tolerance (scan-to-scan):	0.0 m/z or 10.0 ppm
**Feature detection → Smoothing**	
Smoothing algorithm:	Loess smoothing
**Feature detection → Chromatogram resolving → Local minimum feature resolver**	
Dimension:	Retention time
Chromatographic threshold:	0.95
Minimum search range RT/Mobility (absolute):	0.05
Minimum relative height:	0
Minimum absolute height:	NEG : 3E3 ; POS : 1.5E3
Min ratio of peak top/edge:	NEG : 1.8 ; POS : 1.85
Peak duration range (min/mobility):	0.04 - 0.8
Minimum scans (data points):	5
**Feature list methods → Isotopes → 13C isotope filter**	
m/z tolerance (intra-sample):	0.0 m/z or 10.0 ppm
Retention time tolerance:	0.1 minutes
Mobility tolerance:	false
Monotonic shape:	false

Maximum charge:	2
Representative isotope:	Most intense
Never remove feature with MS2:	true

Feature list methods → Alignment → Join aligner

m/z tolerance (sample-to-sample):	0.0 m/z or 10.0 ppm
Weight for m/z:	75
Retention time tolerance:	0.2 minutes
Weight for RT:	25
Mobility tolerance:	false
Mobility weight:	1
Require same charge state:	true
Require same ID:	false
Compare isotope pattern:	false
Compare spectra similarity:	false

Feature list methods → Feature list filtering → Filtering feature list rows

Minimum aligned features (samples):	3
Retention time:	0.3 - 32.5 minutes
Feature with MS2 scan:	true
Reset the feature number ID:	true

Feature list methods → Alignment → Join aligner

m/z tolerance (sample-to-sample):	0.0 m/z or 10.0 ppm
Weight for m/z:	75
Retention time tolerance:	0.2 minutes
Weight for RT:	25
Mobility tolerance:	false
Mobility weight:	1
Require same charge state:	true
Require same ID:	false
Compare isotope pattern:	false
Compare spectra similarity:	false

Feature list methods → Export feature list → Molecular Networking Files

Merge MS/MS:	true
Select spectra to merge:	across samples
m/z merge mode:	most intense
intensity merge mode:	sum intensities
Expected mass deviation:	0.0 m/z or 10.0 ppm
Cosine threshold (%):	70
Signal count threshold (%):	20
Isolation window offset (m/z):	0
Isolation window width (m/z):	3
m/z tolerance:	0.0 m/z or 10.0 ppm
Filter rows:	MS2 or ion identity
CSV export:	simple
Submit to GNPS:	false

Table S4: Feature-Based Molecular Networking (FBMN) parameters

The data were filtered by removing all MS/MS fragment ions within ± 17 Da of the precursor ion m/z. MS/MS spectra were window filtered by keeping only the top 6 fragment ions in the ± 50 Da window throughout the spectrum. The precursor ion mass tolerance was set to 0.02 Da, just as the MS/MS fragment ion. Molecular networks were then created, with edges filtered to have a cosine score above 0.7 and more than 6 matched peaks. Further, edges between two nodes were kept in the network if and only if each of the nodes appeared in each other's respective top 10 most similar nodes. Finally, the maximum size of a molecular family was set to 100, and the lowest scoring edges were removed from molecular families until the molecular family size was below this threshold.

References

1. CCMSLIB00010102416 Available online: http://gnps.ucsd.edu/ProteoSAFe/gnpslibraryspectrum.jsp?SpectrumID=CCMSLIB00010102416.
2. MSBNK-RIKEN-PR309121 Available online: https://massbank.eu/MassBank/RecordDisplay?id=MSBNK-RIKEN-PR309121&dsn=RIKEN.
3. Xiao, Y.; Vecchi, M.M.; Wen, D. Distinguishing between Leucine and Isoleucine by Integrated LC–MS Analysis Using an Orbitrap Fusion Mass Spectrometer. *Anal. Chem.* **2016**, *88*, 10757–10766, doi:10.1021/acs.analchem.6b03409.
4. CCMSLIB00000479728 Available online: http://gnps.ucsd.edu/ProteoSAFe/gnpslibraryspectrum.jsp?SpectrumID=CCMSLIB00000479728.
5. Kramberger, K.; Barlič-Maganja, D.; Bandelj, D.; Baruca Arbeiter, A.; Peeters, K.; Miklavčič Višnjevec, A.; Jenko Pražnikar, Z. HPLC-DAD-ESI-QTOF-MS Determination of Bioactive Compounds and Antioxidant Activity Comparison of the Hydroalcoholic and Water Extracts from Two *Helichrysum Italicum* Species. *Metabolites* **2020**, *10*, 403, doi:10.3390/metabo10100403.
6. CCMSLIB00004694530 Available online: http://gnps.ucsd.edu/ProteoSAFe/gnpslibraryspectrum.jsp?SpectrumID=CCMSLIB00004694530.
7. Clifford, M.N.; Johnston, K.L.; Knight, S.; Kuhnert, N. Hierarchical Scheme for LC-MSn Identification of Chlorogenic Acids. *J. Agric. Food Chem.* **2003**, *51*, 2900–2911, doi:10.1021/jf026187q.
8. Splash10-004i-0901000000-4baa83a9d2df1615a0bb Available online: https://mona.fiehnlab.ucdavis.edu/spectra/display/PR100940.
9. Splash10-0006-0900000000-04c43c428d4dd459249d Available online: https://mona.fiehnlab.ucdavis.edu/spectra/display/MoNA042081.
10. Velamuri, R.; Sharma, Y.; Fagan, J.; Schaefer, J. Application of UHPLC-ESI-QTOF-MS in Phytochemical Profiling of Sage (*Salvia Officinalis*) and Rosemary (*Rosmarinus Officinalis*). *Planta Medica International Open* **2020**, *07*, e133–e144, doi:10.1055/a-1272-2903.
11. CCMSLIB00004696262 Available online: http://gnps.ucsd.edu/ProteoSAFe/gnpslibraryspectrum.jsp?SpectrumID=CCMSLIB00004696262 (accessed on 9 December 2024).
12. CCMSLIB00005467742 Available online: http://gnps.ucsd.edu/ProteoSAFe/gnpslibraryspectrum.jsp?SpectrumID=CCMSLIB00005467742 (accessed on 9 December 2024).

13. Kamel, M.S.; Assaf, M.H.; Hasanean, H.A.; Ohtani, K.; Kasai, R.; Yamasaki, K. Monoterpene Glucosides from *Origanum Syriacum*. *Phytochemistry* **2001**, *58*, 1149–1152, doi:10.1016/S0031-9422(01)00386-7.
14. SWYRVCGNMNAFEK-MHXFFUGFSA-N%27) Available online: https://mona.fiehnlab.ucdavis.edu/spectra/display/CCMSLIB00000579260 (accessed on 9 December 2024).
15. Splash10-000i-0900000000-34715572970bc6edd136 Available online: https://mona.fiehnlab.ucdavis.edu/spectra/display/VF-NPL-QTOF000015.
16. Moreira, S.A.; Silva, S.; Costa, E.; Pinto, S.; Sarmento, B.; Saraiva, J.A.; Pintado, M. Effect of High Hydrostatic Pressure Extraction on Biological Activities and Phenolics Composition of Winter Savory Leaf Extracts. *Antioxidants* **2020**, *9*, 841, doi:10.3390/antiox9090841.
17. Peng, J.; Xie, J.; Shi, S.; Luo, L.; Li, K.; Xiong, P.; Cai, W. Diagnostic Fragment-Ion-Based for Rapid Identification of Chlorogenic Acids Derivatives in Inula Cappa Using UHPLC-Q-Exactive Orbitrap Mass Spectrometry. *Journal of Analytical Methods in Chemistry* **2021**, *2021*, 6393246, doi:10.1155/2021/6393246.
18. CCMSLIB00004710948 Available online: http://gnps.ucsd.edu/ProteoSAFe/gnpslibraryspectrum.jsp?SpectrumID=CCMSLIB0000471094 8 (accessed on 9 December 2024).
19. Clifford, M.N.; Knight, S.; Kuhnert, N. Discriminating between the Six Isomers of Dicaffeoylquinic Acid by LC-MSn. *J. Agric. Food Chem.* **2005**, *53*, 3821–3832, doi:10.1021/jf050046h.
20. Jaiswal, R.; Kuhnert, N. Identification and Characterization of Two New Derivatives of Chlorogenic Acids in Arnica (*Arnica Montana* L.) Flowers by High-Performance Liquid Chromatography/Tandem Mass Spectrometry. *J Agric Food Chem* **2011**, *59*, 4033–4039, doi:10.1021/jf103545k.
21. CCMSLIB00004697308 Available online: http://gnps.ucsd.edu/ProteoSAFe/gnpslibraryspectrum.jsp?SpectrumID=CCMSLIB0000469730 8 (accessed on 9 December 2024).
22. CCMSLIB00004695990 Available online: http://gnps.ucsd.edu/ProteoSAFe/gnpslibraryspectrum.jsp?SpectrumID=CCMSLIB0000469599 0 (accessed on 9 December 2024).
23. Cao, S.; Hu, M.; Yang, L.; Li, M.; Shi, Z.; Cheng, W.; Zhang, Y.; Chen, F.; Wang, S.; Zhang, Q. Chemical Constituent Analysis of Ranunculus Sceleratus L. Using Ultra-High-Performance Liquid Chromatography Coupled with Quadrupole-Orbitrap High-Resolution Mass Spectrometry. *Molecules* **2022**, *27*, 3299, doi:10.3390/molecules27103299.
24. CCMSLIB00004678820 Available online: http://gnps.ucsd.edu/ProteoSAFe/gnpslibraryspectrum.jsp?SpectrumID=CCMSLIB0000467882 0 (accessed on 9 December 2024).
25. Ali, A.; Cottrell, J.J.; Dunshea, F.R. Characterization, Antioxidant Potential, and Pharmacokinetics Properties of Phenolic Compounds from Native Australian Herbs and Fruits. *Plants* **2023**, *12*, 993.
26. CCMSLIB00000845356 Available online: http://gnps.ucsd.edu/ProteoSAFe/gnpslibraryspectrum.jsp?SpectrumID=CCMSLIB0000084535 6 (accessed on 9 December 2024).
27. CCMSLIB00000222374 Available online: http://gnps.ucsd.edu/ProteoSAFe/gnpslibraryspectrum.jsp?SpectrumID=CCMSLIB0000022237 4 (accessed on 9 December 2024).
28. XEVQXKKKAVVSMW-WRWORJQWSA-N%27) Available online: https://mona.fiehnlab.ucdavis.edu/spectra/browse?query=exists(compound.metaData.name:%27InChIKey%27%20and%20compound.metaData.value:%27XEVQXKKKAVVSMW-WRWORJQWSA-N%27).

29. CCMSLIB00004718321 Available online: http://gnps.ucsd.edu/ProteoSAFe/gnpslibraryspectrum.jsp?SpectrumID=CCMSLIB00004718321 (accessed on 9 December 2024).
30. CCMSLIB00010123659 Available online: http://gnps.ucsd.edu/ProteoSAFe/gnpslibraryspectrum.jsp?SpectrumID=CCMSLIB00010123659 (accessed on 9 December 2024).
31. Panusa, A.; Petrucci, R.; Lavecchia, R.; Zuorro, A. UHPLC-PDA-ESI-TOF/MS Metabolic Profiling and Antioxidant Capacity of Arabica and Robusta Coffee Silverskin: Antioxidants vs Phytotoxins. *Food Research International* **2017**, *99*, 155–165, doi:10.1016/j.foodres.2017.05.017.
32. CCMSLIB00011430375 Available online: http://gnps.ucsd.edu/ProteoSAFe/gnpslibraryspectrum.jsp?SpectrumID=CCMSLIB00011430375 (accessed on 9 December 2024).
33. Wang, C.Z.; Yu, D.Q. Lignan and Acetylenic Glycosides from *Aster Auriculatus*. *Phytochemistry* **1998**, *48*, 711–717, doi:10.1016/S0031-9422(98)00019-3.
34. Perry, N.; Burgess, E.; Rodríguez Guitián, M.; Romero Franco, R.; López Mosquera, E.; Smallfield, B.; Joyce, N.; Littlejohn, R. Sesquiterpene Lactones in *Arnica Montana* : Helenalin and Dihydrohelenalin Chemotypes in Spain. *Planta Med* **2009**, *75*, 660–666, doi:10.1055/s-0029-1185362.
35. CCMSLIB00005747116 Available online: http://gnps.ucsd.edu/ProteoSAFe/gnpslibraryspectrum.jsp?SpectrumID=CCMSLIB00005747116 (accessed on 9 December 2024).
36. CCMSLIB00005741453 Available online: http://gnps.ucsd.edu/ProteoSAFe/gnpslibraryspectrum.jsp?SpectrumID=CCMSLIB00005741453 (accessed on 9 December 2024).
37. CCMSLIB00004706229 Available online: http://gnps.ucsd.edu/ProteoSAFe/gnpslibraryspectrum.jsp?SpectrumID=CCMSLIB00004706229 (accessed on 9 December 2024).
38. CCMSLIB00005778054 Available online: http://gnps.ucsd.edu/ProteoSAFe/gnpslibraryspectrum.jsp?SpectrumID=CCMSLIB00005778054 (accessed on 9 December 2024).
39. Li, L.; Feng, R.; Feng, X.; Chen, Y.; Liu, X.; Sun, W.; Zhang, L. The Development and Validation of an HPLC-MS/MS Method for the Determination of Eriocitrin in Rat Plasma and Its Application to a Pharmacokinetic Study. *RSC Adv* **2020**, *10*, 10552–10558, doi:10.1039/c9ra10925k.
40. CCMSLIB00005749683 Available online: http://gnps.ucsd.edu/ProteoSAFe/gnpslibraryspectrum.jsp?SpectrumID=CCMSLIB00005749683 (accessed on 9 December 2024).
41. CCMSLIB00004706230 Available online: http://gnps.ucsd.edu/ProteoSAFe/gnpslibraryspectrum.jsp?SpectrumID=CCMSLIB00004706230 (accessed on 9 December 2024).
42. CCMSLIB00005743117 Available online: http://gnps.ucsd.edu/ProteoSAFe/gnpslibraryspectrum.jsp?SpectrumID=CCMSLIB00005743117 (accessed on 9 December 2024).
43. CCMSLIB00011430423 Available online: http://gnps.ucsd.edu/ProteoSAFe/gnpslibraryspectrum.jsp?SpectrumID=CCMSLIB00011430423 (accessed on 9 December 2024).
44. CCMSLIB00011430275 Available online: http://gnps.ucsd.edu/ProteoSAFe/gnpslibraryspectrum.jsp?SpectrumID=CCMSLIB00011430275 (accessed on 9 December 2024).
45. CCMSLIB00004704384 Available online: http://gnps.ucsd.edu/ProteoSAFe/gnpslibraryspectrum.jsp?SpectrumID=CCMSLIB00004704384 (accessed on 9 December 2024).

46. CCMSLIB00005778052 Available online: http://gnps.ucsd.edu/ProteoSAFe/gnpslibraryspectrum.jsp?SpectrumID=CCMSLIB00005778052 (accessed on 9 December 2024).
47. Llorent-Martínez, E.J.; Spínola, V.; Gouveia, S.; Castilho, P.C. HPLC-ESI-MSn Characterization of Phenolic Compounds, Terpenoid Saponins, and Other Minor Compounds in *Bituminaria Bituminosa*. *Industrial Crops and Products* **2015**, *69*, 80–90, doi:10.1016/j.indcrop.2015.02.014.
48. CCMSLIB00005747752 Available online: http://gnps.ucsd.edu/ProteoSAFe/gnpslibraryspectrum.jsp?SpectrumID=CCMSLIB00005747752 (accessed on 9 December 2024).
49. CCMSLIB00004702995 Available online: http://gnps.ucsd.edu/ProteoSAFe/gnpslibraryspectrum.jsp?SpectrumID=CCMSLIB00004702995 (accessed on 9 December 2024).
50. Ancillotti, C.; Ciofi, L.; Rossini, D.; Chiuminatto, U.; Stahl-Zeng, J.; Orlandini, S.; Furlanetto, S.; Del Bubba, M. Liquid Chromatographic/Electrospray Ionization Quadrupole/Time of Flight Tandem Mass Spectrometric Study of Polyphenolic Composition of Different *Vaccinium* Berry Species and Their Comparative Evaluation. *Anal Bioanal Chem* **2017**, *409*, 1347–1368, doi:10.1007/s00216-016-0067-y.
51. Wang, Z.-L.; Wang, S.; Kuang, Y.; Hu, Z.-M.; Qiao, X.; Ye, M. A Comprehensive Review on Phytochemistry, Pharmacology, and Flavonoid Biosynthesis of Scutellaria Baicalensis. *Pharmaceutical Biology* **2018**, *56*, 465–484, doi:10.1080/13880209.2018.1492620.
52. CCMSLIB00005739929 Available online: http://gnps.ucsd.edu/ProteoSAFe/gnpslibraryspectrum.jsp?SpectrumID=CCMSLIB00005739929 (accessed on 9 December 2024).
53. Bitam, F.; Letizia Ciavatta, M.; Manzo, E.; Dibi, A.; Gavagnin, M. Chemical Characterisation of the Terpenoid Constituents of the Algerian Plant *Launaea Arborescens*. *Phytochemistry* **2008**, *69*, 2984–2992, doi:10.1016/j.phytochem.2008.09.025.
54. CCMSLIB00004678828 Available online: http://gnps.ucsd.edu/ProteoSAFe/gnpslibraryspectrum.jsp?SpectrumID=CCMSLIB00004678828.
55. CCMSLIB00004720252 Available online: http://gnps.ucsd.edu/ProteoSAFe/gnpslibraryspectrum.jsp?SpectrumID=CCMSLIB00004720252.
56. CCMSLIB00004702808 Available online: http://gnps.ucsd.edu/ProteoSAFe/gnpslibraryspectrum.jsp?SpectrumID=CCMSLIB00004702808 (accessed on 9 December 2024).
57. CCMSLIB00010124979 Available online: http://gnps.ucsd.edu/ProteoSAFe/gnpslibraryspectrum.jsp?SpectrumID=CCMSLIB00010124979 (accessed on 9 December 2024).
58. Splash10-004i-0900000000-6082780274b24b6c6862 Available online: https://mona.fiehnlab.ucdavis.edu/spectra/display/RIKENPlaSMA008141.
59. CCMSLIB00005467729 Available online: http://gnps.ucsd.edu/ProteoSAFe/gnpslibraryspectrum.jsp?SpectrumID=CCMSLIB00005467729.
60. CCMSLIB00004706631 Available online: http://gnps.ucsd.edu/ProteoSAFe/gnpslibraryspectrum.jsp?SpectrumID=CCMSLIB00004706631.
61. CCMSLIB00004711941 Available online: http://gnps.ucsd.edu/ProteoSAFe/gnpslibraryspectrum.jsp?SpectrumID=CCMSLIB00004711941.

62. CCMSLIB00005742298 Available online: http://gnps.ucsd.edu/ProteoSAFe/gnpslibraryspectrum.jsp?SpectrumID=CCMSLIB00005742298.
63. CCMSLIB00003138125 Available online: http://gnps.ucsd.edu/ProteoSAFe/gnpslibraryspectrum.jsp?SpectrumID=CCMSLIB00003138125.
64. CCMSLIB00000222706 Available online: http://gnps.ucsd.edu/ProteoSAFe/gnpslibraryspectrum.jsp?SpectrumID=CCMSLIB00000222706.
65. CCMSLIB00005744426 Available online: http://gnps.ucsd.edu/ProteoSAFe/gnpslibraryspectrum.jsp?SpectrumID=CCMSLIB00005744426.
66. CCMSLIB00004706184 Available online: http://gnps.ucsd.edu/ProteoSAFe/gnpslibraryspectrum.jsp?SpectrumID=CCMSLIB00004706184.
67. CCMSLIB00005743792 Available online: http://gnps.ucsd.edu/ProteoSAFe/gnpslibraryspectrum.jsp?SpectrumID=CCMSLIB00005743792.
68. Pikulski, M.; Brodbelt, J.S. Differentiation of Flavonoid Glycoside Isomers by Using Metal Complexation and Electrospray Ionization Mass Spectrometry. *J. Am. Soc. Mass Spectrom.* **2003**, *14*, 1437–1453, doi:10.1016/j.jasms.2003.07.002.
69. CCMSLIB00004705059 Available online: http://gnps.ucsd.edu/ProteoSAFe/gnpslibraryspectrum.jsp?SpectrumID=CCMSLIB00004705059.
70. CCMSLIB00011430427 Available online: http://gnps.ucsd.edu/ProteoSAFe/gnpslibraryspectrum.jsp?SpectrumID=CCMSLIB00011430427.
71. CCMSLIB00005741560 Available online: http://gnps.ucsd.edu/ProteoSAFe/gnpslibraryspectrum.jsp?SpectrumID=CCMSLIB00005741560.
72. CCMSLIB00006581972 Available online: http://gnps.ucsd.edu/ProteoSAFe/gnpslibraryspectrum.jsp?SpectrumID=CCMSLIB00006581972.
73. CCMSLIB00005745089 Available online: http://gnps.ucsd.edu/ProteoSAFe/gnpslibraryspectrum.jsp?SpectrumID=CCMSLIB00005745089.
74. CCMSLIB00005744674 Available online: http://gnps.ucsd.edu/ProteoSAFe/gnpslibraryspectrum.jsp?SpectrumID=CCMSLIB00005744674.
75. CCMSLIB00005744999 Available online: http://gnps.ucsd.edu/ProteoSAFe/gnpslibraryspectrum.jsp?SpectrumID=CCMSLIB00005744999.
76. Rodríguez-Ferreiro, A.O.; Ochoa-Pacheco, A.; Méndez-Rodriguez, D.; Ortiz-Beatón, E.; Font-Salmo, O.; Guisado-Bourzac, F.; Molina-Bertrán, S.; Monzote, L.; Cos, P.; Foubert, K.; et al. LC-MS Characterization and Biological Activities of Cuban Cultivars of Plectranthus Neochilus Schltr. *Plants* **2022**, *11*, 134, doi:10.3390/plants11010134.
77. CCMSLIB00010121591 Available online: http://gnps.ucsd.edu/ProteoSAFe/gnpslibraryspectrum.jsp?SpectrumID=CCMSLIB00010121591.
78. Gevrenova, R.; Kostadinova, I.; Stefanova, A.; Balabanova, V.; Zengin, G.; Zheleva-Dimitrova, D.; Momekov, G. Phytochemical Profiling, Antioxidant and Cognitive-Enhancing Effect of

Helichrysum Italicum Ssp. Italicum (Roth) G. Don (Asteraceae). *Plants* **2023**, *12*, 2755, doi:10.3390/plants12152755.
79. CCMSLIB00005743168 Available online: http://gnps.ucsd.edu/ProteoSAFe/gnpslibraryspectrum.jsp?SpectrumID=CCMSLIB00005743168.
80. CCMSLIB00005741664 Available online: http://gnps.ucsd.edu/ProteoSAFe/gnpslibraryspectrum.jsp?SpectrumID=CCMSLIB00005741664.
81. CCMSLIB00005749533 Available online: http://gnps.ucsd.edu/ProteoSAFe/gnpslibraryspectrum.jsp?SpectrumID=CCMSLIB00005749533.
82. CCMSLIB00010113016 Available online: http://gnps.ucsd.edu/ProteoSAFe/gnpslibraryspectrum.jsp?SpectrumID=CCMSLIB00010113016.
83. CCMSLIB00010123259 Available online: http://gnps.ucsd.edu/ProteoSAFe/gnpslibraryspectrum.jsp?SpectrumID=CCMSLIB00010123259.
84. CCMSLIB00005740573 Available online: https://metabolomics-usi.ucsd.edu/spectrum/?usi=mzspec:GNPS:MASSBANK:accession:CCMSLIB00005740573.
85. CCMSLIB00005742039 Available online: http://gnps.ucsd.edu/ProteoSAFe/gnpslibraryspectrum.jsp?SpectrumID=CCMSLIB00005742039.
86. MSBNK-BS-BS003401 Available online: https://massbank.eu/MassBank/RecordDisplay?id=MSBNK-BS-BS003401&dsn=BS.
87. CCMSLIB00004678839 Available online: http://gnps.ucsd.edu/ProteoSAFe/gnpslibraryspectrum.jsp?SpectrumID=CCMSLIB00004678839.
88. CCMSLIB00005742082 Available online: http://gnps.ucsd.edu/ProteoSAFe/gnpslibraryspectrum.jsp?SpectrumID=CCMSLIB00005742082.
89. CCMSLIB00004706112 Available online: http://gnps.ucsd.edu/ProteoSAFe/gnpslibraryspectrum.jsp?SpectrumID=CCMSLIB00004706112.
90. CCMSLIB00005739282 Available online: http://gnps.ucsd.edu/ProteoSAFe/gnpslibraryspectrum.jsp?SpectrumID=CCMSLIB00005739282.
91. CCMSLIB00005739575 Available online: http://gnps.ucsd.edu/ProteoSAFe/gnpslibraryspectrum.jsp?SpectrumID=CCMSLIB00005739575.
92. CCMSLIB00004717548 Available online: http://gnps.ucsd.edu/ProteoSAFe/gnpslibraryspectrum.jsp?SpectrumID=CCMSLIB00004717548.
93. CCMSLIB00004702896 Available online: http://gnps.ucsd.edu/ProteoSAFe/gnpslibraryspectrum.jsp?SpectrumID=CCMSLIB00004702896.
94. CCMSLIB00004702903 Available online: http://gnps.ucsd.edu/ProteoSAFe/gnpslibraryspectrum.jsp?SpectrumID=CCMSLIB00004702903.
95. CCMSLIB00011430424 Available online: http://gnps.ucsd.edu/ProteoSAFe/gnpslibraryspectrum.jsp?SpectrumID=CCMSLIB00011430424.

96. Duthen, S.; Gadéa, A.; Trempat, P.; Boujedaini, N.; Fabre, N. Comparison of the Phytochemical Variation of Non-Volatile Metabolites within Mother Tinctures of *Arnica Montana* Prepared from Fresh and Dried Whole Plant Using UHPLC-HRMS Fingerprinting and Chemometric Analysis. *Molecules* **2022**, *27*, 2737, doi:10.3390/molecules27092737.
97. CCMSLIB00006581970 Available online: http://gnps.ucsd.edu/ProteoSAFe/gnpslibraryspectrum.jsp?SpectrumID=CCMSLIB00006581970.
98. CCMSLIB00004711458 Available online: http://gnps.ucsd.edu/ProteoSAFe/gnpslibraryspectrum.jsp?SpectrumID=CCMSLIB00004711458.
99. YFZSQPRYLBGYKE-FZFRBNDOSA-N%27) Available online: https://mona.fiehnlab.ucdavis.edu/spectra/browse?query=exists(compound.metaData.name:%27InChIKey%27%20and%20compound.metaData.value:%27YFZSQPRYLBGYKE-FZFRBNDOSA-N%27).
100. Wu, Y.-T.; Chen, Y.-F.; Hsieh, Y.-J.; Jaw, I.; Shiao, M.-S.; Tsai, T.-H. Bioavailability of Salvianolic Acid B in Conscious and Freely Moving Rats. *Int J Pharm* **2006**, *326*, 25–31, doi:10.1016/j.ijpharm.2006.07.003.
101. MSBNK-MetaboLights-ML003601 Available online: https://massbank.eu/MassBank/RecordDisplay?id=MSBNK-MetaboLights-ML003601&dsn=MetaboLights.
102. Marzouk, M.M.; Hussein, S.R.; Elkhateeb, A.; El-shabrawy, M.; Abdel-Hameed, E.-S.S.; Kawashty, S.A. Comparative Study of Mentha Species Growing Wild in Egypt: LC-ESI-MS Analysis and Chemosystematic Significance. *J App Pharm Sci* **2018**, *8,* 116–122, doi:10.7324/JAPS.2018.8816.
103. CCMSLIB00005745915 Available online: http://gnps.ucsd.edu/ProteoSAFe/gnpslibraryspectrum.jsp?SpectrumID=CCMSLIB00005745915.
104. Martins, N.; Barros, L.; Santos-Buelga, C.; Silva, S.; Henriques, M.; Ferreira, I.C. Decoction, Infusion and Hydroalcoholic Extract of Cultivated Thyme: Antioxidant and Antibacterial Activities, and Phenolic Characterisation. *Food chemistry* **2015**, *167*, 131–137.
105. CCMSLIB00000845756 Available online: http://gnps.ucsd.edu/ProteoSAFe/gnpslibraryspectrum.jsp?SpectrumID=CCMSLIB00000845756.
106. Kheyar-Kraouche, N.; da Silva, A.B.; Serra, A.T.; Bedjou, F.; Bronze, M.R. Characterization by Liquid Chromatography–Mass Spectrometry and Antioxidant Activity of an Ethanolic Extract of *Inula Viscosa* Leaves. *Journal of Pharmaceutical and Biomedical Analysis* **2018**, *156*, 297–306, doi:10.1016/j.jpba.2018.04.047.
107. CCMSLIB00006581954 Available online: http://gnps.ucsd.edu/ProteoSAFe/gnpslibraryspectrum.jsp?SpectrumID=CCMSLIB00006581954.
108. CCMSLIB00004691728#%7B%7D Available online: https://gnps.ucsd.edu/ProteoSAFe/gnpslibraryspectrum.jsp?SpectrumID=CCMSLIB00004691728#%7B%7D.
109. CCMSLIB00000077235 Available online: http://gnps.ucsd.edu/ProteoSAFe/gnpslibraryspectrum.jsp?SpectrumID=CCMSLIB00000077235.
110. CCMSLIB00010012027 Available online: http://gnps.ucsd.edu/ProteoSAFe/gnpslibraryspectrum.jsp?SpectrumID=CCMSLIB00010012027.
111. CCMSLIB00004718307 Available online: http://gnps.ucsd.edu/ProteoSAFe/gnpslibraryspectrum.jsp?SpectrumID=CCMSLIB00004718307.

112. Singh, A.; Singh, P.; Kumar, B.; Kumar, S.; Dev, K.; Maurya, R. Detection of Flavonoids from Spinacia Oleracea Leaves Using HPLC-ESI-QTOF-MS/MS and UPLC-QqQLIT-MS/MS Techniques. *Natural Product Research* **2019**, *33*, 2253–2256, doi:10.1080/14786419.2018.1489395.
113. Piątczak, E.; Kolniak-Ostek, J.; Gonciarz, W.; Lisiecki, P.; Kalinowska-Lis, U.; Szemraj, M.; Chmiela, M.; Zielińska, S. The Effect of Salvia Tomentosa Miller Extracts, Rich in Rosmarinic, Salvianolic and Lithospermic Acids, on Bacteria Causing Opportunistic Infections. *Molecules* **2024**, *29*, 590, doi:10.3390/molecules29030590.
114. Xia, M.; Ma, S.; Wang, Y.; Chen, D.; Jiang, L.; Wen, C.; Wu, G.; Wang, X. An Innovative UPLC-MS/MS Method for the Quantitation and Pharmacokinetics of Eupafolin in Rat Plasma. *Journal of Chromatography B* **2024**, *1245*, 124272, doi:10.1016/j.jchromb.2024.124272.
115. Tsukui, A.; Vendramini, P.H.; Garrett, R.; Scholz, M.B.S.; Eberlin, M.N.; Bizzo, H.R.; Rezende, C.M. Direct-Infusion Electrospray Ionization-Mass Spectrometry Analysis Reveals Atractyligenin Derivatives as Potential Markers for Green Coffee Postharvest Discrimination. *LWT* **2019**, *103*, 205–211, doi:10.1016/j.lwt.2018.12.078.
116. CCMSLIB00004718325 Available online: http://gnps.ucsd.edu/ProteoSAFe/gnpslibraryspectrum.jsp?SpectrumID=CCMSLIB00004718325.
117. Zengin, G.; Nilofar; Yildiztugay, E.; Bouyahya, A.; Cavusoglu, H.; Gevrenova, R.; Zheleva-Dimitrova, D. A Comparative Study on UHPLC-HRMS Profiles and Biological Activities of Inula Sarana Different Extracts and Its Beta-Cyclodextrin Complex: Effective Insights for Novel Applications. *Antioxidants* **2023**, *12*, 1842.
118. Nair, A.G.R.; Jayaprakasam, R.; Gunasekaran, R.; Bayet, C.; Voirin, B. 6-Hydroxykaempferol 7-(6″-Caffeoylglucoside) from *Eupatorium Glandulosum*. *Phytochemistry* **1993**, *33*, 1275–1276, doi:10.1016/0031-9422(93)85066-Z.
119. Petrakis, E.A.; Mikropoulou, E.V.; Mitakou, S.; Halabalaki, M.; Kalpoutzakis, E. A GC–MS and LC–HRMS Perspective on the Chemotaxonomic Investigation of the Natural Hybrid Origanum × Lirium and Its Parents, Subsp. Hirtum and O. Scabrum. *Phytochemical Analysis* **2023**, *34*, 289–300, doi:10.1002/pca.3206.
120. CCMSLIB00004706209 Available online: http://gnps.ucsd.edu/ProteoSAFe/gnpslibraryspectrum.jsp?SpectrumID=CCMSLIB00004706209.
121. CCMSLIB00004704874 Available online: http://gnps.ucsd.edu/ProteoSAFe/gnpslibraryspectrum.jsp?SpectrumID=CCMSLIB00004704874.
122. Lin, L.-Z.; Harnly, J.M. Identification of Hydroxycinnamoylquinic Acids of *Arnica* Flowers and Burdock Roots Using a Standardized LC-DAD-ESI/MS Profiling Method. *J Agric Food Chem* **2008**, *56*, 10105–10114, doi:10.1021/jf802412m.
123. CCMSLIB00000845611 Available online: http://gnps.ucsd.edu/ProteoSAFe/gnpslibraryspectrum.jsp?SpectrumID=CCMSLIB00000845611.
124. CCMSLIB00004678846 Available online: http://gnps.ucsd.edu/ProteoSAFe/gnpslibraryspectrum.jsp?SpectrumID=CCMSLIB00004678846.
125. CCMSLIB00004718271 Available online: http://gnps.ucsd.edu/ProteoSAFe/gnpslibraryspectrum.jsp?SpectrumID=CCMSLIB00004718271.
126. CCMSLIB00004705508 Available online: http://gnps.ucsd.edu/ProteoSAFe/gnpslibraryspectrum.jsp?SpectrumID=CCMSLIB00004705508.
127. CCMSLIB00004696576 Available online: http://gnps.ucsd.edu/ProteoSAFe/gnpslibraryspectrum.jsp?SpectrumID=CCMSLIB00004696576.

128. CCMSLIB00004705520 Available online: http://gnps.ucsd.edu/ProteoSAFe/gnpslibraryspectrum.jsp?SpectrumID=CCMSLIB00004705520.
129. CCMSLIB00004718285 Available online: http://gnps.ucsd.edu/ProteoSAFe/gnpslibraryspectrum.jsp?SpectrumID=CCMSLIB00004718285.
130. CCMSLIB00004683823 Available online: https://metabolomics-usi.ucsd.edu/spectrum/?usi=mzspec:GNPS:SUMNER:accession:CCMSLIB00004683823.
131. CCMSLIB00000846393 Available online: http://gnps.ucsd.edu/ProteoSAFe/gnpslibraryspectrum.jsp?SpectrumID=CCMSLIB00000846393.
132. Splash10-014i-0009000000-1f432f8a59ef5da38609 Available online: https://mona.fiehnlab.ucdavis.edu/spectra/display/MoNA_0003076.
133. CCMSLIB00005741694 Available online: http://gnps.ucsd.edu/ProteoSAFe/gnpslibraryspectrum.jsp?SpectrumID=CCMSLIB00005741694.
134. CCMSLIB00004718287 Available online: http://gnps.ucsd.edu/ProteoSAFe/gnpslibraryspectrum.jsp?SpectrumID=CCMSLIB00004718287.
135. Splash10-01q9-0104790000-552073d1029226254b2f Available online: https://mona.fiehnlab.ucdavis.edu/spectra/display/PR309363.
136. CCMSLIB00005777779 Available online: http://gnps.ucsd.edu/ProteoSAFe/gnpslibraryspectrum.jsp?SpectrumID=CCMSLIB00005777779.
137. CCMSLIB00005724857 Available online: http://gnps.ucsd.edu/ProteoSAFe/gnpslibraryspectrum.jsp?SpectrumID=CCMSLIB00005724857.
138. CCMSLIB00000081738 Available online: http://gnps.ucsd.edu/ProteoSAFe/gnpslibraryspectrum.jsp?SpectrumID=CCMSLIB00000081738.
139. CCMSLIB00000846994 Available online: http://gnps.ucsd.edu/ProteoSAFe/gnpslibraryspectrum.jsp?SpectrumID=CCMSLIB00000846994.
140. MSBNK-RIKEN-PR309108 Available online: https://massbank.eu/MassBank/RecordDisplay?id=MSBNK-RIKEN-PR309108&dsn=RIKEN.
141. CCMSLIB00004718157 Available online: http://gnps.ucsd.edu/ProteoSAFe/gnpslibraryspectrum.jsp?SpectrumID=CCMSLIB00004718157.
142. Lavault, M.; Richomme, P. Constituents of *Helichrysum Stoechas* Variety Olonnense. *Chemistry of Natural Compounds* **2004**, *40*, 118–121, doi:10.1023/B:CONC.0000033925.00693.7b.
143. CCMSLIB00010116988 Available online: http://gnps.ucsd.edu/ProteoSAFe/gnpslibraryspectrum.jsp?SpectrumID=CCMSLIB00010116988.
144. Garayev, E.; Herbette, G.; Di Giorgio, C.; Chiffolleau, P.; Roux, D.; Sallanon, H.; Ollivier, E.; Elias, R.; Baghdikian, B. New Sesquiterpene Acid and Inositol Derivatives from *Inula Montana* L. *Fitoterapia* **2017**, *120*, 79–84.
145. CCMSLIB00004683814 Available online: http://gnps.ucsd.edu/ProteoSAFe/gnpslibraryspectrum.jsp?SpectrumID=CCMSLIB00004683814.
146. CCMSLIB00004705562 Available online: http://gnps.ucsd.edu/ProteoSAFe/gnpslibraryspectrum.jsp?SpectrumID=CCMSLIB00004705562.

147. CCMSLIB00004705126 Available online: http://gnps.ucsd.edu/ProteoSAFe/gnpslibraryspectrum.jsp?SpectrumID=CCMSLIB00004705126.
148. Zhang, Z.; Jia, P.; Zhang, X.; Zhang, Q.; Yang, H.; Shi, H.; Zhang, L. LC–MS/MS Determination and Pharmacokinetic Study of Seven Flavonoids in Rat Plasma after Oral Administration of *Cirsium Japonicum DC.* Extract. *Journal of Ethnopharmacology* **2014**, *158*, 66–75, doi:10.1016/j.jep.2014.10.022.
149. Splash10-014i-1249000000-3b8220b7bf99c73cf79f Available online: https://mona.fiehnlab.ucdavis.edu/spectra/display/PR310830.
150. Guo, C.; Zhang, S.; Teng, S.; Niu, K. Simultaneous Determination of Sesquiterpene Lactones Isoalantolactone and Alantolactone Isomers in Rat Plasma by Liquid Chromatography with Tandem Mass Spectrometry: Application to a Pharmacokinetic Study. *Journal of Separation Science* **2014**, *37*, 950–956, doi:10.1002/jssc.201400119.
151. CCMSLIB00000847372 Available online: http://gnps.ucsd.edu/ProteoSAFe/gnpslibraryspectrum.jsp?SpectrumID=CCMSLIB00000847372.
152. MSBNK-NaToxAq-NA003096 Available online: https://massbank.eu/MassBank/RecordDisplay?id=MSBNK-NaToxAq-NA003096&dsn=NaToxAq.
153. CCMSLIB00004718251 Available online: http://gnps.ucsd.edu/ProteoSAFe/gnpslibraryspectrum.jsp?SpectrumID=CCMSLIB00004718251.
154. Wang, L.; Gan, C.; Wang, Z.; Liu, L.; Gao, M.; Li, Q.; Yang, C. Determination and Pharmacokinetic Study of Three Diterpenes in Rat Plasma by UHPLC-ESI-MS/MS after Oral Administration of Rosmarinus Officinalis L. Extract. *Molecules* **2017**, *22*, 934, doi:10.3390/molecules22060934.
155. HMDB0006547#spectra Available online: https://hmdb.ca/metabolites/HMDB0006547#spectra.
156. CCMSLIB00003137341 Available online: http://gnps.ucsd.edu/ProteoSAFe/gnpslibraryspectrum.jsp?SpectrumID=CCMSLIB00003137341.
157. Splash10-00lr-9210000000-41acd8a55f52afec6d79 Available online: https://hmdb.ca/spectra/ms_ms/1470932.
158. Splash10-0002-0090000000-3b4035f10b24d4c3f5a0 Available online: https://mona.fiehnlab.ucdavis.edu/spectra/display/PR309089.
159. Zanganeh, F.; Tayarani-Najaran, Z.; Nesměrák, K.; Štícha, M.; Emami, S.A.; Akaberi, M. Dereplication of Natural Cytotoxic Products from *Helichrysum Oligocephalum* Using Ultra-Performance Liquid Chromatography–Quadrupole Time of Flight-Mass Spectrometry. *SEPARATION SCIENCE PLUS* **2024**, *7*, 2300150, doi:10.1002/sscp.202300150.
160. CCMSLIB00003136330 Available online: http://gnps.ucsd.edu/ProteoSAFe/gnpslibraryspectrum.jsp?SpectrumID=CCMSLIB00003136330.
161. CCMSLIB00004684215 Available online: https://mona.fiehnlab.ucdavis.edu/spectra/display/CCMSLIB00004684215.
162. CCMSLIB00004708352 Available online: http://gnps.ucsd.edu/ProteoSAFe/gnpslibraryspectrum.jsp?SpectrumID=CCMSLIB00004708352.
163. Rizzato, G. Valutazione Della Qualità e Sicurezza in Prodotti Di Interesse Alimentare Attraverso Metodi Di Indagine Metabolomica, Università Ca'Foscari Venezia, 2018.
164. CCMSLIB00005467727 Available online: http://gnps.ucsd.edu/ProteoSAFe/gnpslibraryspectrum.jsp?SpectrumID=CCMSLIB00005467727.

165. HMDB0001388#spectra Available online: https://hmdb.ca/metabolites/HMDB0001388#spectra.
166. CCMSLIB00003137543 Available online: https://metabolomics-usi.ucsd.edu/spectrum/?usi=mzspec:GNPS:GNPS-NIST14-MATCHES:accession:CCMSLIB00003137543.
167. Taglialatela-Scafati, O.; Pollastro, F.; Chianese, G.; Minassi, A.; Gibbons, S.; Arunotayanun, W.; Mabebie, B.; Ballero, M.; Appendino, G. Antimicrobial Phenolics and Unusual Glycerides from *Helichrysum Italicum* Subsp. *Microphyllum*. *J. Nat. Prod.* **2013**, *76*, 346–353, doi:10.1021/np3007149.
